# Ciliopathy patient variants reveal organelle-specific functions for TUBB4B in axonemal microtubules

**DOI:** 10.1126/science.adf5489

**Published:** 2024-04-26

**Authors:** Daniel O Dodd, Sabrina Mechaussier, Patricia L Yeyati, Fraser McPhie, Jacob R Anderson, Chen Jing Khoo, Amelia Shoemark, Deepesh K Gupta, Thomas Attard, Maimoona A Zariwala, Marie Legendre, Diana Bracht, Julia Wallmeier, Miao Gui, Mahmoud R Fassad, David A Parry, Peter A Tennant, Alison Meynert, Gabrielle Wheway, Lucas Fares-Taie, Holly A Black, Rana Mitri-Frangieh, Catherine Faucon, Josseline Kaplan, Mitali Patel, Lisa McKie, Roly Megaw, Christos Gatsogiannis, Mai A Mohamed, Stuart Aitken, Philippe Gautier, Finn R Reinholt, Robert A Hirst, Chris O’Callaghan, Ketil Heimdal, Mathieu Bottier, Estelle Escudier, Suzanne Crowley, Maria Descartes, Ethylin W Jabs, Priti Kenia, Jeanne Amiel, Giacomo Maria Bacci, Claudia Calogero, Viviana Palazzo, Lucia Tiberi, Ulrike Blümlein, Andrew Rogers, Jennifer A Wambach, Daniel J Wegner, Anne B Fulton, Margaret Kenna, Margaret Rosenfeld, Ingrid A Holm, Alan Quigley, Emma A Hall, Laura C Murphy, Diane M Cassidy, Alex von Kriegsheim, Scottish Genomes Partnership, Genomics England Research Consortium, Network Undiagnosed Diseases, Jean-François Papon, Laurent Pasquier, Marlène S Murris, James D Chalmers, Claire Hogg, Kenneth A Macleod, Don S Urquhart, Stefan Unger, Timothy J Aitman, Serge Amselem, Margaret W Leigh, Michael R. Knowles, Heymut Omran, Hannah M Mitchison, Alan Brown, Joseph A Marsh, Julie P I Welburn, Shih-Chieh Ti, Amjad Horani, Jean-Michel Rozet, Isabelle Perrault, Pleasantine Mill

**Affiliations:** 1 MRC Human Genetics Unit, MRC Institute of Genetics and Cancer, University of Edinburgh, Edinburgh EH4 2XU, UK; 2 Laboratory of Genetics in Ophthalmology, INSERM UMR_1163, Institute of Genetic Diseases, Institut Imagine, Université de Paris, Paris 75015, France; 3 Department of Biological Chemistry and Molecular Pharmacology, Blavatnik Institute, Harvard Medical School, Boston 02215, USA; 4 School of Biomedical Sciences, The University of Hong Kong, Hong Kong SAR, China; 5 Respiratory Research Group, Molecular and Cellular Medicine, University of Dundee, Dundee DD1 9SY, UK; 6 Royal Brompton Hospital, London SW3 6NP, UK; 7 Department of Pediatrics, Washington University School of Medicine, St. Louis 63130, USA; 8 Wellcome Trust Centre for Cell Biology, School of Biological Sciences, University of Edinburgh, Edinburgh EH9 3BF, UK; 9 Department of Pathology and Laboratory Medicine, Marsico Lung Institute, University of North Carolina at Chapel Hill, Chapel Hill 27599-7248, USA; 10 Molecular Genetics Laboratory, Sorbonne Université, Assistance Publique - Hôpitaux de Paris (AP-HP), Hôpital Armand Trousseau, Paris 75012, France; 11 Sorbonne Université, INSERM, Childhood Genetic Disorders, Paris 75012, France; 12 Department of General Pediatrics, University Children’s Hospital Münster, Münster 48149, Germany; 13 Genetics and Genomic Medicine Department, UCL Institute of Child Health, University College London, London WC1N 1EH, UK; 14 Department of Human Genetics, Medical Research Institute, Alexandria University, Alexandria 21561, Egypt; 15 Faculty of Medicine, University of Southampton, Southampton SO16 6YD, UK; 16 Centre for Genomic and Experimental Medicine, MRC Institute of Genetics and Cancer, University of Edinburgh, Edinburgh EH4 2XU, UK; 17 South East of Scotland Genetics Service, Western General Hospital, Edinburgh EH4 2XU, UK; 18 Department of Anatomy, Cytology and Pathology, Hôpital Intercommuncal de Créteil, Créteil, France; 19 Biomechanics and Respiratory Apparatus, IMRB, U955 INSERM – Université Paris Est Créteil, CNRS ERL 7000, Créteil 94000, France; 20 MRC Prion Unit at UCL, UCL Institute of Prion Diseases, University College London, London W1W 7FF, UK; 21 Princess Alexandra Eye Pavilion, Edinburgh EH3 9HA, UK; 22 Center for Soft Nanoscience and Institute of Medical Physics and Biophysics, Münster 48149, Germany; 23 Biochemistry Division, Chemistry Department, Faculty of Science, Zagazig University, Ash Sharqiyah 44519, Egypt; 24 Core Facility for Electron Microscopy, Department of Pathology, Oslo University Hospital-Rikshospitalet, Oslo 0372, Norway; 25 Centre for PCD Diagnosis and Research, Department of Respiratory Sciences, University of Leicester, Leicester LE1 9HN, UK; 26 Department of Medical Genetics, Oslo University Hospital, Oslo 0407, Norway; 27 Paediatric Department of Allergy and Lung Diseases, Oslo University Hospital, Oslo 0407, Norway; 28 Department of Genetics, University of Alabama at Birmingham, Birmingham, 35294-0024, USA; 29 Icahn School of Medicine at Mount Sinai, New York 10029-6504, USA; 30 Department of Clinical Genomics, Mayo Clinic, Rochester 55905, USA; 31 Department of Paediatric Respiratory Medicine, Birmingham Women’s and Children’s Hospital NHS Foundation Trust, Birmingham B15 2TG, UK; 32 Département de Génétique, Hôpital Necker-Enfants Malades, Assistance Publique Hôpitaux de Paris (AP-HP), Paris 75015, France; 33 Laboratory of Embryology and Genetics of Human Malformations, INSERM UMR 1163, Institut Imagine, Université de Paris, Paris 75015, France; 34 Pediatric Ophthalmology Unit, Meyer Children's Hospital IRCCS, Florence 50139, Italy; 35 Pediatric Pulmonary Unit, Meyer Children's Hospital IRCCS, Florence 50139, Italy; 36 Medical Genetics Unit, Meyer Children's Hospital IRCCS, Florence 50139, Italy; 37 Carl-Thiem-Klinikum Cottbus, Cottbus 03048, Germany; 38 Department of Ophthalmology, Boston Children’s Hospital; Boston 02115, USA; 39 Department of Otolaryngology, Boston Children’s Hospital; Boston 02115, USA; 40 Department of Pediatrics, University of Washington School of Medicine and Seattle Children’s Research Institute, Seattle 98015, USA; 41 Division of Genetics and Genomics and the Manton Center for Orphan Diseases Research, Boston Children’s Hospital, Boston 02115, USA; 42 Department of Pediatrics, Harvard Medical School, Boston 02115, USA; 43 Department of Paediatric Radiology, Royal Hospital for Children and Young People, Edinburgh EH16 4TJ, UK; 44 Cancer Research UK Edinburgh Centre, Institute of Genetics and Cancer, University of Edinburgh, Edinburgh EH4 2XU, UK; 45 Genomics England, William Harvey Research Institute, Queen Mary University of London, London EC1M 7AA, UK; 46 Undiagnosed Diseases Network Coordinating Center, Harvard Medical School, Boston 02115, USA; 47 ENT Department, Bicêtre Hospital, Assistance Publique-Hôpitaux de Paris (AP-HP), Paris-Saclay University, Le Kremlin-Bicêtre 94270, France; 48 Medical Genetics Department, CHU Pontchaillou, Rennes 35033, France; 49 Department of Pulmonology, Transplantation, and Cystic Fibrosis Centre, Larrey Hospital, Toulouse 31400, France; 50 Department of Paediatric Respiratory and Sleep Medicine, Royal Hospital for Children and Young People, Edinburgh EH16 4TJ, UK; 51 Department of Child Life and Health, University of Edinburgh, Edinburgh EH16 4TJ, UK; 52 Department of Pediatrics, Marsico Lung Institute, University of North Carolina at Chapel Hill, Chapel Hill 27599-7248, USA; 53 Department of Medicine, Marsico Lung Institute, University of North Carolina at Chapel Hill, Chapel Hill 27599-7248, USA; 54 Department of Cell Biology and Physiology, Washington University School of Medicine, St. Louis 63110, USA

## Abstract

Tubulin, one of the most abundant cytoskeletal building blocks, has numerous isotypes in metazoans encoded by different conserved genes. Whether these distinct isotypes form cell-type and context-specific microtubule structures is poorly understood. Based on a cohort of 12 patients with primary ciliary dyskinesia, as well as mouse mutants, we identified and characterized variants in the *TUBB4B* isotype that specifically perturbed centriole and cilium biogenesis. Distinct *TUBB4B* variants differentially affected microtubule dynamics and cilia formation in a dominant-negative manner. Structure-function studies revealed that different TUBB4B variants disrupted distinct tubulin interfaces, thereby enabling stratification of patients into three classes of ciliopathic diseases. These findings illustrate that specific tubulin isotypes have unique and non-redundant subcellular functions and establish a link between tubulinopathies and ciliopathies.

The dynamic remodeling of microtubules drives diverse cellular processes from organelle trafficking and chromosome segregation to templating stable structures such as centrioles and ciliary axonemes. Microtubules are highly conserved polymers composed of α- and β-tubulin heterodimers. While organisms like *Chlamydomonas reinhardtii* have only one form of α- and β-tubulin, humans possess ten β-tubulin isotypes, each of which can potentially pair with any one of nine α-tubulin isotypes. Single cell sequencing data show that tubulin isotypes display both unique and overlapping expression patterns across cell types and developmental stages. Different genes each encoding a distinct tubulin isotype provide metazoans with discrete transcriptional modules to meet shifting demand for tubulin subunits across development and cell type (1–3). Mutations in genes encoding tubulins cause tubulinopathies, a broad spectrum of predominantly neurological conditions and neurodegenerative disorders (4). Differences in coding sequence between isotypes within species could alter the physical properties of microtubules they are incorporated in, thereby supporting functional specialization (5–8). This idea underlies the proposed concept of a ‘tubulin code,’ in which expression of a given set of isotypes, combined with specific post-translational modifications, could dictate the stability and mechanical properties of the microtubule lattices they form (3). Whether biological context-specific subcellular or organelle-specific lattices actually exist remains to be clearly demonstrated. One area of exploration is within subcellular compartments that exhibit distinct microtubule architecture.

The cilium is one such subcellular compartment. Cilia are microtubule-based organelles essential to embryonic development and required postnatally across critical physiological processes, including vision, hearing, olfaction, respiration, excretion and reproduction. Although different cilia types may vary in their final structure, function, size and number, they share certain conserved elements. In all cilia, microtubules are arranged into an axoneme, an axial structure consisting of nine microtubule doublets. This arrangement is templated by the basal body (a modified centriole), in which microtubules are organized into nine triplets. Tubulin heterodimers are polymerized into protofilaments that are radially interlinked within the microtubule doublets and lengthened longitudinally at the cilia tip. Unlike other cytoskeletal networks in a cell, the microtubules of axonemes are comparatively stable (9, 10), particularly in motile cilia, which power fluid flow and thus require considerable mechanical strength to permit constant motor-driven bending (11, 12). While conventional transmission electron microscopy (TEM) suggests that these microtubule-based structural elements are similar across basal bodies and axonemes, it remains unknown whether axoneme assembly and function utilize specific tubulin isotypes.

Mutations in over 200 genes that affect cilia structure and/or function result in a list of over 40 conditions termed ciliopathies (13, 14). These can be roughly divided into sensory and motile ciliopathies. Sensory ciliopathies result from impaired signaling functions of non-motile primary cilia. They are associated with a spectrum of diseases, ranging from lethal multiorgan syndromes to non-syndromic forms like retinal dystrophy, which impact only a specific organ. Motile ciliopathies affect the ability of motile cilia to generate effective fluid flow (15). This results in heterogeneous clinical manifestations, which include defects in airway mucociliary clearance and hydrocephaly from accumulation of cerebrospinal fluid (CSF) in brain ventricles. The molecular basis for the clinical heterogeneity observed amongst ciliopathy patients – even within one type of condition – remains unclear.

Primary ciliary dyskinesia (PCD, OMIM: PS244400) is a motile ciliopathy affecting the structure and function of motile cilia that line the airways, the brain ependyma, the reproductive tracts and the transient embryonic node. In patients with PCD, these cilia are static, beat in an uncoordinated manner, or are completely absent (ciliary agenesis). These ciliary defects can result in chronic respiratory disease, owing to impaired mucociliary clearance, as well as laterality defects, hydrocephaly and infertility in a subset of patients (16, 17). Syndromic (*i.e*. with additional sensory ciliopathy features) PCD is very rare (18, 19). PCD patients almost exclusively present with respiratory features with or without involvement of other motile ciliated tissues. Most PCD cases are recessively inherited, owing to variants in ^~^50 genes (20). However, mutations in these genes only account for ^~^70% of PCD cases, indicating that additional unidentified causal genes likely exist.

Here, we identified mutations in the β-tubulin isotype *TUBB4B* associated with a subgroup of PCD. These mutations impacted different functional interfaces of the tubulin protein and resulted in distinct presentations of ciliopathic disease, where some showed only PCD phenotypes and others exhibited a syndromic ciliopathy. Through computational analysis, cell-based structure-function analysis, and mouse knockout studies, we defined the role of TUBB4B in ciliary function and the effect of disease-associated mutations on microtubule dynamics and axoneme assembly.

## Identification of de novo heterozygous *TUBB4B* variants in PCD cases

To examine the molecular basis of PCD in a cohort of 8 clinically diagnosed patients, we undertook trio whole genome sequencing (WGS)(21). We identified by ultrastructural analysis a patient P1 (HG-003) exhibiting ciliary agenesis, sometimes referred to as reduced generation of multiple motile cilia (RGMC), a specific subtype of PCD. This patient also had shunted hydrocephalus. Hydrocephalus in human patients with PCD is rare but occurs most commonly through recessive inheritance in genes associated with the RGMC phenotype such as *CCNO* and *MCIDAS*, or through heterozygous dominant de novo mutations in the master motile ciliogenesis transcriptional regulator *FOXJ1*. However, no pathogenic or potentially pathogenic gene variants have been identified in any of these genes (22–24) or in other known PCD genes.

**Figure 1:**
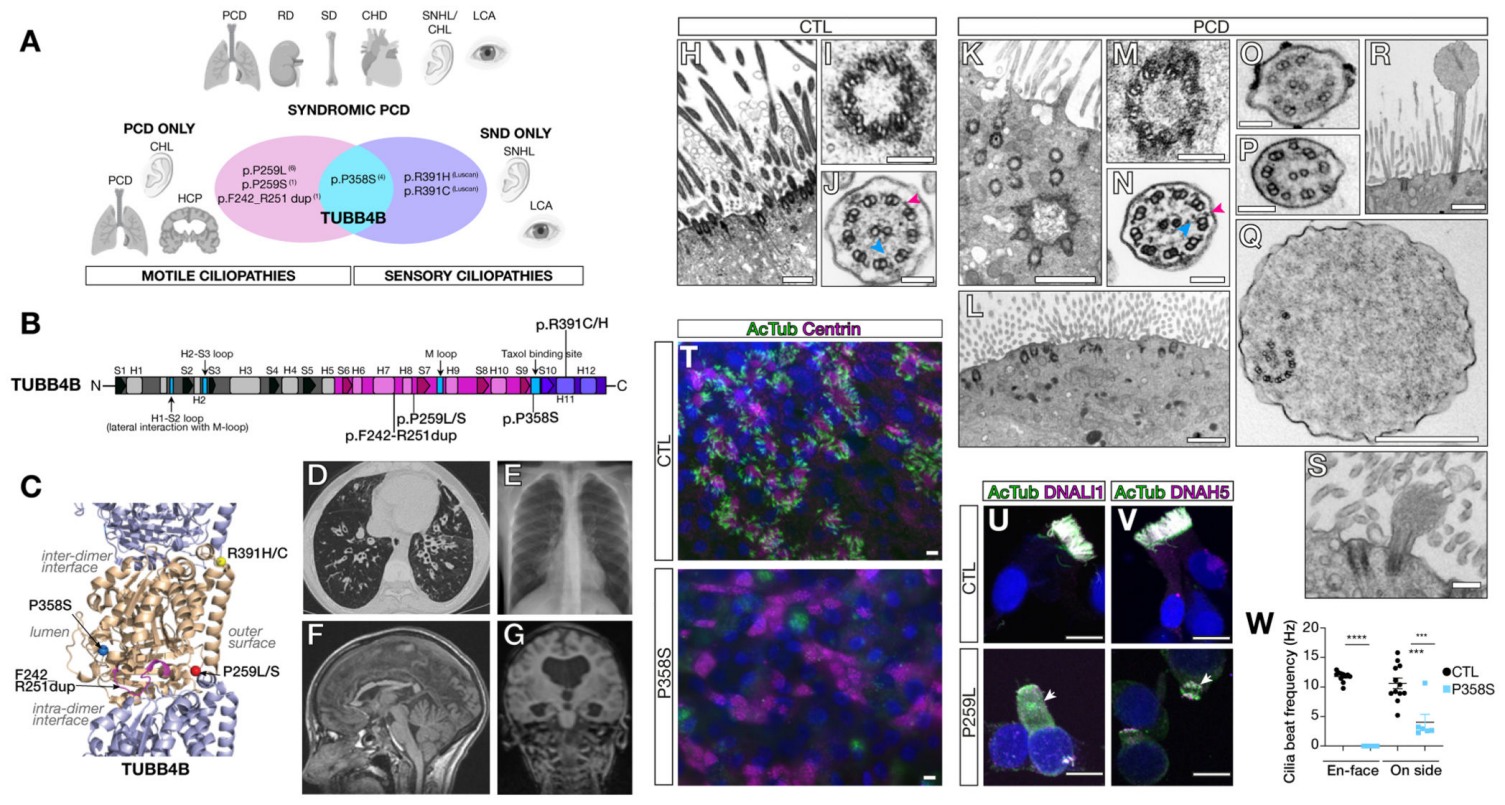
Distinct de novo *TUBB4B* variants caused PCD-only, SND-only or syndromic (PCD+SND) disease. (**A**) Schematic of patient phenotypes within the ciliopathic spectrum clustered on genotypes. Abbreviations: CHL: conductive hearing loss; CHD: congenital heart disease; HCP: hydrocephaly; LCA: Leber congenital amaurosis; PCD: primary ciliary dyskinesia; RD: renal disease; SD: skeletal defects; SNHL: sensorineural hearing loss; SND: sensory-neural disorder. (**B,C**) Resulting changes mapped onto a protein schematic (**B**) or an atomic model of TUBB4B (gold) with TUBA1 (purple) (**C**). (**D-G**) Clinical features of PCD patients: (**D**) chest CT showing bilateral lower lobe bronchiectasis (P1); (**E**) X-ray showing right middle lobe atelectasis (P2); (**F**) midline T1 sagittal image showing irregular corpus callosum secondary to earlier hydrocephaly (shunted), no evidence of basal ganglia dysmorphology is observed, typical of tubulinopathies (P1); (**G**) MRI showing dilated ventricles (P8). (**H-S**) TEM of healthy donor (**H-J**) and PCD patient nasal epithelia (**K-S**). Patient samples showed misoriented, internally docked (**K**) or reduced centrioles without axonemes (**L**) (P3), incomplete microtubule triplets (P3) (**M**), and rare intact axoneme (P9) with both inner (blue arrowheads) and outer (magenta arrowheads) dynein arms (**N**). Missing doublets (**O**, P3), singlet microtubules (**P**, P3), disrupted axonemes (Q, P3) or rare short axonemes with bulbous tips (**R**, P3; **S**, P10) were observed. (**T**) Wholemount immunofluorescence of nasal epithelial cultures from unaffected parent and patient (P9). (**U,V**) Immunofluorescence of healthy donor or patient (P2) cells for cilia axonemes and dynein motor proteins. See Fig. S2L,M. (**W**) Cilia beat frequency of control and patient (P9) airway cultures. Mean ± SEM from N=3 experimental replicates. Student’s t-test: ***, p ≤ 0.001; ****, p ≤ 0.0001. Scale bars: 10 μm (**T-V**); 1 μm (**H,K,L,R**); 500 nm (**Q**); 125 nm (**I,M**), 200 nm (**S**) and 100 nm (**J,N-P**).

PCD is largely inherited in an autosomal recessive manner except for one recent example of autosomal dominant inheritance (*FOXJ1*) and a few cases of X-linked recessive inheritance (*RPGR, PIH1D3, OFD1*)(20). Unlike the other seven patients, whom we molecularly diagnosed as carrying biallelic variants in known PCD genes, we found that patient P1 carried a de novo missense mutation – p.P259L (chr9:g.137242994:C>T (hg38)) – in the *TUBB4B* gene encoding the β-tubulin 4B isotype (Fig. S1A). We then identified an additional cohort of eleven unrelated PCD patients with heterozygous, often recurrent, variants in *TUBB4B*. Of these, five patients carried p.P259L, one patient carried p.P259S (chr9:g.137242993:C>T (hg38)), one patient carried an in-frame ten amino acid duplication p.F242_R251dup (chr9: g.137242941_137242970dup (hg38)) and four patients carried p.P358S (chr9:g.137243290:C>T (hg38)) (Fig. 1A-C, Fig. S1). Common clinical features of airway disease including bronchiectasis were observed across the cohort (Fig. 1D,E). In addition, 6/12 patients exhibited the less-commonly associated feature of hydrocephaly (Fig. 1F,G, Tables S1,S2). Laterality defects were uncommon, observed in only 1/12 patients. 8/12 patients presented with PCD only (PCD-only group: p.P259L, p.P259S, p.F242_R251dup). In comparison, the four patients with the p.P358S substitution also presented with Leber congenital amaurosis (LCA) associated with sensorineural hearing loss (SNHL)- a syndromic phenotype (PCD+SND group). Two out of these four p.P358S substitution patients also exhibited renal defects (RD), congenital heart defects (CHD) or skeletal growth defects (SD), suggesting defects in several additional ciliated tissues. These phenotypes were all distinct from that of sensory-neural disease (SND-only) previously reported to be linked to the recurrent *TUBB4B* missense variants p.R391H or p.R391C across four unrelated families (25). These patients were characterized by early-onset and severe retinal dystrophy (EOSRD/LCA) associated with sensorineural hearing loss (SNHL). Importantly, no rhinopulmonary features characteristic of PCD airway dysfunction were reported for these original four families. These findings taken together suggested that dominant mutations in *TUBB4B* can cause three distinct and separate clinical presentations: a solely motile ciliopathy (PCD-only), a solely sensory ciliopathy (SND-only) and a syndromic form impacting both motile and sensory cilia (PCD+SND).

## *TUBB4B* mutations disrupted cilia and centrosomes in patient respiratory cells

Regardless of genotype, we observed similar cellular phenotypes in PCD *TUBB4B* patient-derived respiratory epithelial cells. These phenotypes included reduced numbers of apically docked basal bodies, basal bodies that fail to extend an axoneme (Fig. 1H-L, Fig. S2A-D), and incomplete centriole microtubule triplets (Fig. 1K,M, Fig. S2C’-F). Axonemes that did extend were short and had bulbous tips displaying disrupted and misoriented microtubules (Fig. 1O-S, Fig. S2G-J’, Movie S1). To confirm the ciliary agenesis phenotype, we expanded and differentiated control and patient respiratory epithelial cultures from nasal brushings. Patient cells in culture recapitulated poor ciliation and reduced number of basal bodies (Fig. 1T, Fig. S2K).

PCD is most commonly caused by mutations that disrupt the expression and assembly of the axonemal dynein motors that power cilia beating (20, 26). We observed by immunofluorescence in *TUBB4B* patient cells mislocalization of dynein motors either to the cytoplasm or to the apical region where cilia should have formed (Fig. 1U,V, Fig. S2L). Furthermore, acetylated α-tubulin, which normally marks axonemes, appeared as cytoplasmic aggregates (Fig. 1U,V, Fig. S2K, arrowheads). These results suggested that axonemal motors were still produced, even in the absence of axonemes. Indeed, the rare axonemes that did form had inner and outer dynein arms (Fig. 1J,N), albeit with reduced motility (Fig. 1W, Movie S2-S6). Post-translational modifications of tubulin are a key part of the tubulin code and are normally common on ciliary microtubules. In contrast, patient cells showed alterations of these marks on the rare cilia observed with apical cytoplasmic accumulations (Fig. 1U,V, Fig. S2K-M). These defects in centriole amplification, axoneme extension, and tubulin modification may underlie the defects in mucociliary clearance observed in patients.

## *TUBB4B* is essential for motile cilia assembly in specific tissues

**Figure 2:**
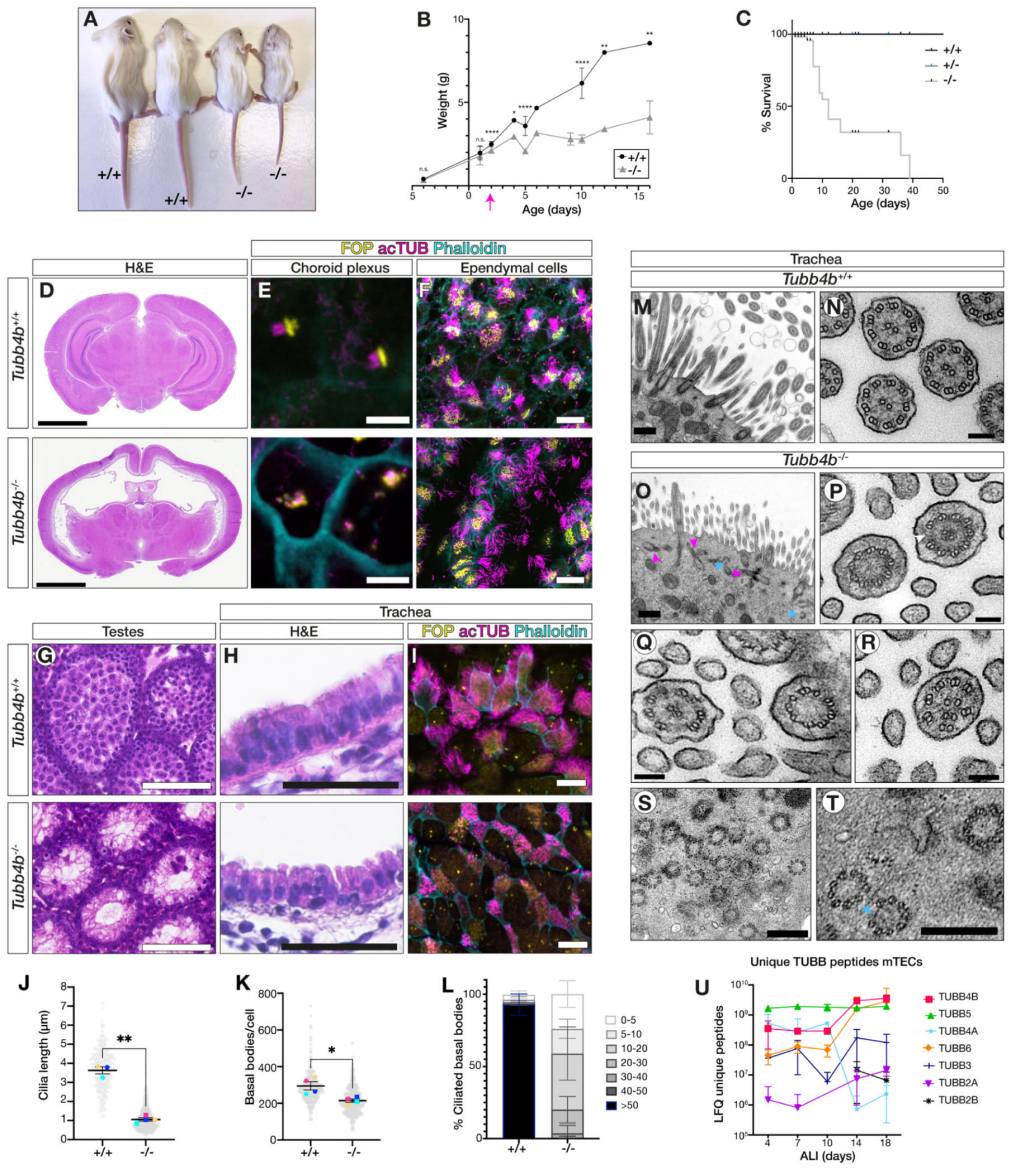
*Tubb4b* was specifically required for axoneme formation in motile ciliated tissues in vivo. (**A-C**) *Tubb4b*^*-/-*^ animals exhibited postnatal runting (**A**, P16), starting from P2 (**B**), and postnatal lethality (**C**). (**D-F**) *Tubb4b*^*-/-*^ animals displayed hydrocephaly (**D**, P15). Choroid plexus multicilia were disrupted (**E**, P5) while ependymal cilia were largely normal (**F**, P5). (**G-I**) *Tubb4b*^*-/-*^ animals exhibited male infertility and spermatogenesis defects (**G**, P22) and severe disruption of tracheal cilia by histology (**H**) and immunofluorescence (**I**). (**J-L**) Quantification of wholemount immunofluorescence of *Tubb4b;Centrin2-eGFP* neonatal (P5-P8) trachea cilia length (**J**), basal body number per cell (**K**) and percentage of ciliated basal bodies per cell (**L**). See Fig. S3G. N = 4 animals per genotype with (**J**) n >75 cilia per biological replicate and (**K,L**) n > 56 cells per biological replicate. (**M-T**) TEM from control (**M,N**) and *Tubb4b*^*-/-*^ neonatal (P1-P4) mutant tracheas (**O-T**). Mutants showed non-docked centrioles without axonemes (magenta arrowheads) and partial centrioles (cyan arrowheads) (**O**). Missing microtubule doublets (**P**) (white arrowhead), missing central pair apparatus (**R**) and microtubule singlets with disrupted organization (**Q**). Within the mutant cytosol, partial centrioles and centrioles with microtubule doublets instead of triplets (**S,T**). (**U**) Mass spectrometry of differentiating control mTECs detected many unique β-tubulins. Scale bars: 2.5 mm (D), 5 μm (E), 20 μm (F), 100 μm (G), 50 μm (H), 10 μm (I), 500 nm (M,O), 100 nm (N,P,Q,R) and 300 nm (S,T). (B,J,K) Graphic bars: mean ± SEM derived from N>3 animals per time point. Student’s t-test ns, not significant; *, p ≤ 0.1; **, p ≤ 0.01; ***, p ≤ 0.001; ****, p ≤ 0.0001. (U) Line chart: mean ± SEM derived from N=3 experimental replicates per time point.

To investigate the requirement for TUBB4B in vivo, we generated *Tubb4b*^*-/-*^ homozygous protein null mice (Fig. S3A-C). *Tubb4b*^*-/-*^ mice were born at Mendelian ratios but exhibited perinatal lethality with runting (Fig. 2A-C) and hydrocephaly (Fig. 2D), both features associated with motile cilia dysfunction in murine models. Consistent with the lack of prominent laterality defects amongst our *TUBB4B* patient cohort (1/12 patients with dextrocardia), we did not observe any left-right patterning defects in the mice. We observed defects in surviving male spermatogenesis (Fig. 2G) and defects in oviduct multiciliated cells, which exhibited reduced cilia lengths (Fig. S4A-C). The lack of overt skeletal or growth phenotypes at birth in *Tubb4b*^*-/-*^ neonates (Fig. 2B) suggested that TUBB4B is not required for embryonic development, where primary cilia play key roles. Indeed, *Tubb4b*^*-/-*^ cilia in primary fibroblasts showed normal numbers and lengths (Fig. S3D-F).

To examine the effects of mutations on motile cilia function, we first evaluated the hydrocephalus phenotype. This phenotype can be caused by defects in motile cilia on ependymal cells, which generate CSF flow. Given that 75% of the PCD-only cohort of *TUBB4B* patients also had hydrocephaly, we expected to see defects in the multiciliated ependymal cells lining the ventricles. However, although *Tubb4b*^*-/-*^ mice exhibited pronounced and progressive dilatations of the ventricles neonatally without obstruction of aqueducts that suggested communicating hydrocephalus (Fig. 2D), motile cilia on ependymal cells exhibited grossly normal lengths and densities (Fig. 2F, Fig. S4D-F). Instead, we observed profound reductions in cilia number and length in choroid plexus cells involved in CSF secretion and regulation (Fig. 2E). Ependymal cilia further examined ex vivo (Fig. S4G-I) confirmed that the cilia numbers and lengths were grossly normal, and also showed that there was no significant difference in ciliary beat patterns or frequency. These data emphasized that, despite the similarities in molecular cascades driving multiciliogenesis between tissue types in mammals, lack of TUBB4B did not cause overt ependymal ciliary defects as it does in the adjacent choroid plexus epithelial cells.

We also observed defects in both the number and length of *Tubb4b*^*-/-*^ tracheal cilia (Fig 2H-L, Fig. S3G). *Tubb4b*^*-/-*^ centrioles also failed to amplify and exhibited partially formed microtubule triplets. Despite these defects, some fully formed basal bodies managed to dock and extend rare, stumpy axonemes (Fig. 2L-T). These phenotypes (25) have been confirmed by a recent publication on an additional *Tubb4b* allele (27). On examining serial sections, we observed axonemal defects including the loss or duplication of central pairs, loss of microtubule doublets and microtubule disorganization arising at or just proximal to the transition zone (Fig. S5). Notably, in the absence of TUBB4B, other cytoskeletal processes looked grossly normal including apical-basal patterning in the pseudostratified epithelium. These data suggested a unique role for TUBB4B as a critical ‘limiting component’ specific for organelle size control and scaling in airway epithelial cell cilia.

We performed proteomic analysis of wild-type tracheal cultures at different timepoints across airway epithelial differentiation (e.g. air-liquid interface day 4 (ALI4): centriole amplification, ALI10: early ciliogenesis) and observed that multiple different β-tubulin isotypes were expressed during the process, as indicated by unique peptide reads (Fig. 2U). Together with our knockout data, this suggested that although other β-tubulins in multiciliated airway cells are expressed during developmental timepoints when centrioles and cilia were built, they are non-redundant with TUBB4B. TUBB4B therefore fulfils a unique role in ciliogenesis and is essential for the formation of multiple motile cilia in the respiratory epithelium. These data further support the idea that TUBB4B is a cilia-specific tubulin.

## TUBB4B variants differentially impacted microtubule dynamics and tubulin heterodimer formation

**Figure 3:**
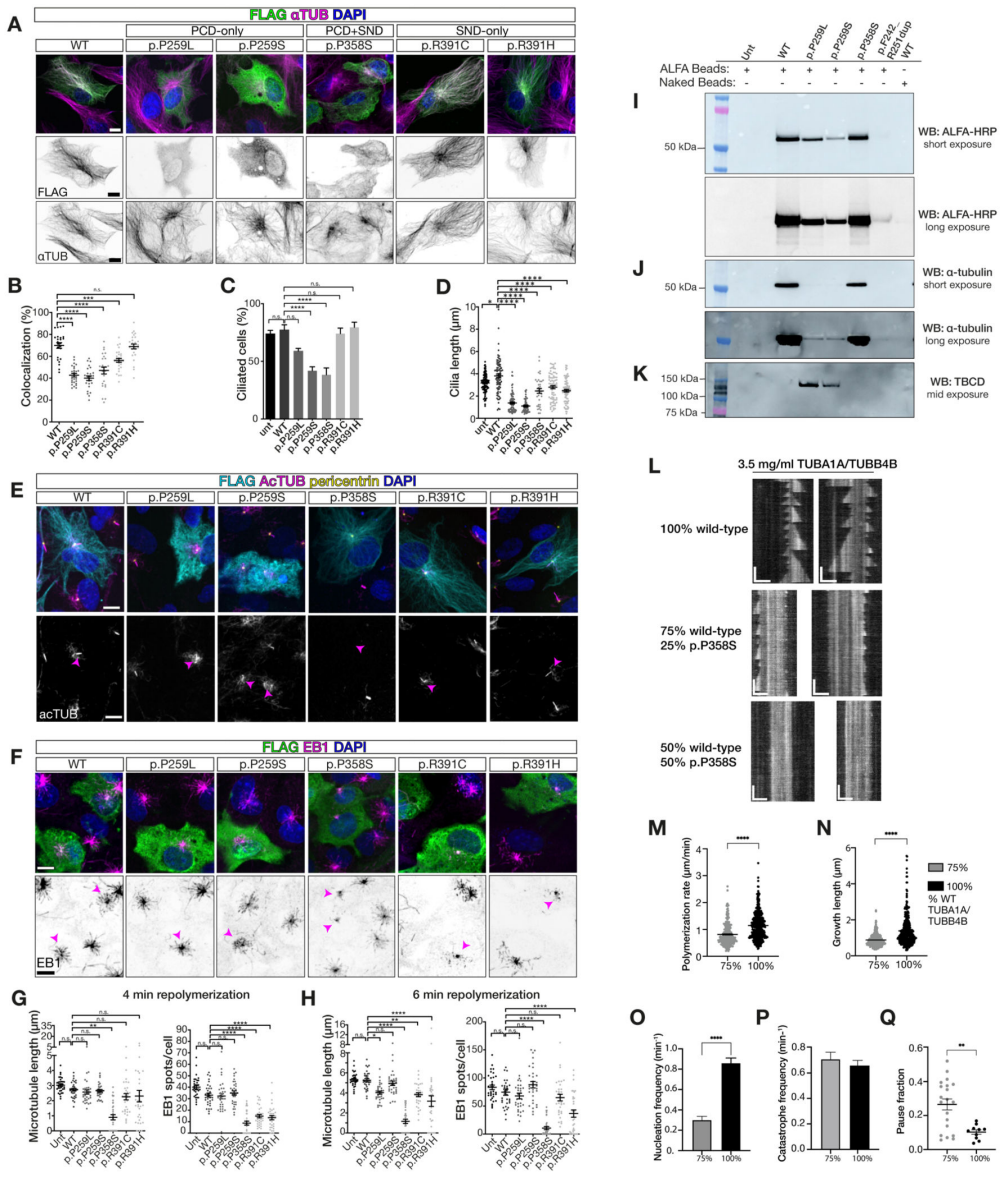
Disease-causing TUBB4B variants altered microtubule dynamics and ciliation. (**A**) Microtubule incorporation of wild-type (WT), p.P259L, p.P259S, p.P358S, p.R391C and p.R391H TUBB4B variants overexpressed in RPE1 cells. (**B**) Quantification of percentage colocalization of FLAG TUBB4B variants and α-tubulin staining. (**C-E**) Ciliogenesis in 24 h serum-starved RPE1 cells overexpressing TUBB4B variants. Acetylated α-tubulin only channel (lower panel) with transfected cell cilia highlighted by magenta arrowhead. Quantification of (**D**) rates of ciliation and (**E**) cilia lengths. (**F**) Microtubule dynamics analysis of RPE1 cells overexpressing TUBB4B variants, upon repolymerization at 37 °C for 4 min. Inverted EB1 only channel (lower panel) in transfected cell highlighted by magenta arrowhead to illustrate variant effects on dynamics. (**G,H**) Quantification upon repolymerization at 4 (**G**) and 6 (**H**) min. See also Fig. S6. (**I-K**) Affinity purified (ALFA beads) lysates from stable IMCD3 cells expressing ALFA-tagged human *TUBB4B* variants immunoblotted against ALFA (**I**), α-tubulin to examine heterodimer assembly (**J**) and TBCD, a heterodimer assembly pathway chaperone (**K**). (**L**) TIRF microscopy time-lapse image kymographs microtubule growth dynamics from GMPCPP-stabilized wild-type seeds with varying concentrations of p.P358S containing purified tubulin TUBA1-TUBB4B heterodimers (Horizontal scale bar = 3 μm, vertical scale bar = 2.27 min). (**M-Q**) Data distribution and statistical analysis of microtubule-polymerization rate (**M**), growth length (**N**), nucleation frequency (**O**), and fraction of time for each microtubule that paused during polymerization (**Q**) with addition of p.P358S. Catastrophe frequency (**P**) is not affected. Kinetic analysis was not performed for the 50:50 as no dynamics were observed. See also Fig. S8. Scale bars: (A, E, F) 10 µm. Plots represent the mean ± SEM, N= 2 (B-D,G,H) or 3 (M-Q) experimental replicates. Ns, not significant; *, p ≤ 0.1; **, p ≤ 0.01; ***, p ≤ 0.001; ****, p ≤ 0.0001.

In order to understand how different *TUBB4B* mutations might affect microtubule dynamics and ciliation, we transiently overexpressed human FLAG-tagged wild-type and disease-associated variants of TUBB4B in RPE-1 cells (Fig. 3, Fig. S6). PCD-only TUBB4B variants (p.P259L/S) failed to colocalize strongly to microtubules (Fig. 3A,B) and the PCD+SND syndromic variant (p.P358S) showed reduced colocalization. In contrast, microtubule localization was minimally affected for the SND-only variants (p.R391H/C).

Under serum-starvation conditions to induce ciliogenesis, we also examined effects of TUBB4B variants on cilia length and numbers (Fig. 3C-E). We measured the kinetics of microtubule depolymerization in cells expressing these different TUBB4B variants by tracking the number and lengths of microtubules bound to the end-binding protein EB1 after cold shock followed by repolymerization (Fig. S6). PCD-only TUBB4B variants (p.P259L/S), which showed low incorporation into MTs including those of the centrosome, had no observable effects on cytoplasmic microtubule dynamics (Fig. 3F-H) but profoundly decreased cilia number and length (Fig 3C-E). In contrast, the syndromic PCD+SND TUBB4B variant (p.P358S) localized to centrosomes upon repolymerization but strongly impeded the number and length of repolymerizing cytoplasmic microtubules, as well as decreased the number and length of cilia (Fig. 3C-H). The SND-only variants (p.R391H/C) showed intermediate effects on microtubule dynamics, and only modestly affected the length of primary cilia (Fig. 3C-H). Overexpression of wild-type TUBB4B only slightly increased cilia length without disrupting rates of ciliation or microtubule dynamics, suggesting that the effects observed of the variants are unlikely to be caused by overexpression alone (Fig. 3C-H). Together, these findings suggested that each variant acts to disrupt microtubule biology via differing mechanisms.

To gain insight into the molecular mechanisms by which different TUBB4B variants disrupt tubulin function, we first focused on the chaperone-dependent α/β tubulin heterodimer assembly pathway (Fig. S7A) using in vitro transcription/translation products (Fig. S7B,C) and stable cell lines expressing control and TUBB4B variants (Fig. 3I,J, Fig. S7D). While all constructs were equally translated in vitro (Fig. S7B), the three PCD-only variants (p.P259L, p.P259S, Dup) in mammalian cells and in vitro significantly impaired α/β heterodimer formation (Fig. 3I,J, Fig. S7C). Moreover, p.P259L and p.P259S did not bind α-tubulin, instead co-purifying with TBCD (tubulin folding co-factor D: step 4), one of the five co-chaperones required for assembly and disassembly of the α/β-tubulin heterodimers (Fig. 3K, Fig. S7C). This suggests that these PCD-only variants disrupted the α/β heterodimer assembly pathway.

The p.P358S variant, however, did not show disrupted binding to α-tubulin (Fig. 3I,J, Fig. S7C). To further determine how p.P358S impacts tubulin function, we purified in vitro recombinant human TUBB4B co-expressed with TUBA1A. Both wild type and p.P358S TUBB4B formed heterodimers with TUBA1A robustly in this system (Fig. S8A,B,C). In the presence of a slowly hydrolyzable GTP analogue, guanylyl-(α, β)-methylene-diphosphonate (GMPCPP), wild-type TUBA1A-TUBB4B heterodimers polymerized into micrometer-long microtubules. However, p.P358S mutant TUBB4B required both GMPCPP and taxol, which stabilizes microtubules, to form microtubules. This indicated that p.P358S-containing TUBA1A-TUBB4B heterodimers were capable of forming a microtubule lattice but required a higher critical concentration to do so (Fig. S8D). To test the effects of p.P358S on microtubule dynamics, we undertook total internal reflection fluorescence (TIRF) videomicroscopy on wild-type seed microtubules with varying ratios of isotypically pure tubulin TUBA1A-TUBB4B heterodimers containing wild-type and p.P358S variants (Fig. 3L-Q). Mutant TUBB4B was able to potently inhibit wild-type microtubule dynamics in a dose-dependent manner in vitro, similar to what we observed in our cellular experiments (Fig. 3F,G). We observed significant decreases in the growth characteristics of p.P358S containing microtubules (i.e. polymerization rate (Fig. 3M), growth (Fig. 3N), nucleation frequency (Fig. 3O)) and increases in the duration of pause during polymerization (Fig. 3Q), events without sustained microtubule growth or shrinkage.

Together these findings demonstrated how PCD-causing *TUBB4B* mutations disturbed centriole number and axoneme size by disrupting heterodimer assembly (PCD-only variants) or disrupting polymerization (PCD+SND) in an organelle-specific manner.

## PCD-associated mutations have a dominant-negative effect in mouse and patient cell models

**Figure 4:**
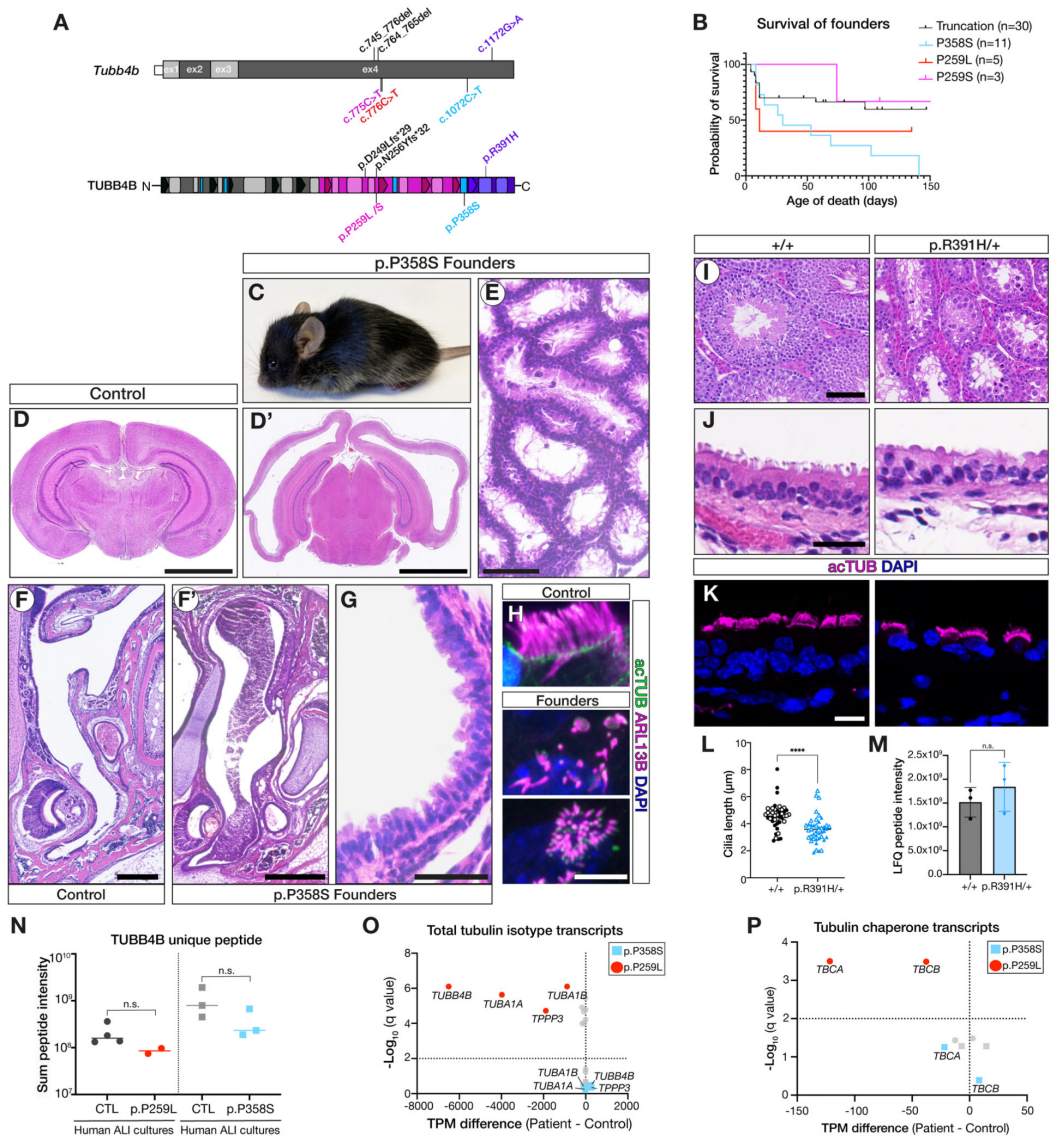
*TUBB4B* variants caused dominant disease in vivo. (**A**) Schematic of patient p.P259L/S (PCD-only, red or magenta), p.P358S (syndromic PCD+LCA, blue) and p.R391H (SND-only, purple) mutations and truncating mutations (p.D249Lfs*29 and p.N256Yfs*32, null alleles (black)) on the mouse *Tubb4b* mRNA (top) and protein (bottom). (**B-H**) (B) Kaplan-Meier graph of founder mice carrying PCD-patient variants which died either spontaneously or were euthanized for health concerns. (C-H) p.P358S founders exhibited hydrocephaly (C,D’), defects in spermatogenesis (E), mucopurulent nasal plugs (F’), and loss of tracheal cilia (G,H). BL/6 age-matched controls (D,F). (**I-M**) *Tubb4b*^*R391H/+*^ animals showed no decrease in fitness or survival allowing a line to be established (see also Fig. S9). Males were infertile with defects in spermatogenesis (I). *Tubb4b*^*R391H/+*^ tracheal cilia histology (J), immunofluorescence, length quantified in (L). Mass spectrometry of trachea quantifying TUBB4B levels (M). (**N-P**) Nasal epithelial cultures from healthy controls, a PCD-only patient (red, p. P259L) or syndromic PCD+SND patient (blue, p.P358S) were used for mass spectrometry of unique TUBB4B peptides (N) or targeted RNASeq analysis of tubulin (O) or tubulin chaperone (P) transcripts. Scale bars: 2.5 mm (D), 500 μm (E), 250 μm (F), 100 μm (I), 50 μm (G), 25 μm (J) and 10 μm (H,K). (L, M) Graphic bars: mean ± SEM from N=2 biological replicates, n>20 cells/replicate (L) and N= 3 biological (M) or experimental (N) replicates. Student’s t-test: ns, not significant; ****, p ≤ 0.0001.

If, as proposed, certain mutant TUBB4B variants can act in a dominant-negative manner in vivo, we could expect to see different phenotypes in heterozygous mice carrying single patient *Tubb4b* mutations versus null mutations (i.e. haploinsufficiency). We therefore used CRISPR-Cas9 mediated genome editing to engineer into mice the *Tubb4b* patient variants carried in PCD-only (p.P259L, p.P259S), syndromic PCD+SND (p.P358S) and SND-only (p.R391H) patients, as well as deletion alleles (Fig. 4A, Fig. S3A, Fig. S10A). While animals heterozygous for the two null *Tubb4b* alleles described above (Fig. 2, Fig. S3) exhibited normal neonatal survival and growth (Fig. S9D), with no reduction in airway cilia length (Fig. S9A,B) or fertility defects (Fig. S9C), founder mice carrying PCD-causing mutations exhibited increased postnatal lethality (Fig. 4B). They developed pronounced hydrocephaly neonatally (Fig. 4C,D, D’) and defects in mucociliary clearance within the upper airways (Fig. 4F,F’), with loss of multicilia throughout the respiratory epithelium (Fig. 4G,H). Moreover, we were unable to transmit any of the PCD variants because surviving founders exhibited both male and female infertility (Fig. 4E). These mice therefore phenocopy PCD patients.

In contrast, we were able to generate a mouse line carrying the SND-only *Tubb4b*^*R391H/+*^ variant (Fig. S10A,B), although males remained infertile due to defects in spermatogenesis (Fig. 4I). *Tubb4b*^*R391H/+*^ mice did not develop any retinal degeneration (Fig. S10C-E) even when aged (Fig. S10E). We observed a significant (20%) reduction in airway cilia length (Fig. 4J-L) in *Tubb4b*^*R391H/+*^ mice, indicating a dominant effect, as we could confirm TUBB4B protein levels were identical between control and p.R391H/+ littermates in vivo (Fig. 4M). However, SND TUBB4B mutations in mice do not recapitulate the phenotypes of human SND patients.

We further examined the effects of the disease variants on tubulin autoregulation in human airway nasal epithelial cultures by carrying out proteomic and transcriptomic analyses on lysates from healthy donors and patients carrying either the p.P259L or the p.P358S variant. Comparable levels of TUBB4B protein were detected between controls and patients (Fig. 4N), further suggesting haploinsufficiency is not the disease mechanism. However, bulk RNA sequencing (RNASeq) of the samples from these two patients and control donors revealed distinct molecular signatures. Only the p.P259L patient samples displayed a two-fold increase in *TUBB4B* mRNA and concomitant increase in mRNAs encoding *TUBA1A* and *TPPP3*, a microtubule polymerizing protein (Fig. 4O). This is consistent with the expectation that the variant disrupting α/β tubulin heterodimer assembly would also impact on the tight regulatory feedback in cells that ensures an appropriate balance of α and β subunits (28, 29). In keeping with this concept, we also observed upregulation of the mRNA encoding the *TBCA* and *TBCB* tubulin chaperones involved in binding and stabilizing the nascent β-tubulin and α-tubulin protein, respectively (Fig. 4P). These tubulin autoregulation signatures were not observed in the syndromic PCD+SND p.P358S samples, consistent with our observation that this variant does not disrupt tubulin heterodimer assembly (Fig. 3J) but rather exerts downstream dominant effects on microtubule dynamics (Fig. 3L).

Together these data suggested that these disease-causing variants are acting via non-loss-of-function mechanisms i.e. through dominant-negative or gain-of-function effects. Indeed, it is difficult to distinguish between these two possibilities (30), particularly for tubulins (31). Despite a decreased intrinsic propensity for the PCD-only variants to assemble into microtubules, transcriptional upregulation of *TUBB4B* itself and its chaperones still produce mutant TUBB4B protein. This production of mutant protein could have competitive dominant-negative effects over wild-type TUBB4B by competing for tubulin chaperones. In contrast, incorporation of the p.P358S variant appeared to have assembly-mediated dominant-negative effects over the wild-type TUBB4B-containing microtubule lattices. Here, the variant poisoned microtubule dynamic properties in a dose-dependent manner. Overall, these results support distinct dominant-negative modes of action of *TUBB4B* mutations in each ciliopathy subtype. In the case of mice carrying PCD-causing *Tubb4b* mutations, these models phenocopied many patient features, at both cellular and physiological levels, consistent with the mutant variants acting in a dominant manner to disrupt centriolar and ciliary microtubules.

## *TUBB4B* mutations differentially localized across tubulin surfaces according to clinical phenotype

**Figure 5:**
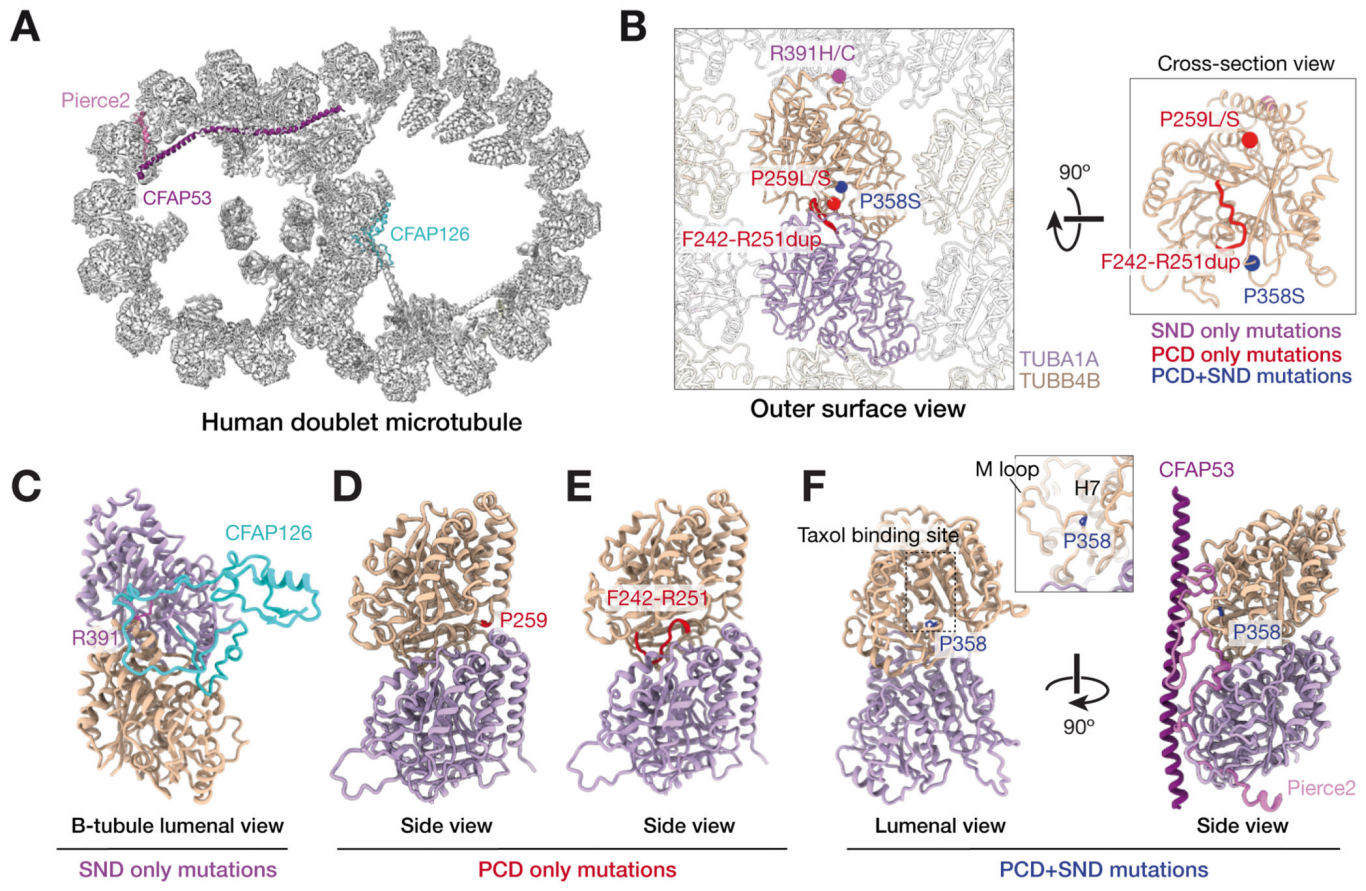
Structural environment of disease-causing variants of TUBB4B. (**A**) Human microtubule doublet cross-section (PDB ID: 7UNG) highlighting microtubule interacting proteins (MIPs) that interact with TUBB4B residues associated with disease. Microtubule doublets are the conserved cytoskeletal element of both primary and motile cilia, consisting of complete A-tubule with 13 protofilaments and incomplete B-tubules with 10 protofilaments. (**B**) Orthogonal views showing disease-causing TUBB4B variants within the ciliary microtubule doublet lattice. The human tubulin isotypes (TUBA1A (purple) and TUBB4B (gold)) were determined based on the human microtubule doublet cryo-EM density map (32) and abundance in human multiciliated respiratory cells by scRNAseq (64). Variant positions are indicated with spheres colored based on their disease association. Only one TUBB4B molecule is shown in the cross-section (right), where R391 is not visible. (**C**) Interaction of R391 of TUBB4B with the microtubule inner protein CFAP126. (**D-E**) p.P259 and loop p.F242-R251 locate at the intradimer interface. (**F**) p.P358 locates at the taxol binding site which interacts with multiple microtubule interacting proteins (MIPs) including, for example, PIERCE2.

Although TUBB4B is widely expressed, our mouse knockout studies indicated an essential and non-redundant function for this β-tubulin isotype in building airway cilia. In order to understand why, we undertook a structural approach, reprocessing cryo-electron microscopy (cryo-EM) data of the human microtubule doublet isolated from the axonemes of airway multiciliated cells (32) to determine a structure of the tubulin heterodimer to 2.8-Å resolution (Fig. 5A). Within this reconstruction, we could assign both α- and β-tubulin isotypes, based on their sidechain density. After evaluating each residue of the candidate β-tubulin isotypes, we determined TUBB4B to be the best fit to the density map and thus likely the predominant isotype incorporated into airway cilia axonemes in vivo (Fig. S11-S14). Thus, structural analysis further confirmed that TUBB4B is a cilia-specific tubulin, despite the expression of many other β-tubulin isotypes within this cell type.

The site of mutation in the TUBB4B variants associated with the three different phenotypic classes of disease (SND-only, PCD-only or PCD+SND) were differentially distributed across the structure of the protein, both within and between tubulin heterodimers (Fig. 5B, Table S3). The previously reported SND-only *TUBB4B* variants p.R391H and p.R391C (25) localized to the interface between adjacent tubulin heterodimers (Fig. 5C). The SND p.R391H/C mutations were moderately destabilizing to the protein but predicted to more strongly impact longitudinal interactions with the adjacent α-tubulin in neighboring heterodimers. Indeed, recent cryo-EM maps reveal an interaction between the α-tubulin C-terminal tail that links adjacent dimers to two conserved arginine residues (R391, R392) on β-tubulin to stabilize the microtubule filament (33). Moreover, several other pathogenic missense mutations have been reported in mostly neurodegenerative disorders at this position in other β-tubulin isotypes, including TUBB4A p.R391H/L, TUBB3 p.R391L, TUBB2A p.R391H and TUBB8 p.R391C, where these mutations were predicted to disrupt microtubule stability (31, 34).

The PCD-only group (p.P259L/S, p.F242_R251dup) variants localized to the intradimer interface, the interface between each α- and β-subunit of a tubulin heterodimer (Fig. 5D,E) and have not been reported to be associated with human disease. Both missense mutations at P259 were predicted to destabilize the protein itself but were more likely to affect the interface with α-tubulin (Table S3). A similar disruption of this intradimer binding interface by the p.F242_R251dup was expected, although the effects of an insertion mutation on protein stability are more challenging to predict.

The PCD+SND syndromic variant (p.P358S) was located within the intralumenal face of the tubulin heterodimer- the side facing into the microtubule lumen- close to binding site of the anti-tumor drug taxol (Fig. 5F). This site promotes lateral aggregation of taxol-bound protofilaments into stabilized microtubules (35). This intralumenal position is also known to interact with many microtubule inner proteins (MIPs) within cilia axonemes (36). The p.P358S mutation was predicted to be destabilizing and could also disrupt TUBB4B interactions at the intralumenal side of protofilaments, potentially with MIPs or lateral interactions between protofilaments. p.P358L/A/S mutations have also been reported in *TUBB8*, and are associated with female infertility (37).

Our combined analysis showed that different *TUBB4B* mutations disrupt distinct molecular surfaces of β-tubulin, which in turn disturb different aspects of tubulin function and result in different ciliopathic disease phenotypes. We propose that how these mutations impact tubulin heterodimers and their assembly into higher-order structures within cilia and centrioles dictates whether patients present with purely motile ciliopathy features, purely sensory ciliopathy features or a syndromic form affecting both cilia types.

## TUBB4B is an organelle-specific isotype localized to centrioles and cilia

While our structural analysis confirmed TUBB4B to be the predominant β-tubulin isotype in motile cilia axonemes, it remained unclear whether TUBB4B also contributes more broadly to other microtubules in cells where it is expressed or whether organelle-specific microtubule lattices exist.

**Figure 6:**
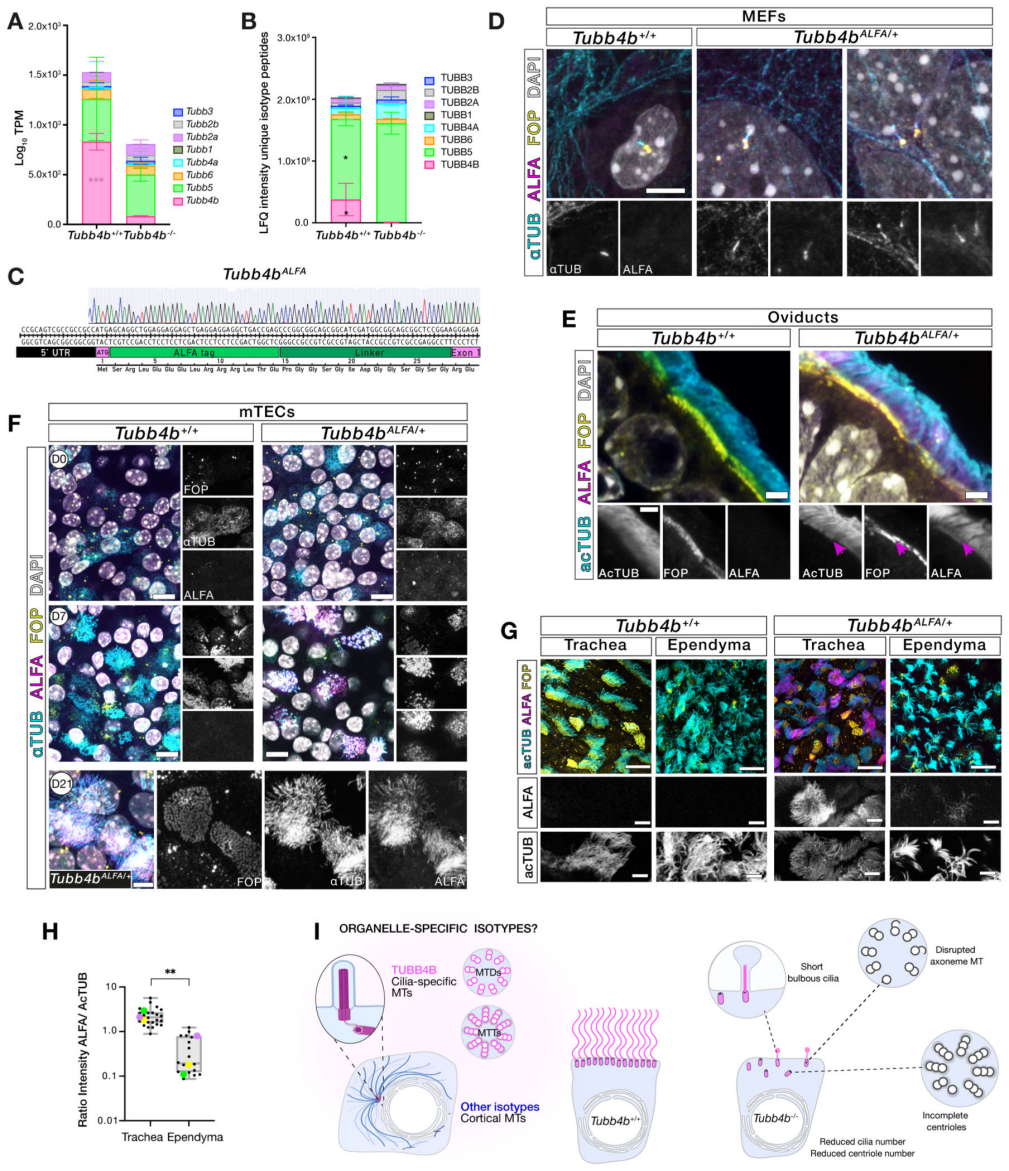
TUBB4B was a centriole and cilia-specific isotype. (**A**) Transcriptomic data from neonatal *Tubb4b* tracheas of β-tubulin isotype mRNA expression profile. (**B**) Total proteomic quantitation of β-tubulin isotype unique LFQ peptides from neonatal *Tubb4b* tracheas. (**C**) Schematic of ALFA-tagging of endogenous *Tubb4b* locus to generate *Tubb4b*^*ALFA*^ mice. (**D**) Immunofluorescence of serum-starved primary *Tubb4b*^*+/+*^ and *Tubb4b*^*ALFA/+*^ MEFs stained for centrioles (FOP, yellow), TUBB4B (ALFA, magenta) and pan-α tubulin (αTUB, cyan). (**E**) Immunofluorescence of P57 *Tubb4b*^*+/+*^ and *Tubb4b*^*ALFA/+*^ oviduct sections stained for centrioles (FOP, yellow), TUBB4B (ALFA, magenta) and acetylated-α tubulin (AcTUB, cyan). Centriolar TUBB4B staining (magenta arrow). (**F**) Wholemount immunofluorescence of *Tubb4b*^*+/+*^ and *Tubb4b*^*ALFA/+*^ mTEC cultures stained for centrioles (FOP, yellow), TUBB4B (ALFA, magenta) and pan-α tubulin (αTUB, cyan), at day 0 (D0), 7 (D7) or 21 (D21) post-airlift. (**G,H**) Comparative quantitation of endogenous TUBB4B in cilia by wholemount immunofluorescence of P8 *Tubb4b*^*+/+*^ (N= 2) and *Tubb4b*^*ALFA/+*^ (N=3) matched trachea and ependyma stained for centrioles (FOP, yellow), TUBB4B (ALFA, magenta) and acetylated-α tubulin (AcTUB, cyan), acquired with identical setting. (**H**) Relative intensity ratio of ALFA:AcTUB in cilia with mean from each animal (color matched). This revealed significantly higher levels of TUBB4B in tracheal cilia, supporting ependymal cilia contained alternate isotypes. (**I**) Summary model for a cilia- and centriole-specific role for TUBB4B. Scale bars: 5 μm (D), 2 μm (E), 10 μm (F,G merged), and 5 μm (F lower row, G single channel). (**A, B**) Stacked graphs: mean ± SD from N=3-5 biological replicates. (**H**) Box and whisker plot: median ± upper and lower extremes of all samples, where colored dots represent mean of each biological replicate from N=3, n=17-23 cells. Student’s t-test: *, p ≤ 0.05; **, p ≤ 0.01; ***, p ≤ 0.005.

To rule out a general tubulin deficiency in *Tubb4b* mutants, we examined transcriptomic and proteomic expression of all β-tubulin isotypes in neonatal tracheas (Fig. 6A,B). Increased protein levels of the highly similar TUBB5 and TUBB4A isotypes in *Tubb4b*^*-/-*^ meant that overall β-tubulin levels were not significantly changed. This suggests that phenotypes were due to lack of function of a specific, and non-redundant, isotype required for building cilia.

To determine TUBB4B localization in vivo, we generated an endogenously tagged *Tubb4b*^*ALFA*^ mouse line using the 13 amino acid ALFA epitope that enabled the use of high specificity and sensitivity nanobody reagents (Fig. 6C). In primary *Tubb4b*^*ALFA/+*^ mouse embryonic fibroblasts (MEFs), low levels of TUBB4B were specifically enriched in centrioles and cilia axonemes (Fig. 6D). In multiciliated cells, such as the oviduct (Fig. 6E), highly expressed TUBB4B localized to centrioles (FOP-positive, magenta arrowhead) and axonemes. We next examined TUBB4B localization during centriole biogenesis in mouse tracheal epithelial cell (mTEC) differentiation. We observed specific low-level localization in parental centrioles but not in cytoplasmic microtubules (D0, Fig. 6F), early in differentiation. During centriole amplification (D7, Fig. 6F), localized TUBB4B signal was observed in amplifying centrioles and subsequent elongating axonemes (D21, Fig. 6F).

We hypothesized that the phenotypic sensitivity of a given tissue (and cilia type) to TUBB4B loss would reflect the relative ratios of TUBB4B to other isotypes locally available. To test this, we isolated neonatal trachea (affected by TUBB4B loss) and ependyma (not affected by TUBB4B loss) from *Tubb4b*^*+/+*^ and *Tubb4b*^*ALFA/+*^ littermates and performed quantitative immunofluorescence (Fig. 6G,H). We observed a ten-fold difference in the axonemal content of TUBB4B between these motile cilia types.

Together, these results demonstrate that although TUBB4B is expressed in multiple tissues and preferentially localized to centrioles and cilia, cilia in different cell types are composed of different ratios of β-tubulin isotypes and thus differentially sensitive to TUBB4B loss. This supported a model where, in certain tissues where TUBB4B inherently is not highly represented in cilia, such as MEFs or ependyma, other tubulin isotypes can compensate in the absence of TUBB4B. However, these tissues could still be impacted by variants that integrate into microtubules to exert a dominant-negative effect, such as the syndromic PCD variant p.P358S (Fig. S15), which abrogates microtubule dynamics.

## Discussion

Given that cilia are by definition microtubule-based organelles, it is perhaps surprising that mutations in tubulin genes have not been observed previously to be associated with ciliopathies. This is likely due to a high level of redundancy amongst tubulin isotypes capable of building cilia. However, our human disease and mouse genetic data now uncover a specific requirement for TUBB4B in the construction and function of ciliary axonemes in specific tissues. We found that disease-causing TUBB4B variants can act in a dominant manner to cause a spectrum of ciliopathic diseases. The locations of the mutations of these variants across the β-tubulin protein result in different effects on tubulin heterodimer assembly and polymerization into higher-order structures of microtubule doublets and triplets, ultimately impacting organelle number and axoneme size. These findings explained the different disease presentations across patients carrying different variants.

Differences in the patterns and levels of tubulin isotype expression, including *TUBB4B*, as well as tissue-specific differences in the ratio of different tubulin isoforms used to build cilia, likely explain why certain specialized cilia and tissues are more sensitive to *TUBB4B* mutations (Fig. S15). In some axoneme types, like those of microtubule doublets of respiratory cilia (38), we demonstrated that only one predominant β-tubulin isotype is utilized. In these cilia, mutations that inhibited heterodimer assembly completely disrupted centriole biogenesis and axoneme elongation as other isotypes cannot compensate, thus explaining why PCD-only phenotypes were observed. In other tissues, where a ‘mix’ of tubulin isotypes may be used to build different ciliary axonemes, other isotypes could compensate for the inefficient integration of *TUBB4B* variants in PCD-only patients (or in KO mice) into these structures, and therefore, cilia function was not compromised. Hence, heterodimer-impaired TUBB4B mutations resulted in PCD-only phenotypes. In contrast, the syndromic PCD+SND variant (p.P358S) could robustly integrate into axonemes and acted in a dominant-negative manner to disrupt the microtubule lattice, thereby leading to additional sensory and renal disease phenotypes in any tissues where TUBB4B was highly expressed. In the polymerization-impaired TUBB4B variants found in SND-only patients (p.R391H/C), we observed less dramatic effects on cilia length in vivo and in vitro. This is consistent with more subtle structural defects in the kinetics or stability of axonemal microtubules into which such variants integrated, and a tissue-specific sensitivity to dysfunction that leads to sensory ciliopathy features.

Our data also suggest that in some contexts, it is possible that different tubulin isotypes are able to compensate for bespoke properties needed to withstand the high mechanical demands of cilia motility and the high structural order of microtubule doublets. For example, centriole amplification and ciliogenesis in mouse ependymal cells appear to be unimpacted by TUBB4B loss, unlike ciliary airway cells, although ependymal cells expressed seemingly similar patterns of isotypes and levels of *Tubb4b* mRNA to ciliated airway cells (Fig. S4J). However, visualization of specific tubulin isotypes in microtubule networks across time and cellular space in vivo through endogenous tagging of tubulin genes using *Tubb4b*^*ALFA*^ showed that wild-type ependymal cilia contain 10x less TUBB4B protein than tracheal cilia. Thus, alternate isotypes such as the highly similar TUBB4A may compensate for the loss of TUBB4B function (39). These observations are consistent with *Drosophila* studies suggesting that only isotypes with a particular amino acid sequence in the carboxyl terminus (EGEFXXX) are required for normal axonemal function (7, 40–42). This motif is only found in TUBB4A and 4B in mammals and is the site of post-translational modifications associated with cilia stability. These findings raise the possibility that mammalian TUBB4A/B is a motile cilia-specific β-tubulin required for the unique mechanical and structural properties of motile axonemes (43).

Cilia on the choroid plexus are dramatically remodelled during development from motile to sensory/immotile (44) and then lost gradually with age (45). Our work suggests that defects in choroid plexus function could underlie hydrocephaly phenotypes more broadly in PCD patients, rather than defects in ependymal cells, which have been largely accepted to be the culprit. The exact function of what choroid plexus cilia do remains unclear but it has been suggested choroid plexus cilia regulate fluid transcytosis and their motility could help cilia sample CSF (46). In a rapidly growing body of evidence for non-genetic causes of hydrocephaly, the importance of the choroid plexus in triggering innate immune and CSF secretory responses to drive hydrocephaly has been linked to insult-induced cilia loss in choroid plexus epithelial cells (47, 48). Moreover, in PCD patients with RGMC phenotypes, like *Multicilin* variants, MRI imaging revealed fully penetrant hydrocephaly with choroid plexus hyperplasia (49). Future studies will be required to understand the mechanisms by which cilia loss regulates CSF secretion and homeostasis in the choroid plexus.

Our study raises an intriguing question of how a cell expressing different tubulin isotypes preferentially creates specific isotype-enriched microtubule structures with different proportions of available isotypes. One possibility is regulation by the large class of microtubule-associated proteins, which can interact with tubulin and microtubules to affect their dynamic and physical properties (50). To dissect this would require our in vivo approaches, which preserve both the endogenous network of regulatory factors and tubulin balance. Our endogenously tagged *Tubb4b*^*ALFA*^ model allows us to monitor in development and disease isotype-specific functions sensitively during the organization of different cellular microtubule arrays. Such approaches are necessary to understand the molecular mechanisms leading to isotype-specific differences in the intracellular microtubule networks which support bespoke cell functions. For example, given the pleiotropic features of PCD+SND p.P358S patients, which include effects on kidney function, heart and bone growth, it will be important to use our *Tubb4b*^*ALFA*^ model to study contributions of TUBB4B to not only primary cilia within these tissues, but more widely to highly specialized cardiomyocytes and renal tubular epithelia that each have distinct cytoskeletal networks. Indeed, a single individual has been identified carrying a de novo p.Q11R variant in *TUBB4B* without clear PCD and exhibiting instead sensorineural hearing loss but not LCA, renal Fanconi Syndrome and hypophasphatemic rickets (51).The Q11 residue is close to the tubulin catalytic GTPase site and is proposed to lead to hyperstabilized microtubules.

In conclusion, our study provides detailed mechanistic insights into how *TUBB4B* variants cause a spectrum of ciliopathic diseases that spans both sensory and motile ciliopathies. The disease presentation manifesting in patients depends on how each variant affects tubulin heterodimer pools, as well as the differential tubulin isotype composition of the cilia and centrioles into which they are incorporated. Our study extends the understanding of tubulinopathies outside of classical neurological features, links them with ciliopathies, and suggests how tubulin diversity in humans underlies and facilitates the diversity of cilia seen in vivo.

## Materials and Methods

### Subjects

Twelve affected individuals from twelve unrelated families and their healthy relatives were included in the study (6 females and 6 males). Genomic DNA was extracted from peripheral blood by standard procedures. Signed and informed consent was obtained from the affected individual as well as relatives through approved protocols. For P1 (UOE), the study was approved by the London-West London & Gene Therapy Advisory Committee Research Ethics Committee (REC number 11/LO/0883), P2 by London-Bloomsbury Research Ethics Committee (REC 08/H0713/82; IRAS ID 103488), P3 by the Institutional Ethics Review Board of the University Muenster (2015-104-f-S), and P4 and P5 by the Institutional Review Board from Institut national de la santé et de la recherche médicale (IRB00003888 - approval n°15-259. Protocols for UNC (P6, P7) human studies were approved by the Institutional Review Board at the University of North Carolina and were performed in compliance with ethical regulations. P8 was recruited for WGS as part of the 100,000 Genomes Project, under approved Research Registry Project RR185 'Study of cilia and ciliopathy genes across the 100,000 GP cohort'. For P9, the study protocols were approved by the Institutional Review Board at Washington University in St. Louis. P10 was recruited under approved studies approved by the Institutional Review Board for Human Use at the University of Alabama at Birmingham (US), in compliance with ethical regulations. P11 was recruited under the Undiagnosed Disease Network protocol 15-HG-0130 approved by the National Institutes of Health Institutional Review Board. For P12, the study protocols were approved by the Pediatric Ethics Committee of Tuscany.

### Whole genome sequencing (WGS) and candidate prioritization

For P1 (UOE), DNA was sequenced by WGS at Edinburgh Genomics (21). Libraries were prepared using the Illumina TruSeq PCR-free protocol and sequenced on the Illumina HiSeq X platform. The average yield per sample was 136 Gb, with mean coverage of 36x (range 33.9-38.3). After first running analysis with a virtual gene panel of 146 genes, based on the PCD PanelApp panel (v1.14)(52) with five additional genes identified in the literature (*CFAP300, DNAH6, DNAJB13, STK36* and *TTC25*), no diagnostic variants were identified in P1. Expanded analysis identified a *de novo* missense mutation p.P259L (chr9:g.137242994:C>T (hg38)) in the gene *TUBB4B* only in the patient, and not present in either parent (Fig. S1A).

*TUBB4B* is an outlier in gnomAD in terms of constraint, especially in the overrepresented synonymous category (Z-score bottom 1.4% of all genes, 1.5x observed vs expected variants). The gene is also highly intolerant to missense variants, (Z-score top 0.4% of all genes, 0.28x observed vs expected variants) and intolerant to loss of function variants (probability loss of function intolerant (pLI) 1, 0.2x observed vs expected variants). Gene annotation for *TUBB4B* was obtained from gnomAD (v2.1.1, (53) https://gnomad.broadinstitute.org/gene/ENSG00000188229?dataset=gnomad_r2_1) and ExAC (v0.3, (54)). The constraints table was downloaded from the gnomAD website (https://gnomad.broadinstitute.org/). Variant annotation was obtained from the Ensembl Variant Effect Predictor (v99, (55)).

We examined the sequence context of P259 residue given it arose repeatedly by independent mutation in 7/12 patients within this study’s cohort.

GTCCCGTTT

-V--P--F-

The change of P>L is CCG>CTG, so deamination of a methylated CpG seems the most likely cause of that mutation. The other change at this residue P>S is CCG>TCG, which could be caused by non-canonical methylation MCG followed by deamination (56). In contrast, the sequence context of P358 which also arose as an independent *de novo* mutation in 4/12 patients is:

CCACCTCGG

-P--P--R-

The change of P>S is CCT>TCT, which is unlikely to be caused by non-canonical methylation.

For P8, data was accessed, analysed and filtered as described in (57). Data was reviewed by the Airlock Committee prior to export.

### Whole exome sequencing (WES) and NGS targeted panel

Details of how WES genomic libraries for P2-P7 and P8-P12 were generated, captured and sequenced are summarized in Table S2.

### Ciliary nasal brushing and high speed videomicroscopy

Biopsies and brushing of ciliated epithelium were obtained using a cytology brush from nasal mucosa (inferior turbinate) of the affected individuals P1-P6, P8-P10, P12 and p.R391C and processed for ciliary investigations. All clinical experiments were performed in the absence of acute respiratory tract infections. Cilia beat frequency and pattern were assessed by high-speed video microscopy at a frame rate of >500 frames per second. Video microscopy of ciliated epithelial cells was performed using an inverted microscope with a 20X phase contrast objective (Eclipse Ti-U; Nikon, Melville, NY) enclosed in a customized environmental chamber maintained at 37 °C. Images were captured by a high-speed video camera and processed with the Sisson-Ammons Video Analysis system (Ammons Engineering, Mt. Morris, MI, USA) and analyzed using established methodologies (58). Cilia beat frequency was analyzed in at least 4 fields obtained from each cell preparation. Cells were collected under approval from appropriate local authorities including Washington University institutional Review Board (IRB) approval and the local ethics committee DC-2008-512, Paris-Necker.

### Transmission electron microscopy- human

Airway biopsies were immersed in 2.5% glutaraldehyde and processed by standard procedures for transmission electron microscopy ultrastructural analysis (59). Ultrathin sections were examined at a final magnification of 60000x without knowledge of the clinical data. In samples without a ciliary agenesis phenotype, analysis of at least 50 transverse ciliary sections of different cells were required to study the internal axonemal structure according to a quantitative method (60). Ciliary ultrastructure results were expressed as a percentage of abnormal cilia among the total number of cilia analysed. As previously reported, up to 10% of cilia in control specimens can exhibit ultrastructural defects (61). For each ciliary study, axonemal abnormalities were expressed as the concerned ultrastructure (*i.e*. dynein arms, central complex and/or peripheral microtubules). In the case of ciliary agenesis, multiple ultrathin sections are analyzed but very few ciliary cross-sections are observed and analyzed (*e.g*. P4 had 9 cilia cross sections, and P5 had 4 cilia cross-sections, both showed outer doublet defects in cross-sections). As such, quantification of these parameters is not feasible and is skewed by the fact the primary defect is cilia rarefaction.

ET data of a section (300 nm thick, stained plastic-embedded) was collected using a TEM CM10 (Philips, Amsterdam, The Netherlands) equipped with a TemCam-F416 camera (TVIPS, Gauting, Germany). The microscope was operated at an acceleration voltage of 80 kV. The tilt series was collected manually from -45° to 35° at 2.5° intervals (single-tilt axis) with a final pixel size of 1,102 nm. The tilt series were aligned and reconstructed using IMOD (62).

### Air liquid interface culture- human

Primary human nasal epithelial cells were expanded at 37 °C in media selective for basal cells (PneumaCult™- Ex Plus Medium Stemcell™ Technologies, Cambridge, UK) or specialized media (58). At 80% confluent basal cells were dissociated and seeded into 6 mm transwell inserts (Corning^®^ Transwell^®^ polyester membrane cell culture inserts, Flintshire, UK). Once a confluent base monolayer had formed, the apical fluid was removed and the basolateral fluid was replaced with ALI media (PneumaCult™-ALI Medium, Stemcell™ Technologies, Cambridge, UK) to promote differentiation. Experiments were performed once cells had been at ALI for at least 3 weeks and were fully differentiated into ciliated epithelium. For RNA and proteomic studies, transwell insert membranes with cells were cut out and stored in RNAlater (ThermoFisher) or snap frozen respectively and stored at -80 °C until use. Cell preparations were maintained in culture for four to twelve weeks.

### Human proteomics studies- in gel digestion

Frozen cell pellets of cultured primary nasal epithelia from healthy unrelated controls or unaffected parent with parallel cultures from patient samples (p.P259L (P1), p.P358S (P9)) were lysed in 2% SDS in PBS and resolved by SDS-PAGE. Each insert was treated as an experimental replicate and graphed separately. Aiming to enrich for tubulin peptides, gel sections (between molecular weight markers of 37-75 kDa) were cut out and further dissected into 1 x 1 mm^2^ fragments. These were dehydrated with acetonitrile (ACN), reduced with 10 mM DTT and 50 mM ammonium bicarbonate (AB) for 20 min at 56 °C, followed by alkylation with 55 mM iodacetamide and 50 mM AB for 1 h RT. Samples were washed by sequential dehydration/hydration steps alternating between ACN and 50 mM AB. Then, samples were digested with trypsin (Promega) at 37 °C for overnight, extracted with 0.1% trifluoroacetic acid (TFA) and 80% ACN 0.1 % TFA. The combined eluates were concentrated in a CentriVap Concentrator (Labconco) and loaded onto StageTips. The tryptic peptides eluted from StageTips (80% ACN, 0.1% TFA) were lyophilised and resuspended in 0.1% TFA. Samples were analysed on a Q Exactive plus mass spectrometer connected to an Ultimate Ultra3000 chromatography system (Thermo Scientific, Germany) incorporating an autosampler. 5 μL of each tryptic peptide sample was loaded on an Aurora column (IonOptiks, Australia, 250 mm length), and separated by an increasing ACN gradient, using a 40 min reverse-phase gradient (from 3%–40% ACN) at a flow rate of 400 nL/min. The mass spectrometer was operated in positive ion mode with a capillary temperature of 275 °C, with a potential of 1,500 V applied to the column. Data were acquired with the mass spectrometer operating in automatic data-dependent switching mode, using the following settings: MS 70k resolution in the Orbitrap, MS/MS 17k resolution obtained by HCD fragmentation (26 normalised collision energy). MaxQuant version 1.6. was used for mass spectra analysis and peptide identification via Andromeda search engine (63) using standard settings apart from: Match between runs was enabled. Trypsin or LysC was chosen as a protease with minimum peptide length 7 and maximum of two missed cleavage sites. Carbamidomethyl of cysteine was set as a fixed modification and methionine oxidation and protein N-terminal acetylation as variable modifications. Total proteomic data are available via ProteomeXchange with identifier PXD036304.

### Human transcriptomic studies

Frozen cell pellets of cultured primary nasal epithelia from healthy unrelated controls or unaffected parent with parallel cultures from patient samples (p.P259L (P1), p.P358S (P9)). Briefly, total RNA from each culture was purified using a miRNAeasy Micro Kit (1038703, Qiagen), cleaned up with MinElute (74204, Qiagen) and treated with Turbo DNAse free (AM1907, Invitrogen). RNA quality and integrity were assessed on the Agilent 2100 Electrophoresis Bioanalyser Instrument (#G2939AA, Agilent Technologies Inc) using RNA 6000 Nano or Pico (#5067-1511, 5067-1513), then quantified using Qubit 2.0 Fluorometer (#Q32866, Thermo Fisher Scientific Inc) with a Qubit RNA High Sensitivity assay kit (#Q32855). DNA contamination was quantified using the Qubit dsDNA HS assay kit (#Q32854). Poly-A mRNA was isolated with magnetic module (#E7490, New England Biolabs) from 20-100 ng of each total RNA sample. Libraries were generated with a NEBNEXT Ultra II Directional RNA Library Prep kit (#E7760, New England Biolabs) using random hexamers as primers. Sequencing was performed using the NextSeq 500/550 High-Output v2.5 (150 cycle) Kit (#20024907, Illumina) on the NextSeq 550 platform (#SY-415-1002, Illumina). Total RNASeq data has been deposited in GEO under accession code GSE214070 (https://www.ncbi.nlm.nih.gov/gds/?term=GSE214070).

### Nasal epithelial cell immunofluorescence

Nasal cells were fixed directly from the patient or after expansion in ALI cultures for wholemount. If direct, samples suspended in cell culture media were spread onto glass slides, air dried, and stored at –80 °C until use. Expanded cultures were fixed directly on the membrane in 4% PFA/PBS, then immunostained and imaged (Tables S7,S8). Nuclei were stained using 4′, 6-diamidino-2-phenylindole 1.5 µg/mL.

### Genetic and three-dimensional structure analysis

The 3-dimensional structure of wild-type TUBB4B and mutant variants was predicted using I-TASSER and based on *TUBB4B* NM_006088.5 reference. Predicted models were aligned on the cryo-electron microscopy structure of a GDP-protofilament (GDP-K MT, EMD-6353, PDB: 3JAS) using the UCSF Chimera software. We modelled the molecular effects of missense mutations using the stability predictor FoldX v5 with all default parameters and three replicates (Table S3). We used the PDB structure 5FNV, chain B, as it possesses both the inter- and intra-dimeric interfaces with α-tubulin. ΔΔG subunit values represent the predicted effects of mutations on the TUBB4B molecule alone, ignoring any intermolecular interactions, while the ΔΔG full values were calculated using the full protein complex structure, and thus include effects from the predicted disruption of α-tubulin interfaces. To visualize the structure of the Dup variant, we modelled the structure of the full variant protein using SWISS-MODEL.

### Identification of the tubulin isotypes that form axonemal microtubule doublets

The tubulin isotypes that form respiratory axonemal microtubule doublets were determined using sidechain density from the 3.6-Å resolution structure of human microtubule doublets (32). All potential isotypes were first determined by mass spectrometry of the sample used for cryo-EM analysis. Candidates for α-tubulin were TUBA1A, TUBA1B, TUBA1C, and TUBA4A. Candidates for β-tubulin were TUBB4B, TUBB2B, and TUBB5. Multiple sequence alignments were generated for the α- and β-tubulin isotypes to highlight positions in the primary sequence where the residues differed. The density corresponding to each site of variation was then examined to discriminate between candidate residues. For example, TUBB2B was excluded as an asparagine sidechain at position 57 that does not match the density (Fig. S12C). The methionine sidechain at position 293 and the alanine sidechain at position 365 of TUBB4B fitted better to the density than the valine (293) and valine (365) of TUBB5, respectively. After performing this sequence comparison at every variable residue, we determined that the amino acid sequence of TUBB4B was the best fit to the density. The same approach was used to identify the α-tubulin isotype as TUBA1A, where the glycine of TUBA1A at position 232 was a better fit to the density than the serine of TUBA1B, TUBA1C and TUBA4A. Our assignment is consistent with the abundance of tubulin isotypes in single-cell RNA-sequencing of human multiciliated respiratory cells (64).

### Site-directed mutagenesis

Patient-derived *TUBB4B* variants c.776C>T, p.P259L; c.775C>T, p.P259S; c.1072C>T, p.P358S; c.1171C>T, p.R391C; and c.1172G>A, p.R391H were generated by mutagenesis via inverse PCR with Phusion polymerase using vector pcDNA3.1-TUBB4B-C-(K)DYK (Genscript, Piscataway, USA) as a respective template with primers listed in Table S6. The amplified product was digested with DpnI to avoid religation of original non-mutated DNA. Constructs were amplified in XL1-Blue competent cells (Agilent, US), and the whole ORF of each plasmid was Sanger sequenced to confirm the presence of the patient mutation.

### Culture of RPE1 cells

hTERT- RPE1 cells (CRL-4000) were maintained at 37 °C, 5% CO_2_ in Opti-MEM Glutamax I medium, supplemented with 10% fetal bovine serum and 1% streptomycin/penicillin (Life Technologies, ThermoFisher Scientific). Cells were seeded at 2 x 10^5^ cells/well on glass coverslips in 12-well plates and maintained for 24 hours (80% confluence). Cells were transfected with wild-type or mutant FLAG-tagged TUBB4B plasmids (1 µg, pcDNA3.1-TUBB4B-C-(K)DYK constructs; Genscript) using the FuGene HD transfection reagent according to the manufacturer’s protocol (Promega).

### Microtubule co-localization and lattice dynamics in RPE1 cells

Microtubule dynamics were characterized 48 h post-transfection. Cells were directly fixed in ice-cold methanol (5 min at -20 °C) for the steady-state microtubule lattice or after having been maintained on ice for 20 and 30 min for microtubule depolymerization or for 30 min prior to incubation at 37 °C for microtubule repolymerization for 4 and 6 min., respectively. Fixed cells were permeabilized in PBS supplemented with 3% bovine serum albumin and 0.1% TritonX-100 (1 h RT) prior to immunostaining. Staining colocalization between positive FLAG and positive α-tubulin from a given cell area (ROI) was quantified using machine learning of Ilastik software(65), percentages of staining colocalization were generated using JACoP plugin on ImageJ software(66) and plotted using GraphPad software. Microtubule lengths were measured by determining the number of EB1 protein spots and the distance between the centrosome and each EB1 signal, in repolymerization state, using a Spot detector plugin within an ROI using Icy software (67). Individual distances were plotted using GraphPad software. Means of fiber alignment degrees (co-localization), EB1 spot numbers and microtubule lengths were calculated from two independent experiments (> 30 cells for each cell line). Statistical analyses were carried out by ANOVA and the PLSD Fisher test.

### Ciliary abundance and length in RPE1 cells

Transfected cells were propagated for 24 h at 37 °C, 5% CO_2_ in serum-free Opti-MEM Glutamax I medium to promote ciliation. Cells were fixed in ice-cold methanol and immunostained. Mean numbers of ciliated cells and cilia lengths were calculated from two independent experiments (> 100 cells for each condition) using ImageJ (66). Statistical analyses were carried out by the PLSD Fisher test according to the significance of the Student’s t test.

### IMCD3 TUBB4B stability and interaction studies

Human *TUBB4B* (NM_001372.3) constructs (wild type, p.P259L, p.P259S, p.P358S and Phe242_Arg251dup) were ordered from GeneWiz, Germany with a C-terminal ALFA tag using the sequence 5’ –AGCAGGCTGGAGGAGGAGCTGAGGAGGAGGCTGACCGAGTAG-3’. A single proline linker 5’-CCC-3’ was included between the cDNA and tag. Plasmids were sub-cloned into the pCDH-CMV-MSC-EF1α-Hygro plasmid (CD515B-1; Systems Biosciences, USA), using Nhe1 and Not1 restriction enzymes. Lentiviruses with a VSV-G pseudotype were produced (Viral Vectors Core, Shared University Research Facilities, University of Edinburgh, UK). To generate stable IMCD3 cell lines expressing control or patient variants, IMCD3 cells (CRL-2123, ATCC) were transduced with 3 x 10^7 copies/mL of virus with polybrene (H9268, Sigma) final concentration of 10 μg/mL in DMEM-F12 (12634010, Gibco), 10% FCS, 1% P/S to 80% confluency. After 24 hours, fresh media was added. After 96 hours following transduction, hygromycin was added to the media at a final concentration of 100 μg/mL and cells were selected for 7 days.

To test the ability of TUBB4B variants to heterodimerize, IMCD3 stable cell lines expressing wild type and patient variants fused in frame with the small C-terminal tag ALFA (NanoTag Biotechnologies) were grown to 80% confluency. Plates were placed on ice for 30 min to depolymerize microtubules after which the culture media was aspirated and cells scraped into 400 µl BRB80 buffer (80 mM PIPES, 1 mM MgCl_2_, 1 mM EGTA, pH 6.8) plus 10% glycerol, 0.2% Triton X-100, 5 µg /ml DNase I, Halt Protease inhibitor (Pierce) and 1 mM GTP (R1461, Thermo Scientific). Cells were lysed in a water bath sonicator for 10 min, incubated at 37 °C for 20 min and centrifuged at 13K rpm. Cleared supernatants were used to determine total protein levels or incubated with 20 µl ALFA SelectorPE beads (N1510, NanoTag Biotechnologies, Germany) for affinity capture of TUBB4B-ALFA for 1 hour at RT, washed x 4 times with BRB80 buffer and 10% Glycerol. Bound proteins were released by competition with 0.1 mg of ALFA-elution peptide (N1520, NanoTag Biotechnologies, Germany) for 15 min RT. Resin eluted or total lysates were resolved in acrylamide gels and transferred using Trans-Blot^®^ Turbo™ Transfer System (170-4150, Biorad) with transfer reagents Trans-Blot Turbo™ (Biorad 170-4270), then followed by iBind™ (SLF1000, Thermo Scientific) and iBind™ Flex Solutions (SLF2020, Thermo Scientific). Blots were immunoblotted using antibodies listed in Tables S7,S8 and imaged following incubation with chemiluminescent substrate SuperSignal™ West Pico Plus (34580, Thermo Scientific) on the ImageQuant 800 (Amersham) using either auto-exposure or manual with indicator of saturation. All quantifications done in non-saturated bands using ImageJ.

### In vitro transcription and translation studies

Plasmids (pCDH-CMV-MSC-EF1α-Hygro) containing the human *TUBB4B* (NM_001372.3) wild type and variants (p.P259L, p.P259S, p.P358S and Phe242_Arg251dup) with a C-terminal ALFA tag were used for TNT expression with the TnT ^®^ T7 Quick Coupled Transcription/Translation System (L1170, Promega), according to the manufacturer’s protocol. Reactions were loaded into NuPAGE precast gels, transferred onto PVDF membrane (1704272, BioRad), and then rinsed in water then TBST, and then blocked in 4% BSA in TBS plus 0.1% Tween. Membranes were then incubated overnight 4 °C with ALFA-HRP (N1502-HRP, NanoTag Biotechnologies, Germany) in 5% milk TBST then washed 3 X 10 min TBST and developed using Pierce SuperSignal Pico Plus (Pierce) reagent and imaged on ImageQuant.

To visualize tubulin heterodimer chaperone complexes the IVT reactions were run at different amounts of time. When adding excess tubulin for the tubulin ‘pulse’, 1 µl porcine tubulin extracted from brains was added after 80 min, and the reaction allowed to proceed for a further 60 min (1 µM). The reactions were loaded onto Invitrogen NativePAGE 4 to 16%, Bis-Tris, 1.0 mm, Mini Protein Gels (ThermoFisher, #BN1002BOX) according to the manufacturer’s instructions except blue dye was excluded and the gels were transferred onto nitrocellulose membranes and processed as above for subsequent immunoblotting.

### In vitro studies: Cloning, protein expression and purification

Expression and purification of affinity tag-free wildtype and mutant recombinant human TUBB4B/TUBA1A heterodimers as unlabelled, biotinylated and fluorophore-labelled proteins were overexpressed and purified following the protocol described previously (68).

### Polymerization of microtubules

GMPCPP-stabilized wildtype and mutant TUBB4B/TUBA1A microtubules were generated by polymerizing 1 mg/mL or 4 mg/mL unlabelled tubulin supplemented with 2.8% fluorescent tubulin and 2.8% biotinylated tubulin following the protocol described previously (68). Taxol stabilized mutant TUBB4B/TUBA1A microtubules were generated by polymerizing 3 mg/mL unlabelled tubulin supplemented with 2.8% fluorescent tubulin and 2.8% biotinylated tubulin following the protocol described previously (68). GMPCPP and taxol double-stabilized mutant TUBB4B/TUBA1A microtubules were generated following the method described earlier but with a slight modification where 1 mM GMPCPP was used instead of GTP.

### Microtubule dynamics

Free tubulin was prepared by supplementing unlabelled free tubulin with 3% fluorescent tubulin in 1xBRB80 containing 5% (w/v) glycerol, 2 mM GTP, 1 mM tris(2-carboxyethyl)phosphine (TCEP). The samples were incubated on ice for 5 min before centrifuging at 90,000 rpm at 4 ˚C for 10 min (Beckman Coulter, TLA 120.1) to remove aggregated tubulin. Final concentration was determined by using Bradford assay. The flow chambers were assembled as described previously (68). Next, the flow chambers were treated sequentially with 0.2 mg/mL neutravidin for 5 min, followed by 1% (w/v) pluronic for 15 min and lastly 0.2 mg/mL κ-casein for 5 min with two washes of 10 μL working buffer in between each treatment. The microtubule seeds were immobilized by perfusing 0.8 μL of resuspended polymerized microtubules into the chamber and incubated for 8 min before washing twice with 10 µL working buffer (1X BRB80, 5% sucrose, 1 mM MgCl_2_, and 1 mM TCEP). After checking the density of immobilized seeds in the chamber, the chamber was perfused with 10 µL of reaction mix containing oxygen scavenger mix (4.5 mg/mL glucose, 200 mg/mL glucose oxidase, 35 mg/mL catalase, and 2 mM GTP) and free tubulin (3.5 mg/mL). The flow chamber was sealed with Valap sealant (a 1:1:1 mixture of Vaseline, lanolin, and paraffin) and incubated at 37 ˚C for 5min before imaging. Three independent experiments were performed for varying ratios of wildtype TUBB4B/TUBA1A to mutant p.P358S (100:0, 75:25, 50:50). Time-lapse images were captured at 5 sec per frame for 15 min using iLAS3 ring-total internal reflection fluorescence (TIRF) microscope (Inverted: Nikon Ti2-E) with a Photometrics Prime95B camera. The microscope stage was kept at 37 ˚C using a warm stage controller. Excitation laser 561 nm was used.

### Dynamics assay analysis

In the analysis of TIRF images, the Mosaic plugin (https://mosaic.mpi-cbg.de/Downloads/update/Fiji/MosaicToolsuite/) of Fiji was used to remove the background prior to drawing kymographs (69). Lines were manually drawn along the vertical distance of the event for catastrophe time, while the catastrophe length was determined by manually drawing horizontal lines along the event before undergoing catastrophe. The pause time was determined by visually identifying the segments where no discernible growth was observed during the event. To estimate the time taken for a new microtubule to nucleate, the nucleation time was calculated by subtracting the catastrophe time from the total duration of the image series captured.

Since the polymerization rate did not allow for distinguishing polarity, all analyses were conducted by pooling events from both ends. The polymerization rate was determined by calculating the slope of the event (catastrophe length/catastrophe time). Catastrophe frequency referred to the total number of events divided by the total catastrophe time, while the nucleation frequency was defined as the total number of events divided by the total nucleation time. The pause fraction is a measure of the time during which a filament experienced a pause event, expressed as a fraction of the total catastrophe time for that filament. Due to the stochastic nature of catastrophe and nucleation, a Poisson distribution was assumed for catastrophe frequency and nucleation frequency. The standard error of mean catastrophe frequency was calculated by dividing the observed catastrophe frequency by the square root of the total number of events observed. Nucleation frequency was analyzed in the same manner.

Data presented represent 3 independent experiments, for a total analysis of 41 microtubule filaments WT:p.P358S (75:25) and 41 filaments wildtype (100:0) TUBB4B/TUBA1A.

### Cryo-EM data processing

The starting point for cryo-EM analysis was a stack of 208,558 particles used previously to calculate a structure of the 48-nm repeat of microtubule doublets isolated from human respiratory cilia (32). These particles were extracted in 512-pixel boxes from micrographs collected on a Titan Krios microscope (Thermo Fisher Scientific) equipped with a BioQuantum K3 imaging filter (slit width, 25 eV) and a K3 detector (Gatan) (Table S4). The micrographs had a defocus range of −0.8 to −2.0 μm and a pixel size of 1.37 Å. Each particle had undergone at least one round of contrast transfer function refinement (CTFRefine) and Bayesian polishing in RELION-4.0 (70) and was a survivor from multiple rounds of three-dimensional classification. In the previous publication (32) this particle set was used to calculate a composite cryo-EM map of the human microtubule doublets with a nominal resolution of 3.6 Å (EMD-26624). Full details of the sample preparation, microscope settings, and data processing steps are provided in (32).

In this study, we have used this particle set to improve the resolution of a α/β-tubulin heterodimer to be able to confidently identify the α- and β-tubulin isotypes. Using RELION-4.1 (70), we created a soft-edged mask over one of the best resolved regions of the map – the microtubule wall (the ribbon) that partitions the lumens of the A and B tubule (Fig. S11A). Density outside the masked region was subtracted to focus subsequent refinements on the ribbon microtubule density. Two rounds of focused local refinement with decreasing initial angular sampling (0.3 and 0.1, respectively) with the subtracted ribbon density yielded a nominal resolution of 2.8 Å (Fig. S11A) based on the Fourier Shell Correlation=0.143 criterion, which is close to the Nyquist limit imposed by the 1.37 Å pixel size set during data collection. A subregion of this map, corresponding to α/β-tubulin and colored by local resolution (Fig. S11B), shows that the majority of the tubulin dimer is resolved to better than 3 Å, consistent with the nominal resolution of 2.8 Å. Maps were sharpened using standard postprocessing in RELION-4.1. The map has been deposited to the Electron Microscopy Data Bank with accession number EMD-40480.

### Identification of tubulin isotypes from cryo-EM maps

The tubulin isotypes that form human respiratory axonemal microtubule doublets were determined using well-resolved sidechain density from the 2.8-Å resolution map. All potential isotypes were considered with the exception of TUBAL3, which has an insertion of 7 residues at position 39 incompatible with the α-tubulin cryo-EM density. Multiple sequence alignments were generated for the α- and β-tubulin isotypes to identify positions in the primary sequence where the residues differed (Fig. S14). The density corresponding to each site of variation was then examined manually to discriminate between candidate residues. Candidates were excluded if their residues had sidechains that extended beyond the cryo-EM density or if their sidechains were smaller than indicated by the cryo-EM density. Using this approach TUBA1A and TUBB4B were identified as the best fit to the density for α- and β-tubulin, respectively (Fig. S12 and S13), although we cannot rule out other tubulin isotypes making a minor contribution that is averaged out during cryo-EM processing. This assignment is consistent with the upregulation of *TUBA1A* and *TUBB4B* transcripts in single-cell RNA-sequencing of human multiciliated respiratory cells (64).

### Model Refinement

To refine the TUBA1A and TUBB4B models, chains LI and LH and residues within 10 Å of these chains were extracted from PDB: 7UNG using ChimeraX and refined into the improved cryo-EM density using ISOLDE (71). ISOLDE’s command `write phenixRsrInputˋ was used to create a parameter file for subsequent refinement in Phenix.real_space_refinement (72). Refinement statistics are provided in Table S4. The model has been deposited to the Protein Data Bank with accession code 8SH7.

### Generation of mouse lines and patient mutation F0 founder mouse models

For the *Tubb4b*^*R391H/+*^ line, the NIH guide was followed for the care and use of laboratory animals, with approval from the French Ministry of Research (APAFiS # 20324) and following ethical principles in the LEAT Facility of Imagine Institute. For all other lines, animals were maintained in SPF environment and studies carried out in accordance with the guidance issued by the Medical Research Council in “Responsibility in the Use of Animals in Medical Research” (July 1993) and licensed by the Home Office under the Animals (Scientific Procedures) Act 1986 under project license number P18921CDE in facilities at the University of Edinburgh (PEL 60/6025).

*Tubb4b*^*R391H/+*^ mice were generated using CRISPR/Cas9 as described in Fig. S6, using guides detailed in Table S5 as shown in Fig. S10A. To generate *Tubb4b*^*ALFA/+*^ mice we used the guide and repair shown in Table S5. To generate *Tubb4b*^*+/-*^ mice (*Tubb4b*^*KO1/+*^ and *Tubb4b*^*KO2/+*^) we used CRISPR/Cas9 as described in Fig. S3A, using guides detailed in Table S5. To generate animals *Tubb4b*^*P259L/+*^, *Tubb4b*^*P259S/+*^ and *Tubb4b*^*P358S/+*^ mice, a similar targeting strategy was attempted using a variety of guide and repairs detailed in Table S5. Silent mutations were included to block the re-cutting as reported to increase the HDR accuracy and efficiency (73). However, we saw increased perinatal lethality and infertility in founders carrying patient-derived mutations and no lines could be established even using IVF techniques. Briefly, C57BL/6J female mice were super-ovulated and fertilized embryos were injected at the 1 cell stage. The microinjection mix consisting of RNPs with 0.35 µM of guides and 1.8 µM of recombinant GeneArt Platinum Cas9 (Thermo Fisher Scientific, US) and 20 ng/μl ssODN repair templates (Integrated DNA Technologies (IDT), US) was incubated at 37 °C for 10 min prior to pronuclear microinjection. Zygotes were cultured overnight before transfer to pseudopregnant CD1 females. Founders were genotyped by PCR and Sanger sequenced from genomic DNA, isolated from ear biopsies, using primers (see Table S6).

To establish colonies of *Tubb4b*^*R391H/+*^ and *Tubb4b*^*+/-*^ mice, founder mice were crossed with C57BL/6J and CD1 mice respectively to remove potential off-targets, and the heterozygous offspring then outcrossed to C57BL/6J and CD1 respectively at least 5 generations to maintain a colony. CD1 was used for *Tubb4b*^*+/-*^ mice to reduce the severity of neonatal lethality and hydrocephaly, coupled to small litter size, characteristic of C57BL/6J mice. Genotyping was performed using primers detailed in Table S6, followed by Sanger sequencing in-house or by Transnetyx (Cordova, TN). For basal body quantification (Fig. S3, S4) mice were crossed to transgenic line *Cen2GFP* (CB6-Tg(CAG-EGFP/CETN2)3-4Jgg/J, The Jackson Laboratory).

### Left-right patterning analysis of mouse models

For left-right patterning defect analysis, three E12.5 litters of *Tubb4b*^*+/-*^ intercrosses were dissected and organ laterality examined blind to genotype. All 27 of the embryos examined displayed situs solitus, including the six *Tubb4b*^*-/-*^ mutants.

### Electroretinographic analysis of mouse models

The function of the retina of ten *Tubb4b*^*391H/+*^ and ten wild-type C57BL/6J mice, aged of 2 months to 1 year, was analyzed by electroretinography using the CELERIS Next Generation Rodent ERG Testing platform, according to the manufacturer protocol (Diagnosys LLC, Cambridge, UK) and guidelines for animal safety. In brief, mice were dark-adapted overnight, anesthetized according to their weight and exposed to two four-step sequences of increasing light stimuli from 0.01 to 3 cd.s/m^2^ and a step sequence of 3 and 10 cd.s/m^2^, to elicit and record rod and cone-specific responses, respectively. Statistical analyses were carried out using GraphPad software using the post hoc Sidak’s test (two-way ANOVA).

### Mouse trachea proteomics

Tracheas from mutant and wild type littermate mice aged between P1 and P5 for *Tubb4b* KO experiments and between P40 and P100 for *Tubb4b*^*391His/+*^ experiments were used for total proteomics, using a filter aided sample preparation (FASP) method. Tracheas were flash frozen in liquid nitrogen and stored at -70 °C. Samples were lysed in 2% SDS, 0.1 M DTT, 0.1 M Tris-HCl pH 7.6 by pipetting, heating to 95 °C for 3 min and then removing non-solubilized material. The resulting sample was diluted to 100 μL with 0.1 M Tris-HCl pH 7.6. FASP purification and double digestion was done following the protocol described in (74) with following alterations: we used Vivaspin500 30k cut-off ultrafiltration devices (Sartorius) and 50 μL aliquots. All other steps, including washes, reduction and alkylation were identical. 1 μg of endoproteinase LysC (Wako) in 40 μL with 0.1 M Tris-HCl pH 8.5 was added. After overnight incubation at 37 °C, the LysC fraction was collected by centrifugation of the filter units for 20 min. The sample in the filter was then eluted with 40 μL of 0.1 M Tris-HCl pH 8.5 containing 1 μg of trypsin. Following a 4 h digestion, tryptic peptides were collected by centrifugation of the filter units for 20 min. Samples were acidified with 1% trifluoroacetic acid (TFA) and desalted using StageTips, dried using a CentriVap Concentrator (Labconco) and resuspended in 15 µl 0.1% TFA. Protein concentration was determined by absorption at 280 nm on a Nanodrop 1000, then 2 µg of de-salted peptides were loaded onto a 50 cm emitter packed with 1.9 µm ReproSil-Pur 200 C18-AQ (Dr Maisch, Germany) using a RSLC-nano uHPLC systems connected to a Fusion Lumos mass spectrometer (both Thermo, UK). Peptides were separated by a 140-min linear gradient from 5% to 30% acetonitrile, 0.5% acetic acid. The Lumos was operated using following settings: MS 120k resolution in the Orbitrap, MS/MS obtained by HCD fragmentation (30 normalized collision energy), read out in the ion-trap with “rapid” resolution with a cycle time of 1 s. The Limma package was used for mass spectra analysis and peptide identification (75). Total proteomic data are available via ProteomeXchange with identifier PXD036304.

For Fig. 2U, we specifically analyzed unique peptides for all α- and β-tubulins from the total proteomes from control mTECs across differentiation time points we previously generated (76). Briefly, here total mTEC proteomes were derived from two animals/genotype with three experimental replicates per time point (days 4–10, animal pair 1; days 14–18 animal pair two). The data were analyzed using the MaxQuant 1.6 software suite (https://www.maxquant.org/) by searching against the murine Uniprot database with the standard settings enabling LFQ determination and matching. The data were further analyzed using the Perseus software suite. LFQ values were normalized, 0-values were imputed using a normal distribution and standard settings.

### Mouse trachea transcriptomics

Tracheas from mutant, heterozygous and wild type P4 and P5 mice were dissected. RNA was extracted and measured as described for the human transcriptomics above, except turbo DNase was not used, the samples were instead run through a gDNA eliminator column (Qiagen) before extraction. The sequencing library was prepared as described for the human transcriptomics and was sequenced using the NextSeq 2000 platform (Illumina Inc, #20038897) using 1000/2000 P2 Reagents (200 Cycles) v3 (#20046812). Coverage of all libraries was variable though the majority of libraries generated ≥33M reads (Min: 29.0M, Max: 49.8M, Mean: 40.7M). Data was deposited in GEO under the accession number GSE246488 (https://www.ncbi.nlm.nih.gov/gds/?term=GSE246488)

### Histology and immunohistochemistry of mouse tissues

Mouse tissues were obtained at different stages after euthanasia by cervical dislocation, anaesthetic overdose or CO_2_ asphyxiation, performed according to protocol guidelines for animal safety. Upon dissection, tracheas, kidneys and brains were fixed in 4% PFA/PBS, testes fixed in Bouin's fixative, and eyes were fixed in Davidson's fixative according to standard protocols. Tissues were serially dehydrated and embedded in paraffin. Samples were sectioned at 5-8 µm and processed for hematoxylin-eosin (H&E) using standard protocols. Wild-type C57BL/6J mice were used as a reference in all knock-in *Tubb4b*^*391His/+*^ and all F0 analyses.

Paraffin embedded eye and trachea tissue sections were dewaxed and re-hydrated via ethanol series prior to antigen retrieval in 10 mM Tris-HCl pH 9.2, 2 mM EDTA, 0.01% Tween-20 for 7 min at 900 W in the microwave. Sections were blocked for 1 h with blocking solution (0.1% Tween, 50 mM NH_4_Cl, 1% BSA and PBS) prior to immunostaining using primary and secondary antibodies detailed in Tables S7,S8. Samples were stained with 1.25 µg/mL DAPI (Roche, Mannheim, Germany), rinsed and mounted in Fluoromount medium (Sigma) under glass coverslips.

### Transmission electron microscopy on tissues-mouse

Samples were dissected into PBS. Samples were fixed in 2 % PFA/2.5 % glutaraldehyde/0.1 M sodium cacodylate buffer pH 7.4 (Electron Microscopy Sciences). Tracheas were fixed for 18 h at 4 °C and then rinsed in 0.1 M sodium cacodylate buffer, post-fixed in 1% OsO_4_ (Agar Scientific) for 1 h and dehydrated in sequential steps of acetone prior to impregnation in increasing concentrations of resin (TAAB Lab Equipment) in acetone followed by 100%, placed in moulds and polymerized at 60 °C for 24 h. Ultrathin sections of 70 nm were subsequently cut using a diamond knife on a Leica EM UC7 ultramicrotome. Sections were stretched with chloroform to eliminate compression and mounted on Pioloform filmed copper grids prior to staining with 1% aqueous uranyl acetate and lead citrate (Leica). They were viewed on a Philips CM100 Compustage Transmission Electron Microscope with images collected using an AMT CCD camera (Deben).

### Whole-mount immunofluorescence

Tracheas, choroid plexuses and oviducts from littermates or age-matched controls were dissected in PBS and fixed in 4% methanol-free formaldehyde for 1 h RT. Samples were permeabilized in PBS/0.5% Triton-X-100 for 15 min and blocked in 4% BSA/PBS/0.025% Tween-20 (PBST). The corresponding antibody incubations were done overnight in 4% BSA/PBST (Tables S7,8). Following washes and staining with (DAPI), samples were mounted in Prolong Gold (Life Technologies, Thermo Fisher Scientific).

Brain ventricles were dissected according to (77), pre-extracted with 0.1% Triton X in PBS for 1 min, then fixed in 4% PFA or ice cold methanol for at least 24 h at 4 °C, followed by permeabilization in PBS (0.5% Triton X-100) for 20 min room temperature. Ventricles were blocked in 4% BSA in PBST (PBS/0.25% Triton X-100) for 1 h at room temperature, then placed ependymal layer down in primary antibodies (Table S7) in 4% BSA/PBST for at least 12 h. Ventricles were washed in PBS 3 X 10 min and incubated with secondary antibodies (Table S8) in 4% BSA/ PBST (0.25% Triton X-100) at 4 °C for at least 12 h. Ventricles were washed in PBS 3 X 10 min, and mounted on glass bottom dishes (Nest, 801002) in Vectashield (VectorLabs), immobilized with a cell strainer (Greiner Bio-One, 542040).

### Isolation and immunofluorescence of primary mouse cells

Mouse tracheal epithelial cells (mTECs) were isolated and cultured as described previously (78, 79). Ependymal cells were isolated from mice aged P0 to P5 and cultured as described previously (80). Mouse fibroblasts were harvested from a mix of tail and ear tissue from P5 mice as described (81). For immunofluorescence, cells were plated on coverslips or in glass bottom plates. Ependymal and fibroblast cells were fixed in 4% methanol-free formaldehyde for 5-10 min. Samples were permeabilized in TBST (TBS/0.1% Triton-X-100) for 5 min, blocked in 5% donkey serum in TBST. The corresponding primary and secondary antibody incubations (Tables S7,8) were done overnight in 1% donkey serum in TBST. Washes were done in TBST and stained with DAPI before mounting in Prolong Gold (Life Technologies, Thermo Fisher Scientific).

### Single cell RNASeq analysis

Published 10X single cell data and metadata was read using the Seurat (82–85) SCTransform method to obtain a gene by cell expression matrix using data from (86–88) or the published gene by cell matrix was used with data from (89, 90). The proportion of cells of each of the cell types, as identified in the data by the respective authors, expressing a tubulin (expression greater than zero) was calculated as a proportion of all cells of that type. The resulting matrix was visualized as a heatmap allowing rows (cell types) to cluster (Fig. S4J (mouse airway, choroid plexus and ependymal datasets), Fig. S10F (mouse and human neuroretina across ages)).

### Quantitative imaging of Tubb4bALFA levels between cilia types

Wild-type (n=2) and heterozygous (n=3) *Tubb4b*^*ALFA/-*^ P8 neonatal littermates were culled by pentobarbital barbiturate intraperitoneal injection. Both trachea and brains were sub-dissected from each animal in ice-cold PBS. Tissues were fixed overnight at 4 °C in 4% PFA/PBS, then rinsed and permeabilised in PBST (PBS, 0.5% Triton-X 100 (ThermoFisher, #85111)) for 20 min and blocked using 2% BSA (Merck, #A9418) in PBS with 0.05% Tween-20 (ThermoFisher, #28320) for 30 min. Primary antibodies (FOP, acetylated α-tubulin and anti-ALFA-Alexa647) were diluted (Table S7) in block solution and incubated overnight at 4 °C. Tissues were washed 3 times with PBS for 5 min per wash. Secondary antibodies (Table S8) were then diluted in block solution, and the tissues incubated with secondary antibodies for 2 h. Tissues were then washed 3 times with PBS prior to mounting. Tracheas were cut into longitudinal strips and mounted onto a glass microscopy slide (Fisher Scientific, #11562203) using ProLong Gold mounting medium (ThermoFisher,#P10144). Brain ventricles were mounted in round glass bottom dishes (Nest, #801002) using ProLong Gold mounting medium and secured with a glass coverslip on top. Images were acquired using Nikon A1+ Confocal (Nikon Europe B.V., Netherlands) with Oil 60x lens using the same PIN, gain and laser intensities between tissues. Single planes shown in Fig. 6G were generated using ImageJ. For quantitative imaging of ratiometric levels, as shown in Fig. 6H, values of pixel intensities from single Z-planes were obtained from NIS Elements (Nikon Europe B.V., Netherlands), by drawing a line across those ciliary bundles that displayed clearest separation between individual cilia (n= 4-8 ciliary bundles per animal per tissue). Pixels with overlapping intensity values for AcTUB and ALFA647 above background were used to calculate the ratios shown in the box plots.

### Imaging

Images for Fig. 1T were acquired using epifluorescent microscopy. Brightfield images were captured on a Hamamatsu Nanozoomer XR (Hamamatsu Photonics, Japan) with 20X and 40X objectives. Confocal Z-stack projections in Fig. 1U,V, Fig. 3A,E,F, Fig. 4K, Fig. S2K-M, Fig. S6 and Fig. S10D were captured on a Spinning Disk Zeiss microscope (Zeiss, Oberkochen, Germany). Confocal Z-stack projections in Fig. S2L,M, Fig. 4H and Fig.2E,F,I were taken on a Nikon A1+ Confocal (Nikon Europe B.V., Netherlands) with oil immersion 60X or 100X objectives with 405, Argon 561 and588, 640 lasers and GaSP detectors. 3D reconstructions of images in Fig. S3F and Fig. S4J were captured with an Andor Dragonfly and Mosaic Spinning Disc confocal using Nikon oil 40X or 100X lenses. Fig. 6D-G (regular mode), Fig. S2N,O (lightning mode), Fig. S3G (lightning mode) and Fig. S4A,D (lightning mode) taken on Leica STELLARIS 8 Spectral confocal microscope equipped with a White Light Laser enabling tuneable excitation wavelengths between 440-790 nm with a 405 nm diode laser for UV. Flexible fluorophore detection occurs via two HyD S (Silicone Multi-Pixel Photon Counter) & two Power HyD X (GaAsP Hybrid) detectors (Leica Microsystems UK Ltd, Milton Keynes, UK) with an oil immersion 63X or 100X NA lens. Basal body quantification data was acquired using the Leica LASX acquisition software with the lightning deconvolution setting. High-speed video microscopy for mouse samples was performed on a Nikon Ti microscope with a 100X SR HP Apo TIRF Objective, and Prime BSI, A19B204007 camera, imaged at 250 fps. Projections, 3D reconstructions or panels were generated using ImageJ (National Institutes of Health), NIS Elements (Nikon Europe B.V., Netherlands), NDP.view2 (Hamamatsu Photonics, Japan) or Imaris software 9.9 (Oxford Instruments, UK). Final composite images were generated using FigureJ plugin on ImageJ software (National Institutes of Health, Bethesda, MA, USA) (91), Photoshop or Illustrator (Adobe Systems, San Jose, CA).

### Data analysis

Automated quantification of basal bodies from wholemount tracheas, lateral ventricles and oviducts using a macro in FIJI (National Institutes of Health) to count Centrin2-GFP fluorescence intensity maxima in user-defined cells, the script used can be found on Zenodo (92). Cilia lengths for Fig. S9B were measured using NDP.view2 (Hamamatsu Photonics, Japan) while cilia lengths for IF images in Fig. S3, S4 were measured using FIJI’s line tool (National Institutes of Health). Data analysis was carried out in Microsoft Excel, GraphPad Prism 9 (version 9.4.1, GraphPad Software, USA) and Matlab. Statistical tests are described in the figure legends and methods sub-sections.

## Supplementary Material

Movie S1

Movie S2

Movie S3

Movie S4

Movie S5

Movie S6

Supplementry Materials

## Data Availability

Proteomics datasets can be found on ProteomeXchange with identifier PXD036304 (http://central.proteomexchange.org/cgi/GetDataset?ID=PXD036304). Human RNA sequencing can be found at GEO datasets under accession GSE214070 (https://www.ncbi.nlm.nih.gov/geo/query/acc.cgi?acc=GSE214070) while mouse RNA sequencing can be found using the accession GSE246488 (https://www.ncbi.nlm.nih.gov/gds/?term=GSE246488). The cryo-EM map of a TUBA1A-TUBB4B heterodimer from human respiratory doublet microtubules has been deposited in the Electron Microscopy Data Bank under accession code EMD-40480. The atomic model of the TUBA1A-TUBB4B heterodimer has been deposited in the Protein Data Bank under accession code 8SH7. All materials in this project will be made available to any researcher upon request for purposes of reproducing or extending the analysis, either through a repository or directly from the researchers. The script for basal body quantification can be found at Zenodo (92).

## One-Sentence Summary

Identification of organelle-specific requirements for the TUBB4B β-tubulin isotype in building functional cilia in human health and disease.
