# Supplementary material for: Ciliopathy patient variants reveal organelle-specific functions for TUBB4B in axonemal microtubules
Source: Science. Author manuscript; Available in PMC 2024 Jul 16. (PMC7616230; doi:10.1126/science.adf5489)
Supplement: Supplementry Materials [file EMS196953-supplement-Supplementry_Materials.pdf]

Supplementary Materials:

Figs. S1 to S15

Tables S1 to S8

Movies S1 to S6

Science
AAAS

Supplementary Materials for

Ciliopathy patient variants reveal organelle-specific functions for TUBB4B in axonemal microtubules

Daniel O Dodd[1†], Sabrina Mechaussier[2†], Patricia L Yeyati[1], Fraser McPhie[1], Jacob R Anderson[3], Chen Jing Khoo[4], Amelia Shoemark[5,6], Deepesh K Gupta[7], Thomas Attard[8], Maimoona A Zariwala[9], Marie Legendre[10,11], Diana Bracht[12], Julia Wallmeier[12], Miao Gui[3, ¶], Mahmoud R Fassad[13,14], David A Parry[1], Peter A Tennant[1], Alison Meynert[1], Gabrielle Wheway[15], Lucas Fares-Taie[2], Holly A Black[16,17], Rana Mitri-Frangieh[18,19], Catherine Faucon[18], Josseline Kaplan[2], Mitali Patel[13,20], Lisa McKie[1], Roly Megaw[1,21], Christos Gatsogiannis[22], Mai A Mohamed[13,23], Stuart Aitken[1], Philippe Gautier[1], Finn R Reinholt[24], Robert A Hirst[25], Chris O'Callaghan[26], Ketil Heimdal[26], Mathieu Bottier[5], Estelle Escudier[11,18], Suzanne Crowley[27], Maria Descartes[28], Ethylin W Jabs[29,30], Priti Kenia[31], Jeanne Amiel[32,33], Giacomo Maria Bacci[34], Claudia Calogero[34], Viviana Palazzo[35], Lucia Tiberi[36], Ulrike Blümlein[37], Andrew Rogers[6], Jennifer A Wambach[7], Daniel J Wegner[7], Anne B Fulton[38], Margaret Kenna[39], Margaret Rosenfeld[40], Ingrid A Holm[41,42], Alan Quigley[43], Emma A Hall[1], Laura C Murphy[1], Diane M Cassidy[5], Alex von Kriegsheim[44], Scottish Genomes Partnership[16], Genomics England Research Consortium[45], Undiagnosed Diseases Network[46], Jean-François Papon[47], Laurent Pasquier[48], Marlène S Murris[49], James D Chalmers[5], Claire Hogg[6], Kenneth A Macleod[48], Don S Urquhart[48,49], Stefan Unger[50,51], Timothy J Aitman[16], Serge Amselem[10,11], Margaret W Leigh[52], Michael R. Knowles[53], Heymut Omran[12], Hannah M Mitchison[13], Alan Brown[3], Joseph A Marsh[1], Julie P I Welburn[8], Shih-Chieh Ti[4], Amjad Horani[7,54], Jean-Michel Rozet[2], Isabelle Perrault[2*], Pleasantine Mill[1*]

Correspondence to: isabelle.perrault@inserm.fr
pleasantine.mill@ed.ac.uk

This PDF file includes:

Figs. S1 to S15
Tables S1 to S8
Captions for Movies 1 to 6

Other Supplementary Materials for this manuscript include the following:

Movies 1 to 6

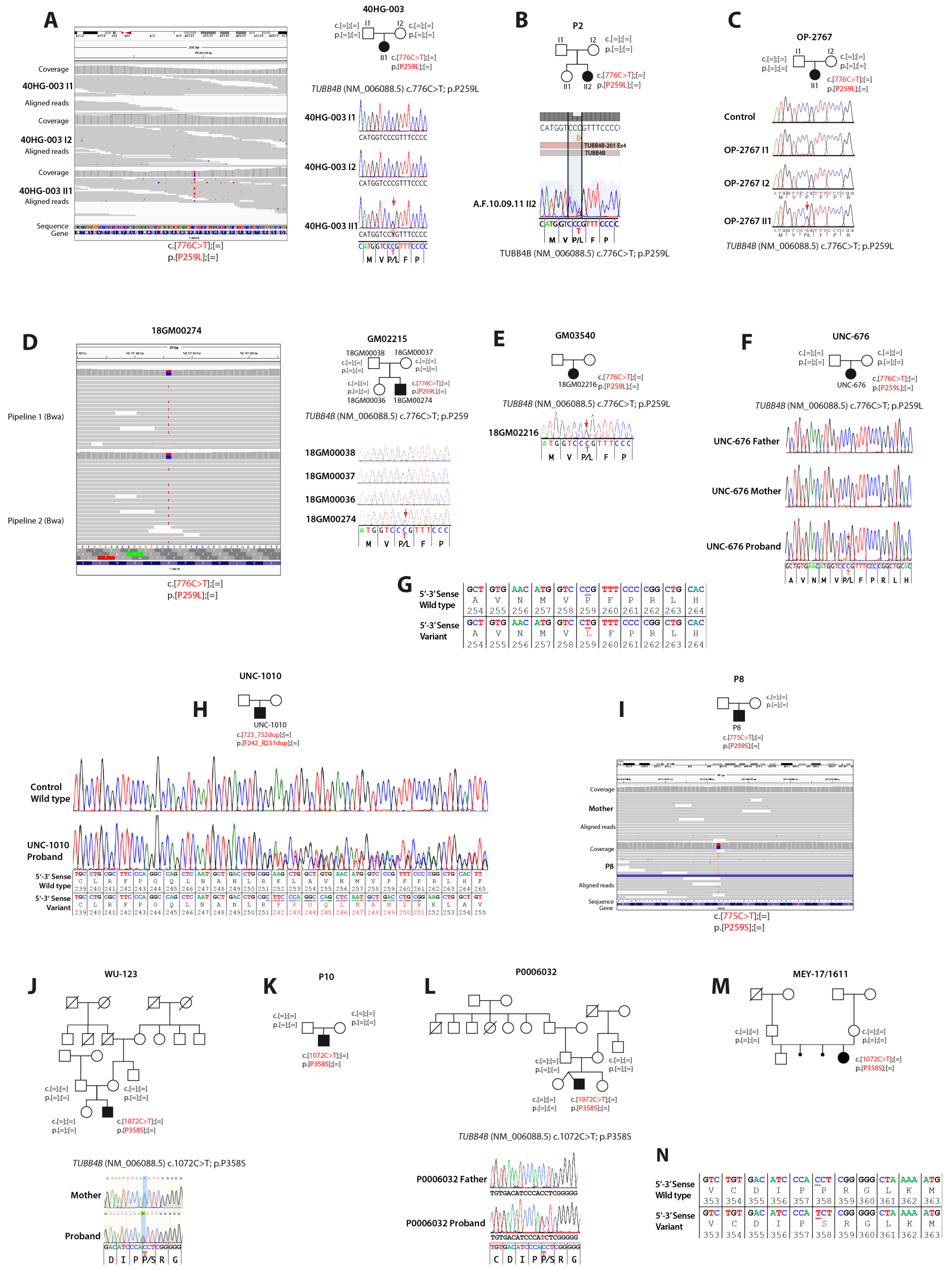

Fig. S1.

Segregation and location of pathogenic variants in *TUBB4B* identified in unrelated PCD patients.

(**A**) P1 (from family 40HG-003) was recruited for WGS based genetic diagnosis of PCD which revealed a heterozygous de novo missense mutation p.P259L (chr9:g.137242994:C>T (hg38)) in *TUBB4B*

(NM_006088.6). Mutation and de novo pattern of segregation of the allele were confirmed by targeted Sanger sequencing.

(B) P2 is proband of a family initially screened for 34 PCD genes including *CCNO* and *MCIDAS* by high-throughput sequencing, followed by screening of a UCL Great Ormond Street ICH targeted gene panel of 40 PCD and 400 motile cilia genes. Whilst biallelic mutations in *DNAH9* were identified comprising a splice acceptor mutation (NM_001372.3:c.7553-3del) and exonic missense mutation (NM_001372.3:c.12640G>T, p.Gly4214Cys), the cellular phenotype of reduced generation of motile cilia was not in keeping with pathogenic variants in this gene (*91)*. Reanalysis of sequencing data confirmed a heterozygous *TUBB4B* de novo missense mutation p.P259L (chr9:137242994:C>T (hg38)) only in the patient, and not present in either parent.

(C) P3 (from trio OP-2767) was recruited for WES-based genetic diagnosis of PCD, followed by targeted analysis of variants in TUBB4B, which identified a heterozygous 'de novo' missense variant [c.776C>T; p.P259L] in exon 4 in the patient that was not present in either parent or a non-related control.

(D) P4 (from family GM02215) was recruited for WES genetic diagnosis of PCD, which identified a heterozygous *de novo* missense mutation p.P259L (g.chr9:137242994:C>T (hg38)) in *TUBB4B*. Mutation confirmation and segregation of the allele were performed by targeted analysis of the *TUBB4B* locus using Sanger sequencing.

(E) P5 is proband 18GM02216 recruited as a singleton for genetic diagnosis of PCD. After a negative targeted capture sequencing of 50 PCD genes, Sanger sequencing of *TUBB4B* identified a heterozygous missense mutation p.P259L (g.chr9:137242994:C>T (hg38)). Parents were not tested.

(F) P6 (from trio UNC-676) was recruited for WES followed by targeted analysis of variants in *TUBB4B* which identified a heterozygous de novo missense variant [c.776C>T; p.P259L] in exon 4. Both the parents were also tested and confirmed to be wild type (WT) at this locus.

(G) Summary of variants identified in all patients (P1-P6) as heterozygous for [c.776C>T; p.P259L] missense variant in exon 4 (below) compared to control sequence (above). Base sequence, amino-acid sequence and codon numbers are shown. Location of base substitution is underlined and amino-acid substitution is depicted in red.

(H) WES followed by targeted analysis of variants in *TUBB4B* (NM_006088.6) identified a heterozygous in-frame 30 bp duplication variant [c.723_752dup; p.(F242_R251dup)] in exon 4 in patient P7, that was verified by Sanger sequencing (individual UNC-1010, lower chromatogram, compared to a healthy individual). DNA from the parents of UNC-1010 was not available for the segregation analysis.

(I) Proband P8 was recruited as a duo where a variant p.P259S (chr9:137242993:C>T (hg38)) was identified in *TUBB4B* only in the patient, and was not present in the mother. DNA from the father was not available for segregation analysis.

(J) P9 (from family WU-123) was recruited for WES to diagnose profound sensorineuronal disease (SND) including early onset vision and hearing loss, as well as respiratory disease consistent with PCD. A variant [c.1072C>T; p.P358S] in exon 4 of the *TUBB4B* gene was identified in only the patient sample in a heterozygous state, confirming de novo inheritance. Results confirmed by Sanger sequencing.

(K) Proband P10, recruited with a history of bilateral sensorineural disease (SND), chronic kidney disease with nephromegaly and hypertension, chronic productive cough and a history of recurrent sinus and ear infections consistent with PCD. The patient also has short stature. A variant [c.1072C>T; p.P358S] in exon 4 of *TUBB4B* was identified in only the patient sample in a heterozygous state, confirming de novo inheritance.

(L) P11 (from family P0006032) was recruited with a history of CHD (cardiomyopathy), as well as hearing and sight loss (SND), plus PCD features such as severe inner ear infections. WES was performed on the

mother, twin sister and proband initially, which identified the variant [c.1072C>T; p.P358S] in exon 4 of *TUBB4B*. De novo inheritance was confirmed by Sanger sequencing.

(M) P12 is proband 17/1611 recorded as a trio for WES following bilateral sensorineural disease (SND), features of PCD, CHD at birth (perimembranous ventricular septal defect), short stature and renal dysplasia with a mild eGFR reduction. A variant [c.1072C>T; p.P358S] in exon 4 of *TUBB4B* was identified in only the patient sample in a heterozygous state, confirming de novo inheritance.

(N) Summary of variants identified in all patients (P9-P12) as heterozygous for [c.1072C>T; p.P358S] missense variant in exon 4 (below) compared to control sequence (above).

In all pedigrees, males and females are designated by the squares and circles, respectively. Filled symbols represent affected probands. Small, filled circles represent spontaneous abortions. Red arrow above chromatograms highlights the affected residue.

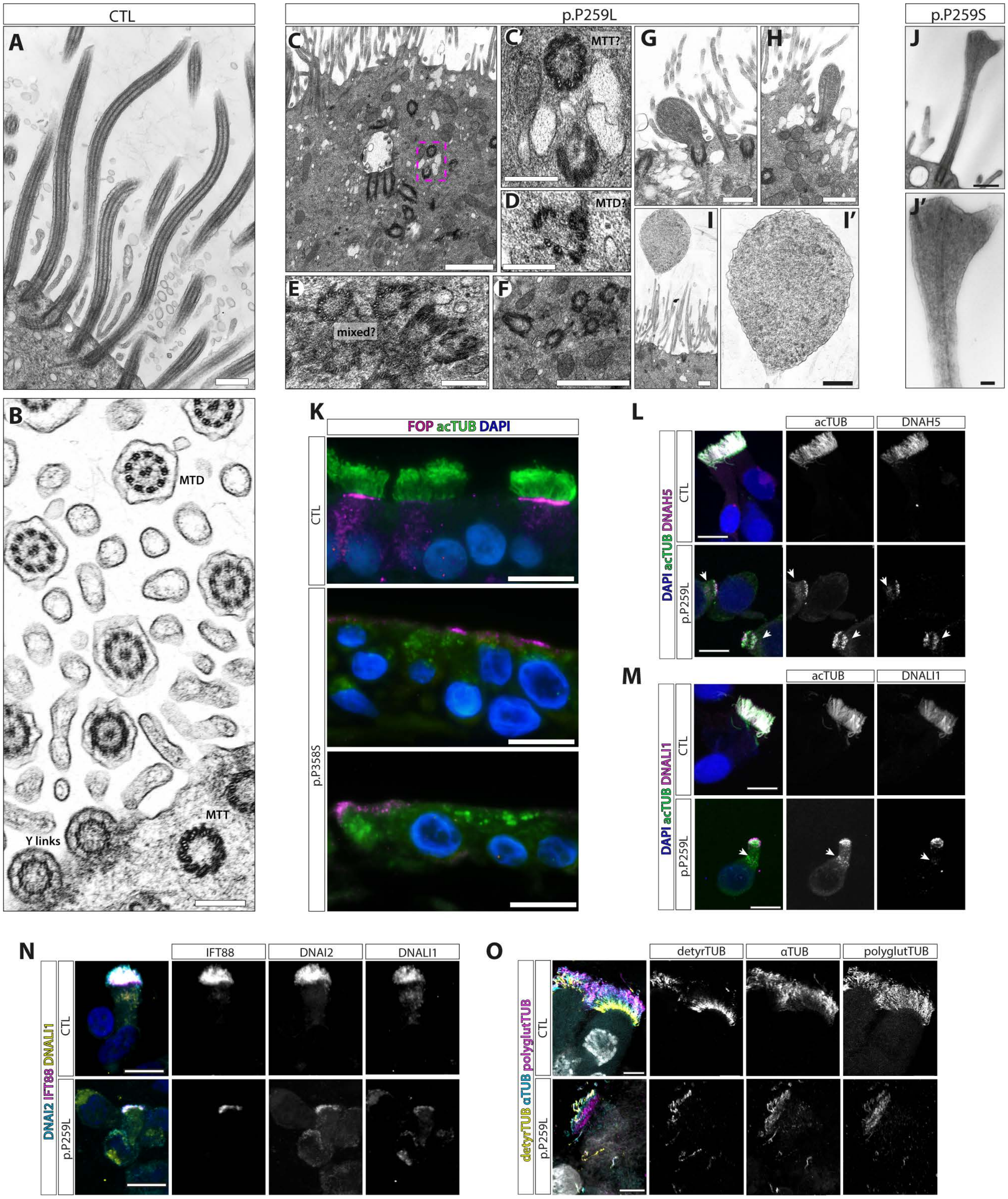

Fig. S2.

***TUBB4B* variants affect ciliary number, length and microtubule PTMs.**

(**A-J'**) TEM of nasal brush epithelial cells from control (**A,B**) and PCD patients with p.P259L variant (P3, **C-I'**) or p.P259S (P8, **J,J'**). In control cells, dense, long cilia on the surface (**A,B**), which in cross-section clearly transition through docked basal body (MTT- microtubule triplets), transition to microtubule doublets (MTD) exiting the cell with characteristic Y-links of transition zone and then to '9+2' MTD

of the axoneme. In PCD patient cells evidence of disrupted centriole assembly and amplification is observed including misoriented and internally docked centrioles without axonemes as well as partial structures (**C-F**). Dashed magenta ROI in (**C**) with increased zoom to highlight features (**C'**). (**G-I'**) P3 TEM shows rare short cilia without clear axonemal microtubules (**G,H**) or dilated cilia tips with disorganized microtubules and granular material (**I,I'**). (**J,J'**) P8 TEM of short cilia with bulbous head shows splayed microtubules within the expanded tip.

(**K**) Nasal brush epithelial cells were cultured and differentiated in ALI from healthy mother (CTL) and syndromic PCD (P9) patient (lower) before sectioning for immunofluorescence confirming reduced centrioles (FOP: magenta) and loss of axonemes, with abnormal acetylated α-tubulin staining (green, arrowheads) mislocalizing within the cytoplasm.

(**L,M**) Immunofluorescence of healthy donor (CTL) or patient (P2) nasal brushings stained for cilia (acTUB-green) outer (DNAH5: magenta, **L**) and inner dynein arm motors (DNALI1: magenta, **M**) single channels for Fig. 1U,V.

(**N,O**) Immunofluorescence of healthy donor (CTL) or patient (P1) nasal brushings stained for (**N**) cilia outer and inner dynein arm motors (DNAI2: cyan; DNALI1: yellow) of the IFT-B complex (IFT88: magenta) and (**O**) microtubule post-translational modifications (α-tubulin: cyan; polyglutamylated tubulin: magenta; detyrosinated tubulin: yellow). Scale bars represent: 10 μm (**K-M**), 5 μm (**N,O**), 1 μm (**C,F**), 500 nm (**J**), 250 nm (**A,B, C'-E, G,-I'**), and 100 nm (**J'**).

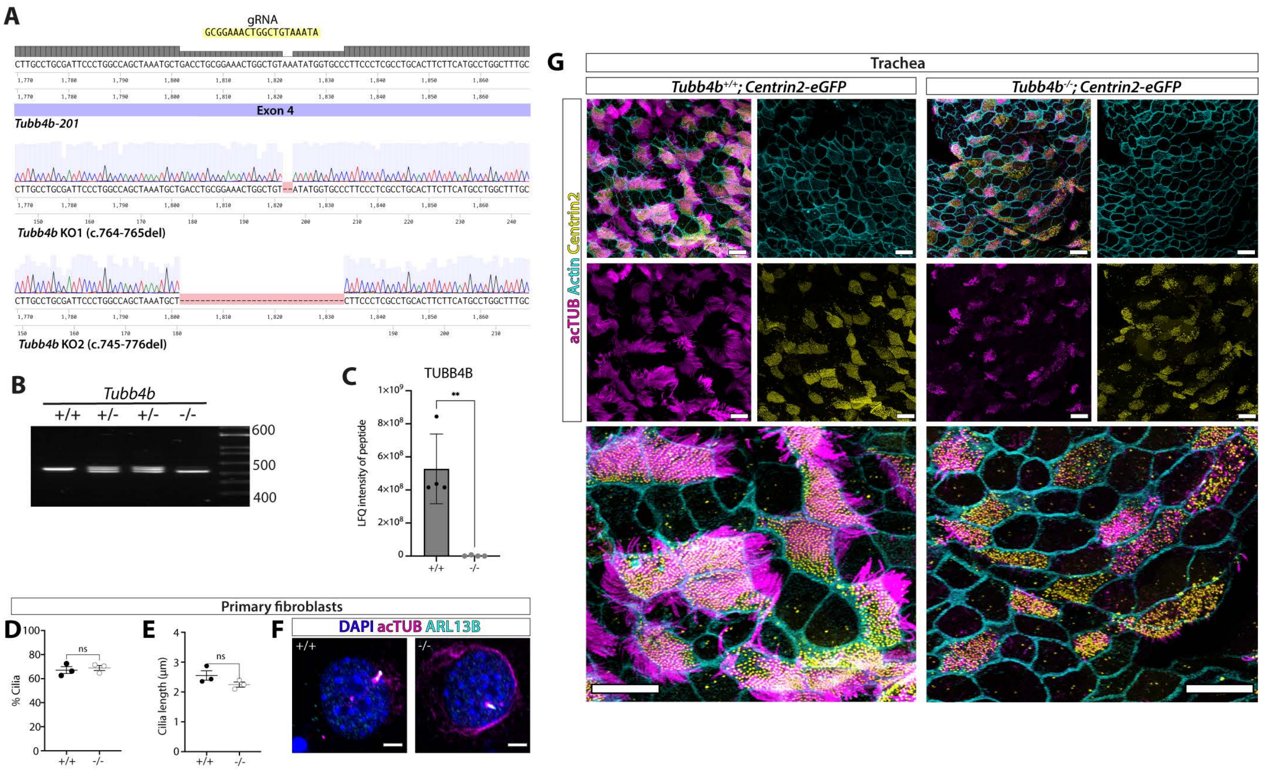

Fig. S3.

Generation of *Tubb4b* knock-out mice.

(**A,B**) Two independent deletion alleles were generated by Cas9 genome editing in the 4th exon of *Tubb4b* and founder screening. Both alleles cause a frame shift and premature termination codon. (**C**) Both alleles are protein null for TUBB4B unique peptides by mass spectrometry (shown for KO2).

(**D-F**) *Tubb4b*$^{-/-}$ primary cilia on fibroblasts show no difference in percentage ciliation (**D**), cilia length (**E**) or gross structure by immunofluorescence (**F**).

(**G**) In situ wholemount immunofluorescence of *Tubb4b;Centrin2-eGFP* neonatal (P5-P8) tracheas.

Cilia length and number of ciliated cells in fibroblasts were quantified using ARL13B as a marker, N = 3 biological replicates per genotype with (**D**) n>139 cells per biological replicate and (**E**) n>87 cells per biological replicate, mean values are plotted. (**F,G**) Acetylated α-tubulin: magenta, ARL13B: cyan (**F**) or actin: cyan (**G**), Centrin2-eGFP: yellow (**G**).

Scale bars represent: 10 μm (**G**) and 5 μm (**F**). (**C**) Graphic bars represent the mean ± SEM derived from N=4 biological replicates (**C**) or N=3 biological replicates (**D,E**). Statistical analyses were carried out by the PLSD Fisher test according to the significance of the Student's t-test. Student's t-test: ns, not significant; **, p ≤ 0.01.

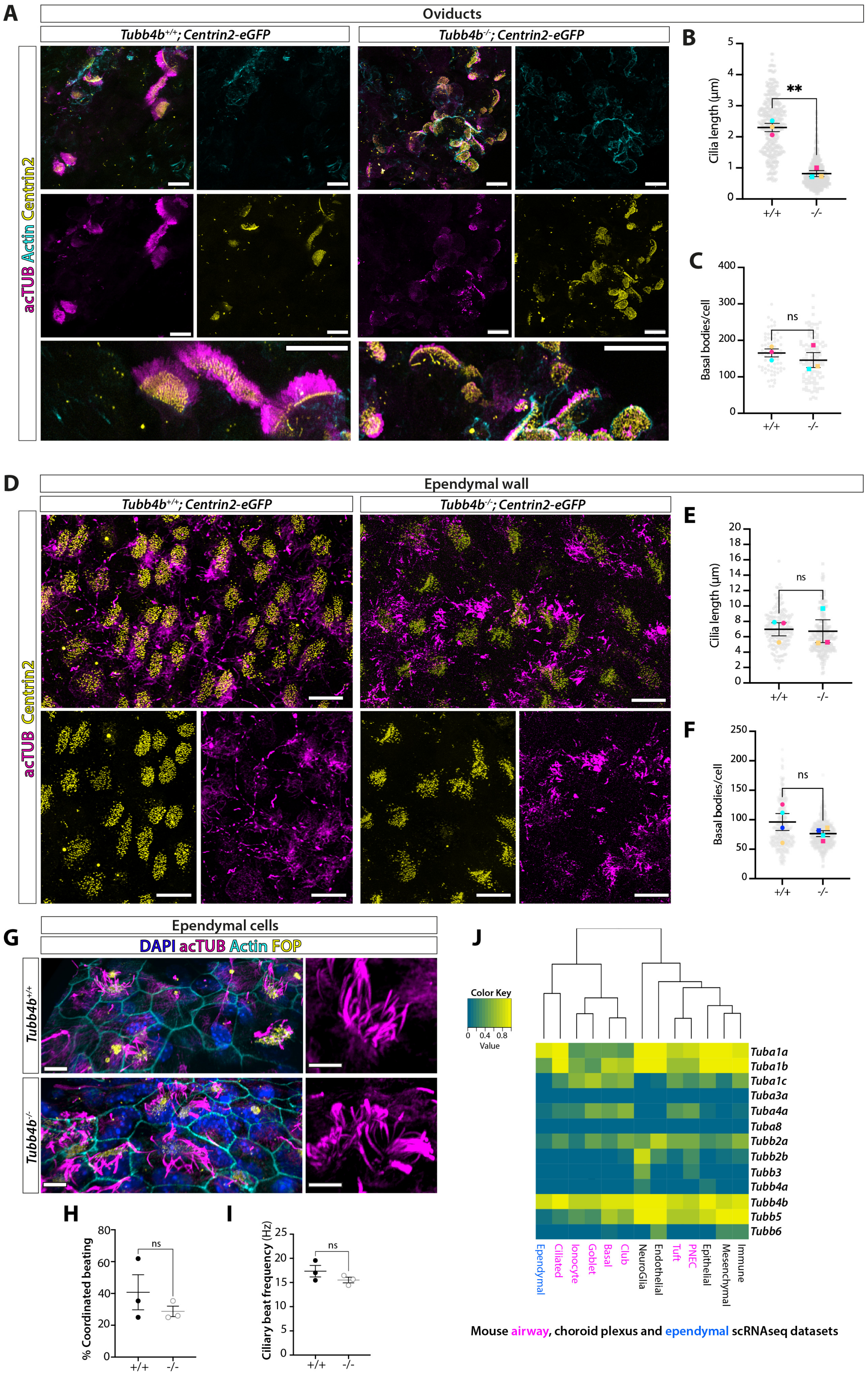

Fig. S4.

Cilia phenotypes vary amongst motile ciliated tissues.

(**A-C**) In situ wholemount immunofluorescence (**A**) of *Tubb4b;Centrin2-eGFP* juvenile (P8-P13) oviducts reveals a profound reduction in cilia length (**B**), but no significant reduction in basal body number per cell (**C**).

(**D-F**) Despite the pronounced hydrocephaly phenotypes visible *in vivo*, in situ flatmount immunofluorescence (**D**) of *Tubb4b;Centrin2-eGFP* lateral ventricles neonates (P5-P8) reveals no reduction in cilia length (**E**) or basal body number per cell (**F**).

(**G-I**) Similarly mutant primary ependymal cells differentiated *in vitro* show no gross difference by immunofluorescence (**G**), nor does high-speed video microscopy reveal changes in percentage of cells with coordinated beating (**H**) or ciliary beat frequency (**I**).

(**J**) Single cell RNASeq heatmaps show the proportions of cells expressing each α- and β-tubulin isotype (RNA count greater than zero) in each cell type identified in the population from published mouse lung (magenta) (*86*), ependymal (blue) (*85*) and choroid plexus (black) (*87*) single cell RNASeq datasets. Rows are clustered on similarity of proportions among cell types (scale 0-1).

Cilia length and number of basal bodies in wholemount oviducts (**A**) and ependymal walls (**D**) were quantified using acTUB as an axoneme marker and eGFP (Centrin2) as a basal body marker. In oviducts, N = 3 biological replicates per genotype with (**B**) n>20 cilia per biological replicate and (**C**) n>62 cells per biological replicate, mean values are plotted. In ependymal flatmounts, N = 4 biological replicates per genotype with (**E**) n>48 cilia per biological replicate and (**F**) n>56 cells per biological replicate, mean values are plotted. (**A, D, G**) Acetylated α-tubulin: magenta, actin: cyan (**A, G**), Centrin2-eGFP: yellow (**A, G**) or Fibroblast Growth Factor Receptor 1 Oncogene Partner (FOP): yellow (**D**).

Scale bars represent: 10 μm (**A, D, G**). (**B, C, E, F, H, I**) Symbols represent the mean ± SEM derived from N=3 biological replicates (**B, C, E, H, I**) or N=4 biological replicates (**F**). Statistical analyses were carried out by the PLSD Fisher test according to the significance of the Student's t-test. Student's t-test: ns, not significant; **, $p \leq 0.01$.

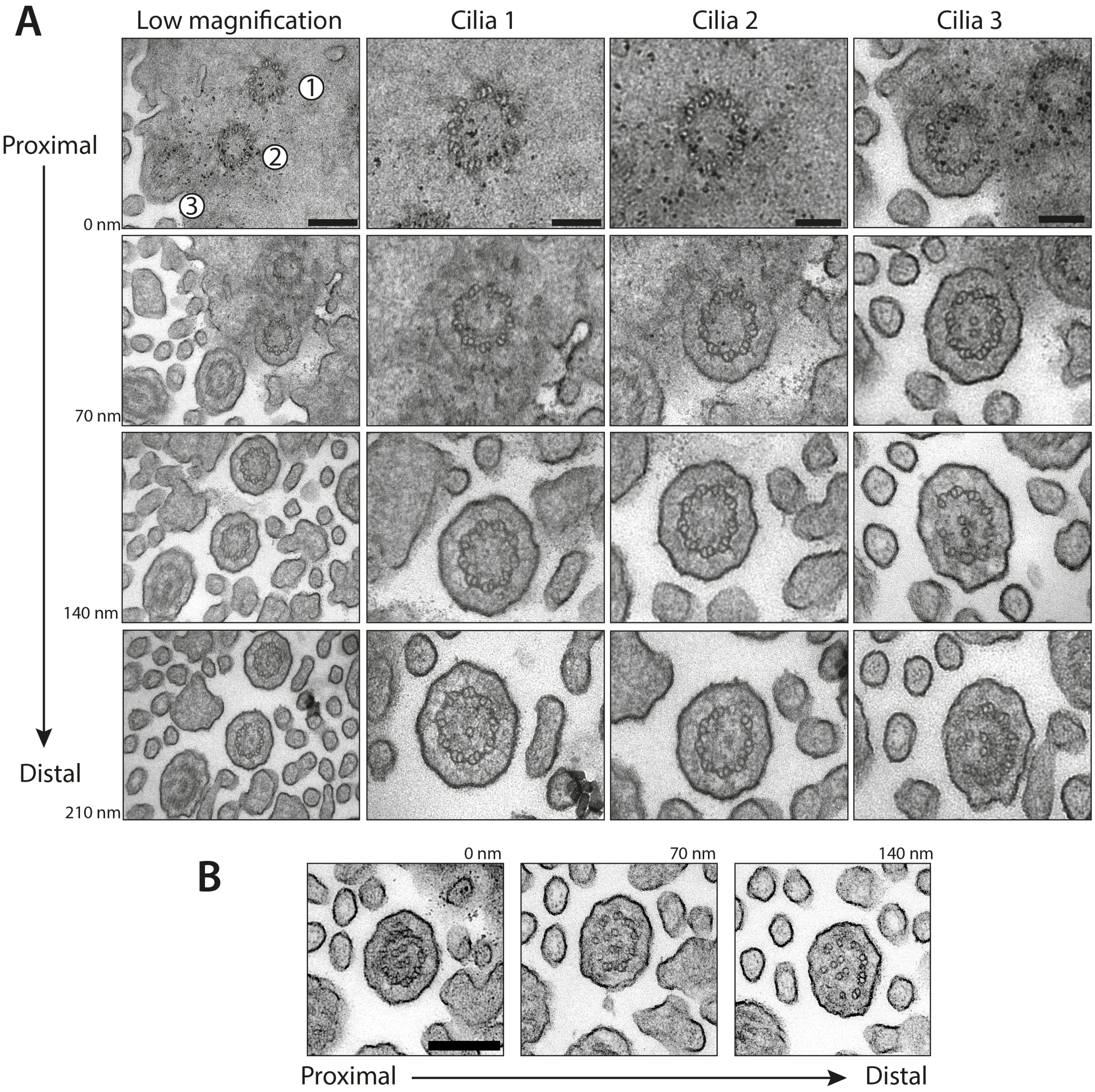

Fig. S5

Serial sections reveal diverse axonemal microtubule defects originate distally to basal bodies.

(A) Tilt series of 70 nm serial sections of *Tubb4b*$^{-/-}$ tracheal epithelial cells tracking 3 adjacent centrioles. Although centrioles are reduced in number and many misformed, those that do dock and extend an axoneme start with 9 microtubule triplets but develop a range of defects including loss of one singlet from central pair (**Cilia 1**), loss of 1 microtubule doublets and lack of central pair (**Cilia 2**) and loss of multiple microtubule doublets (**Cilia 3**). This happens close to the cell body as shown by surrounding microvilli.

(B) 9+4 organization is rapidly lost in 70 nm serial sections.

Scale bars: 200 nm (left row **A, B**), and 100 nm (**A**, Cilia 1-3 inserts).

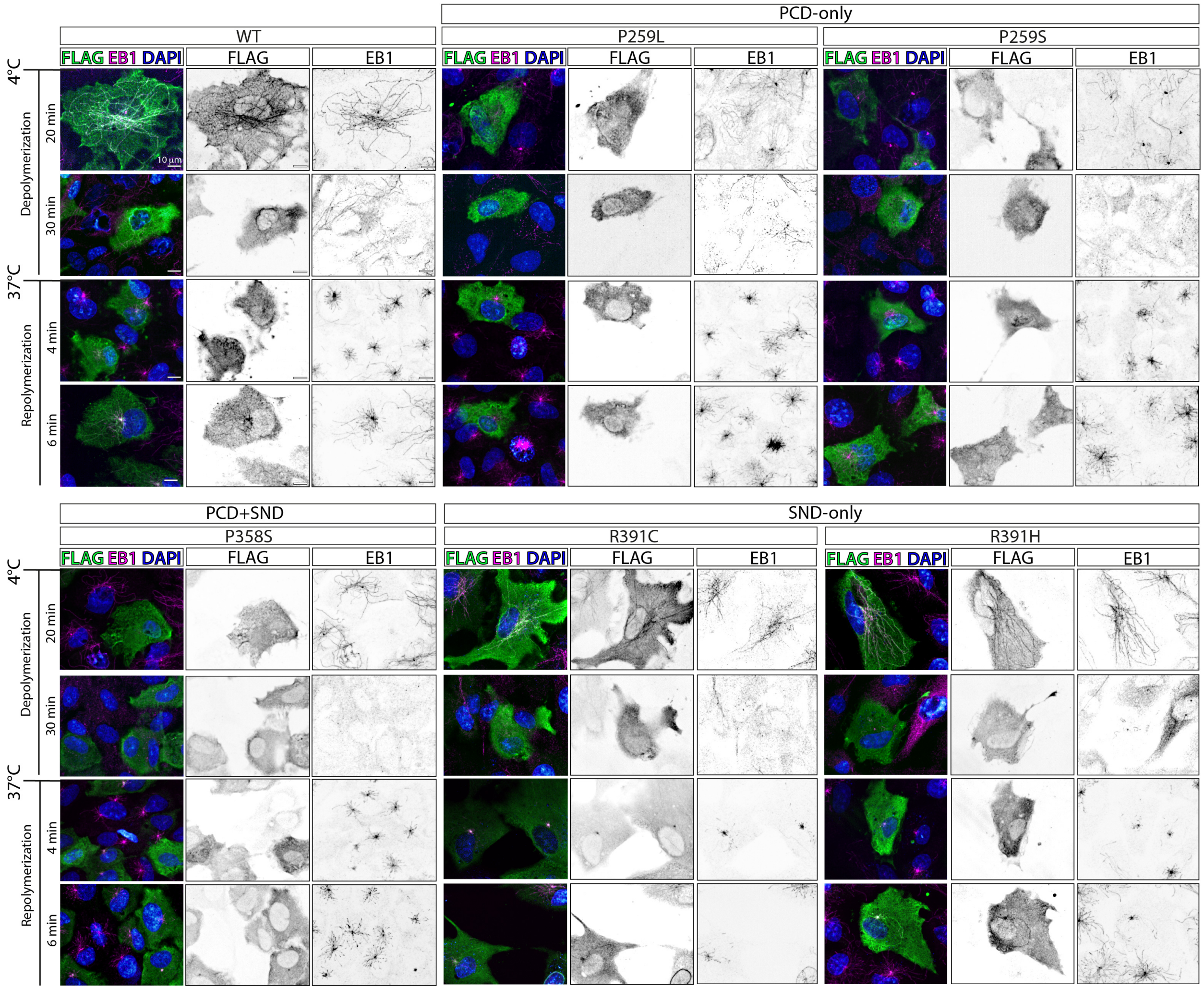

Fig. S6.

Disease-causing TUBB4B variants alter microtubule dynamics. Microtubule network dynamics analysis of RPE1 cells overexpressing TUBB4B variants, showing immunostaining of FLAG-tagged TUBB4B (green) and EB1 (magenta) protein upon cold-induced depolymerization (20 and 30 minutes) and repolymerization at 37 °C (4 and 6 minutes). See **Fig. 3.G, H** for quantification of repolymerization. Scale bars represent: 10 µm.

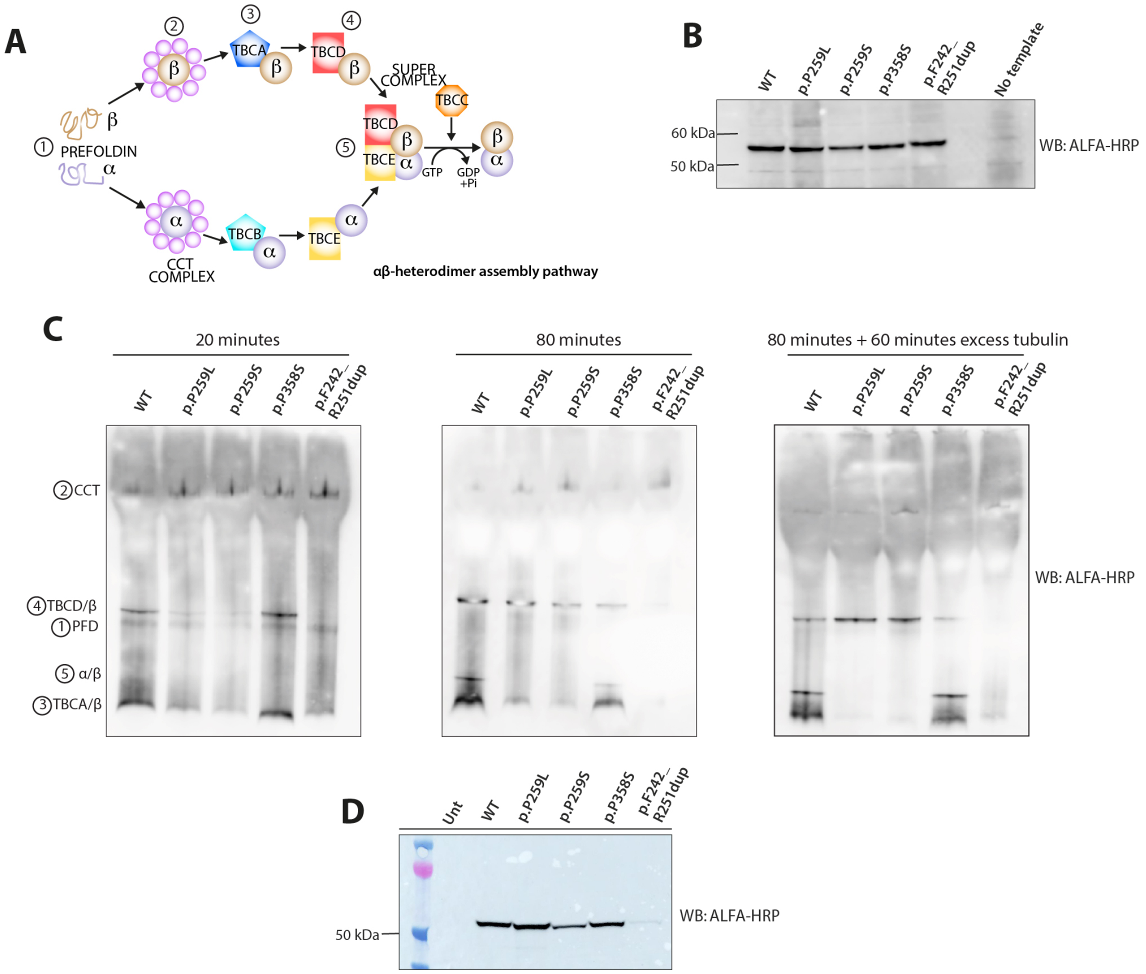

Fig. S7.

PCD-only *TUBB4B* variants affect tubulin heterodimerization.

(A) Immunoblot (denaturing) of *in vitro* translated ALFA-tagged control and *TUBB4B* patient variants. All PCD-only (p.P259L, p.P259S, Dup) and syndromic PCD+SND p.P358S *TUBB4B* variants are stable.

(B) Schematic of tubulin heterodimer assembly pathway. Binding of partially folded tubulin molecules as they emerge from the ribosomes by prefoldin (**1**) and subsequent folding by the cytoplasmic chaperonin CCT (**2**). These quasi-native tubulin intermediates interact with five tubulin-specific chaperones named tubulin cofactors A through E (TBCA–TBCE: **3-5**)). The native assembly-competent tubulin is released from a supercomplex that contains both α- and β-tubulin and cofactors C–E, upon hydrolysis of GTP by β-tubulin in the supercomplex.

(C) Native immunoblot of *in vitro* translated reactions for 20 minutes, 80 minutes and 80 minutes + 60 minutes with excess porcine brain tubulin, respectively. The chaperones are numbered according to their position in the folding pathway. While both WT and syndromic p.P358S TUBB4B form heterodimers robustly, the PCD-only variants stall with different heterodimerization cycle chaperones. The P259L/S stall with TBCD, whilst Dup mutant stalls with Prefoldin and CCT complex chaperones, and neither can be forced through with excess tubulin.

(D) Immunoblot of protein lysate input for affinity purifications in **Fig. 3I-K** from IMCD3 cells stably expressing ALFA-tagged *Tubb4b* constructs probed with ALFA-HRP.

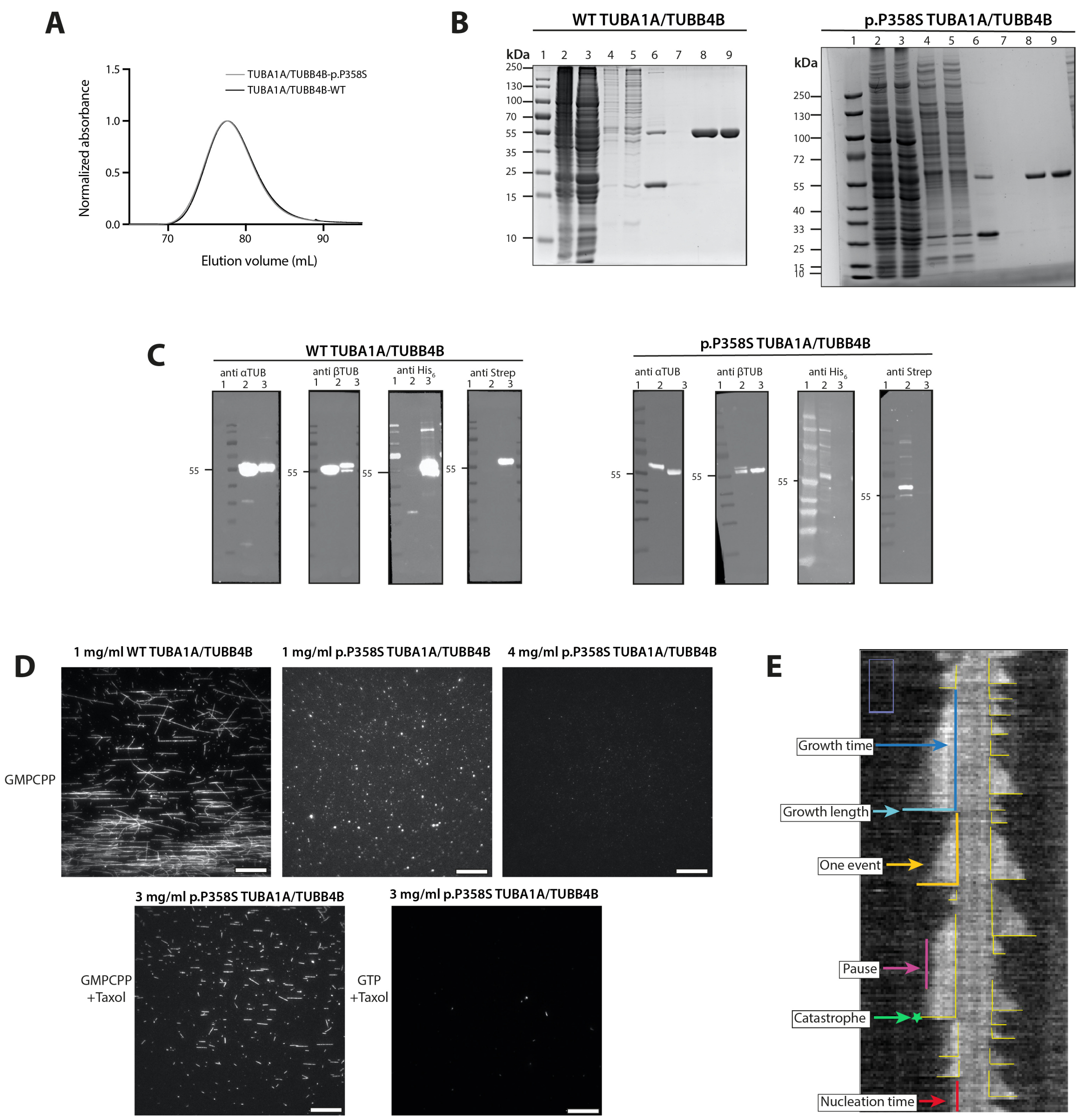

Fig. S8.

PCD+SND *TUBB4B* variant strongly affects tubulin polymerization in a dominant-negative fashion.

(**A**) Elution profiles from the size exclusion chromatography of the affinity tag-free WT (black) and p.P358S (grey) recombinant TUBA1A-TUBB4B heterodimers.

(**B**) Coomassie-stained SDS-PAGE analysis of protein samples from purification steps as follows: (1) ladder; (2) whole cell lysates; (3) flowthrough from histidine affinity column; (4) elution from histidine affinity column; (5) flowthrough from engineered streptavidin (Strep-Tactin XT) column; (6) elution from engineered streptavidin (Strep-Tactin XT) column; (7) flowthrough from anion exchange (HiTrap Q FF) column; (8) elution from anion exchange (HiTrap Q FF) column; and (9) gel filtration.

(**C**) Immunoblot analyses using anti-α-tubulin, anti-β-tubulin, anti-His_6 and anti-Strep antibodies. The loading order for wild type: (1) ladder; (2) gel filtration and (3) elution from histidine affinity column. Loading for p.P358S follows: (1) ladder; (2) elution from histidine affinity column; and (3) gel filtration.

(D) Representative TIRF-microscopy images of tubulin solutions that were incubated at 37 °C for 90 minutes. 1 mg/ml wild-type TUBA1A-TUBB4B heterodimers readily polymerized in the presence of 1 mM GMPCPP (upper left panel), whilst p.P358S TUBA1A-TUBB4B heterodimers failed to polymerize at 1 mg/ml (upper centre panel) or 4 mg/ml (upper right panel) under the same condition. In the presence of 10 μM taxol, 3 mg/ml p.P358S TUBB4B/TUBA1A can polymerize into microtubules with 1 mM GMPCPP (bottom left panel), but not with 1 mM GTP (bottom right panel), suggesting the competence of p.P358S variant TUBA1A-TUBB4B heterodimers to form a microtubule lattice but with a much higher critical concentration than wild-type TUBA1A-TUBB4B heterodimers. Scale bars represent 20 μm.

(E) Representative TIRF kymograph generated during one imaging session on one microtubule fiber in vitro highlighting parameters used to measure microtubule dynamics in **Fig. 3L-Q.** This includes polymerization rate (growth length (cyan)/ growth time (blue)), pause fraction (total pause time (magenta)/total growth time), catastrophe frequency (total number of catastrophe events (green stars)/lifetime) and nucleation frequency (total number of nucleation events (red)/ nucleation time (red)).

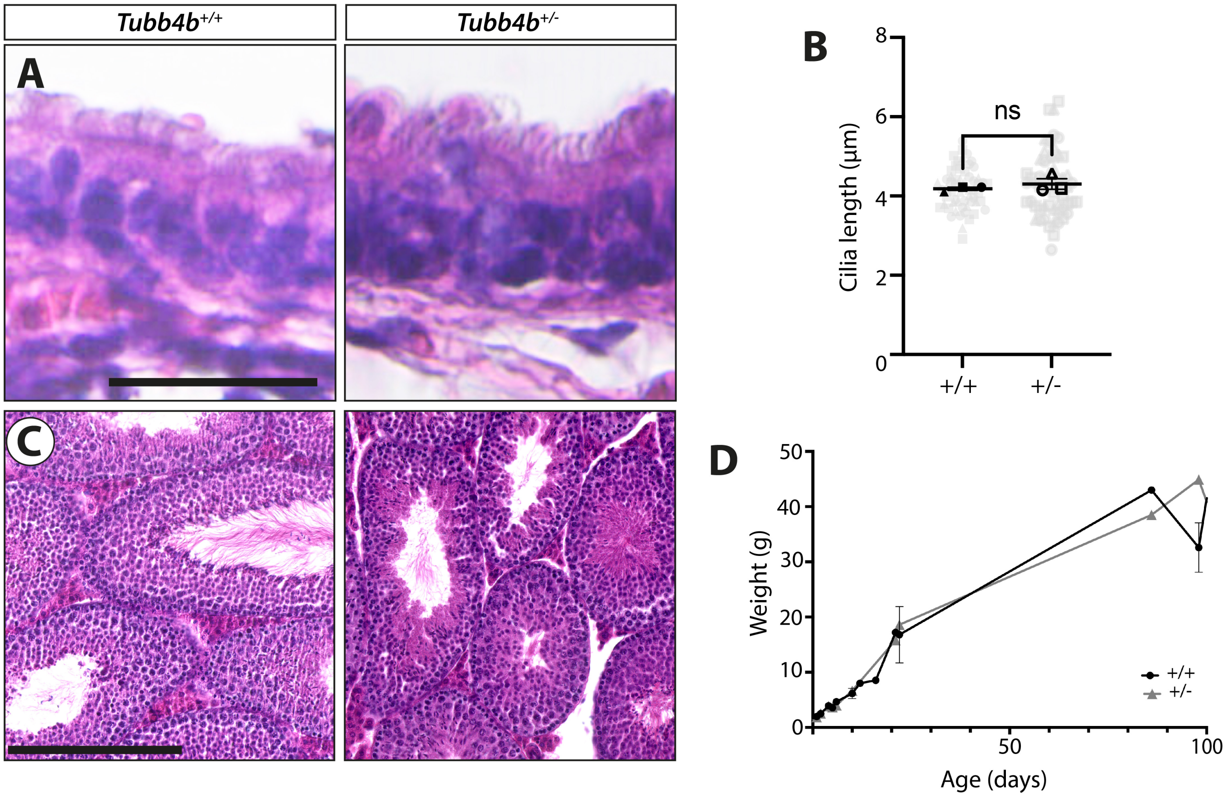

Fig. S9.

Heterozygous loss-of-function alleles for *Tubb4b* have no phenotype.

(**A-D**) Heterozygous KO animals (shown for KO2) are phenotypically normal, showing normal trachea cilia lengths (**A**) and numbers (**B**), normal fertility, as shown by normal spermatogenesis (**C**), and postnatal survival/growth (**D**).

Cilia length was quantified by histology, N = 3 biological replicates per genotype (**B**) n > 18 cells per biological replicate, mean values are plotted. Plot in (**D**) represents N=155 animals. In (**E**), symbols represent individual animals whereas graphic bars represent the mean ± SEM derived from those biological replicates per genotype where n=5 (*Tubb4b*$^{+/+}$), n=4 (*Tubb4b*$^{+/-}$) and n=3 (*Tubb4b*$^{-/-}$).

Scale bars represent: 250 μm (**C**), and 25 μm (**A**). Statistical analyses were carried out by the PLSD Fisher test according to the significance of the Student's t-test. Student's t-test: ns, not significant.

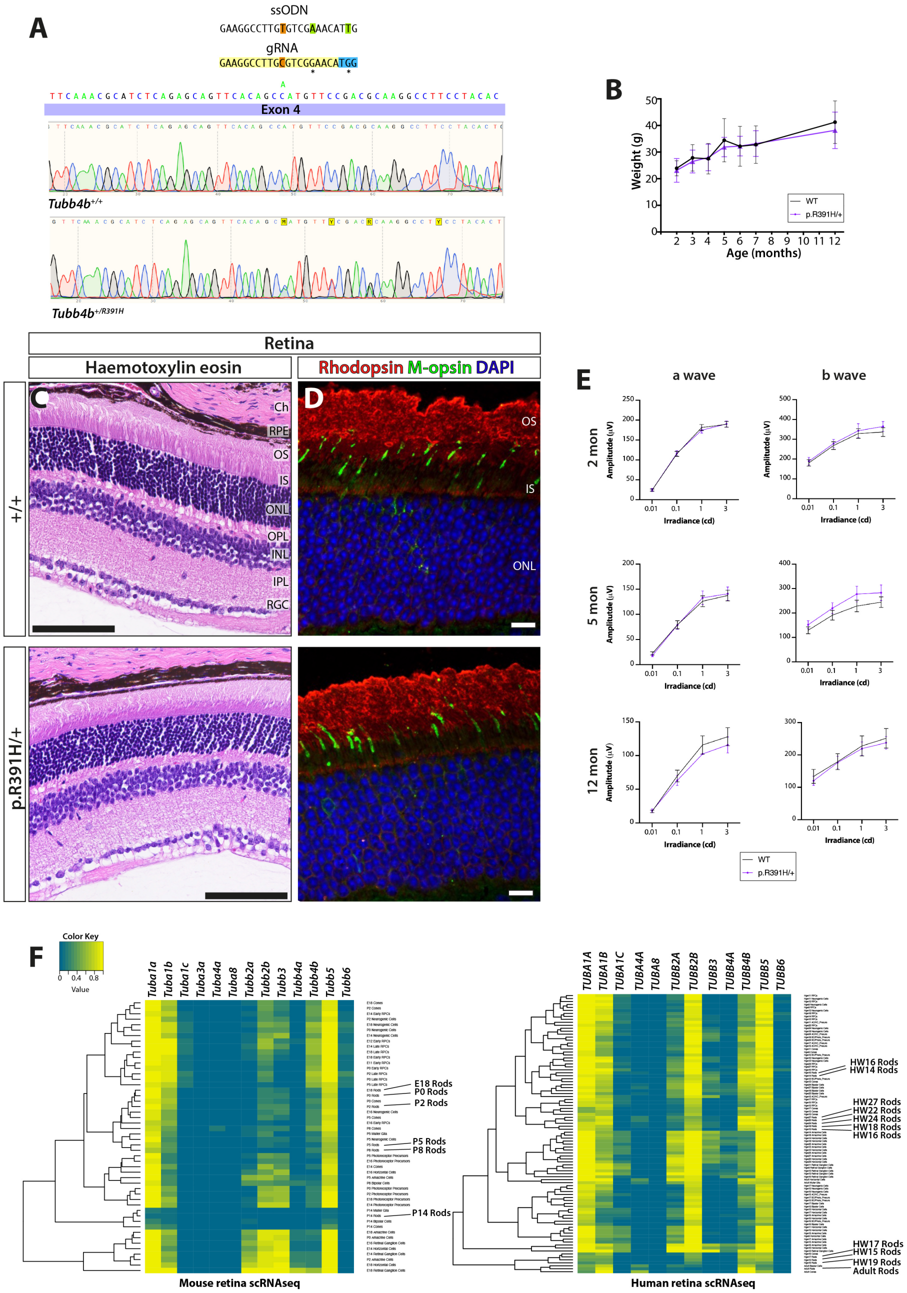

Fig. S10.

Engineered point mutations in *Tubb4b* do not recapitulate patient phenotypes in the mouse retina.

(**A**) Strategy to generate LCA KI *Tubb4b*$^{R391H/+}$ allele by Cas9 genome editing in the 4th exon of *Tubb4b* and Sanger sequencing to validate.

(**B**) *Tubb4b*$^{R391H/+}$ animals show no decrease in fitness or survival postnatally.

(**C-E**) *Tubb4b*$^{R391H/+}$ animals show no early onset or age-related degeneration of photoreceptors by histology (**C**) or immunofluorescence (**D**) (rhodopsin: red; M-opsin: green) in 4 month old adult mice. (**E**) *Tubb4b*$^{R391H/+}$ animals show no evidence of physiological changes in neuroretina with age as shown by electroretinogram unlike the p.R391H/+ human patients.

(**F**) Species-specific differences in expression of TUBB4B in the neuroretina are likely to account for these differences. Heatmaps show the proportions of cells expressing each tubulin (RNA count greater than zero) in each cell type identified in the population from published mouse (left) (*89*) and human (right) (*88*)

neuroretina single cell RNASeq datasets. Rows are clustered on similarity of proportions among cell types (scale 0-1).

Scale bars represent: 100 μm (**C**) and 10 μm (**D**). (**B**,**E**) Graphic bars represent the mean ± SEM derived from N>3 animals per time point.

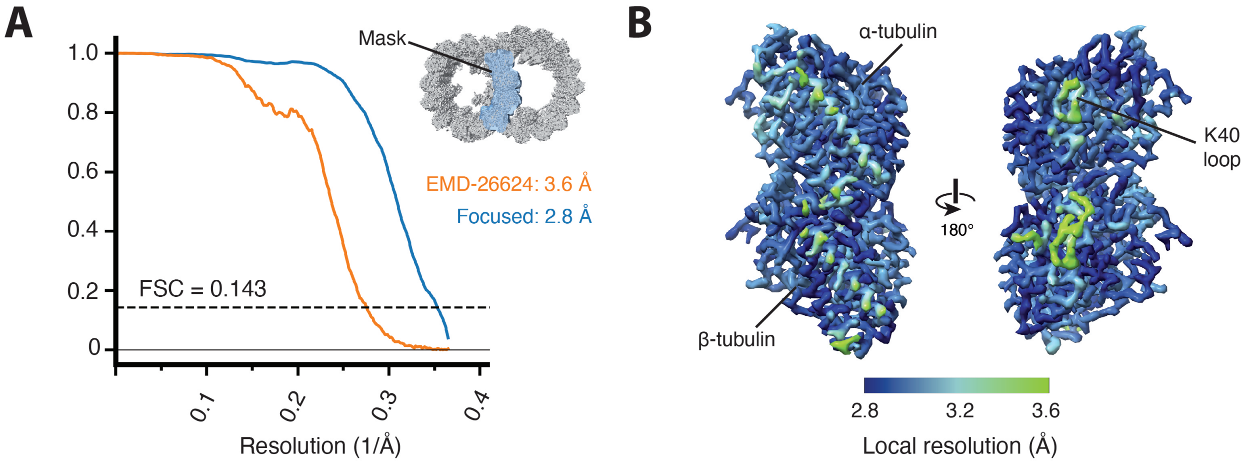

Fig. S11.
Cryo-EM data quality.
(A) Fourier shell correlation (FSC) curves for the deposited map of the human doublet microtubule from respiratory cilia (EMD-26624) and the region after focused refinement (this study). Resolutions are estimated according to the FSC = 0.143 criterion. The inset shows the mask (transparent blue) applied to the ribbon protofilaments between the A and B tubules during focused refinement.
(B) The central tubulin heterodimer from the region masked in (**A**) colored by local resolution.

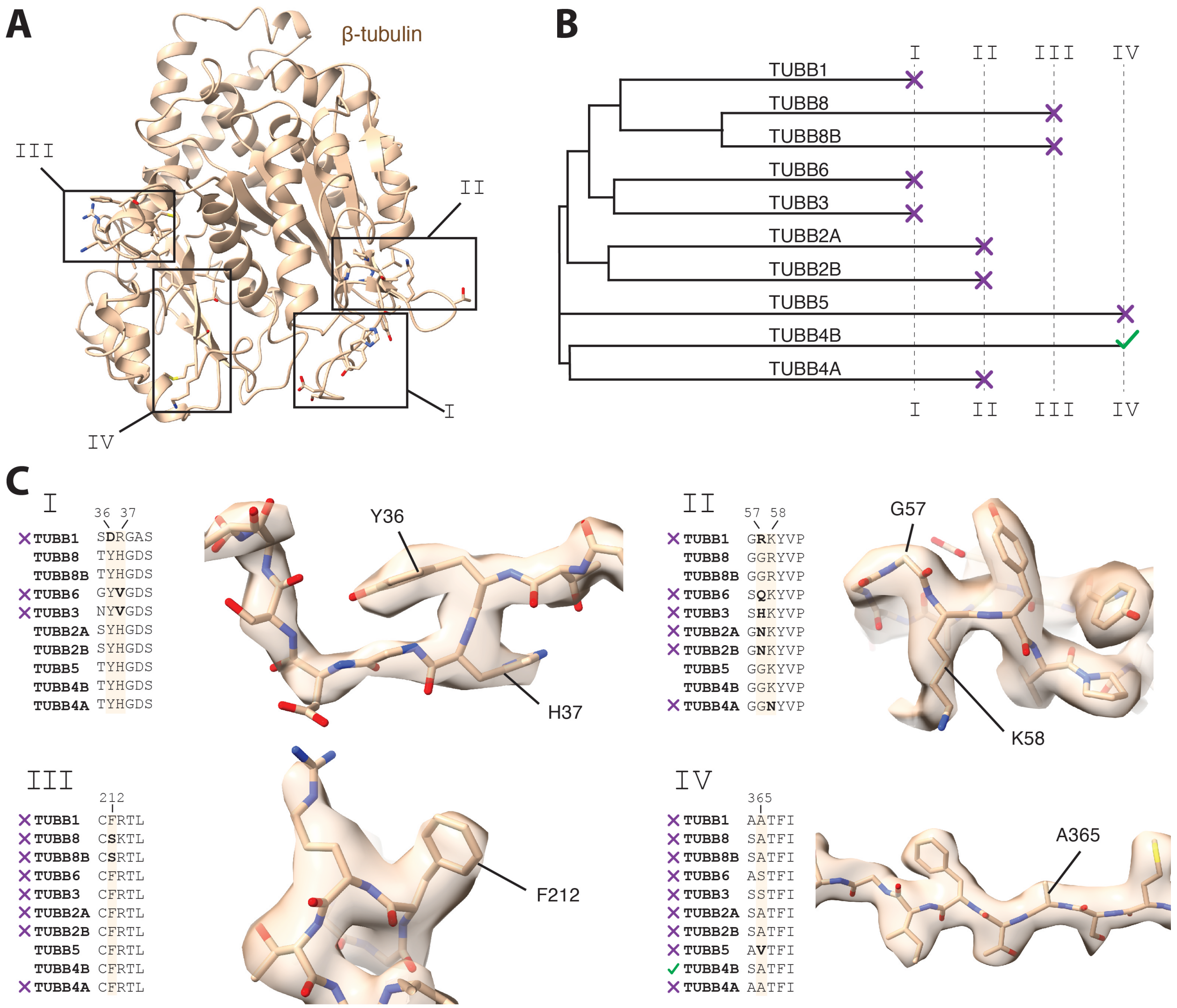

Fig. S12.

Identification of TUBB4B as the predominant β-tubulin isotype of human respiratory cilia axonemes.

(A) Model of β-tubulin highlighting the four regions (I-IV) that allow discrimination of the β-tubulin isotypes in the cryo-EM map.

(B) A hybrid phylogenetic/decision tree showing the outcome when all 10 isotypes of β-tubulin encoded by the human genome were analyzed sequentially for their fit to the cryo-EM density of each region boxed in (**A**). A purple cross indicates that the sequence was incompatible with the density, and the isotype was excluded from the next step. Only TUBB4B is consistent with all 4 regions.

(C) Fit of an atomic model of TUBB4B to the cryo-EM density for regions I-IX. To the left of each panel is a sequence alignment showing which isotypes could be excluded at each step of the evaluation process.

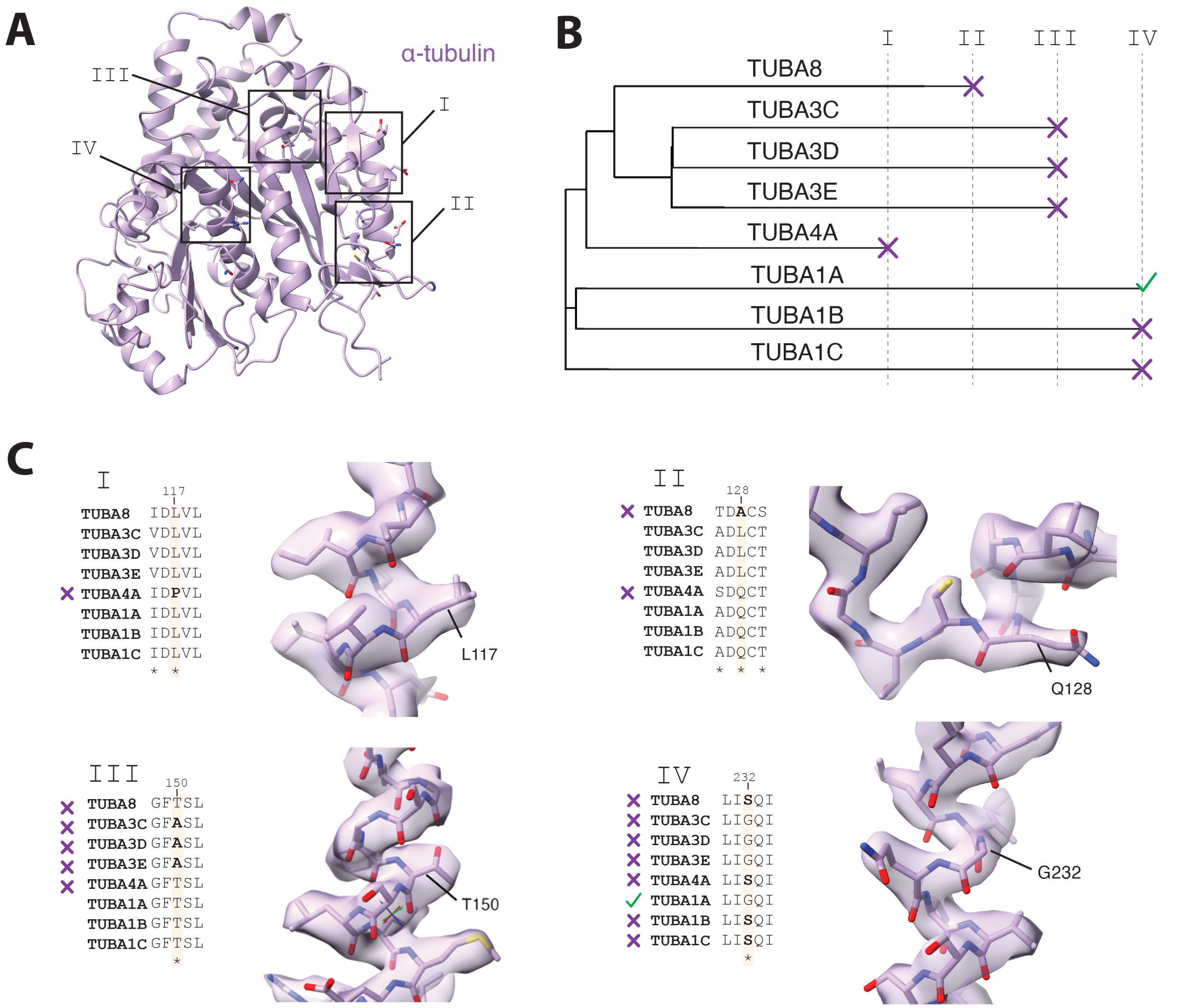

Fig. S13.

Identification of TUB1A1 as the predominant α-tubulin isotype of human respiratory cilia axonemes.
(**A**) Model of α-tubulin highlighting the four regions (I-IV) that allow discrimination of the α-tubulin isotypes in the cryo-EM map. (**B**) A hybrid phylogenetic/decision tree showing the outcome when all 8 isotypes of α-tubulin encoded by the human genome were analyzed sequentially for their fit to the cryo-EM density of each region boxed in (**A**). A purple cross indicates that the sequence was incompatible with the density, and the isotype was excluded from the next step. Only TUBA1A is consistent with all 4 regions. (**C**) Fit of an atomic model of TUBA1A to the cryo-EM density for regions I-IX. To the left of each panel is a sequence alignment showing which isotypes could be excluded at each step of the evaluation process.

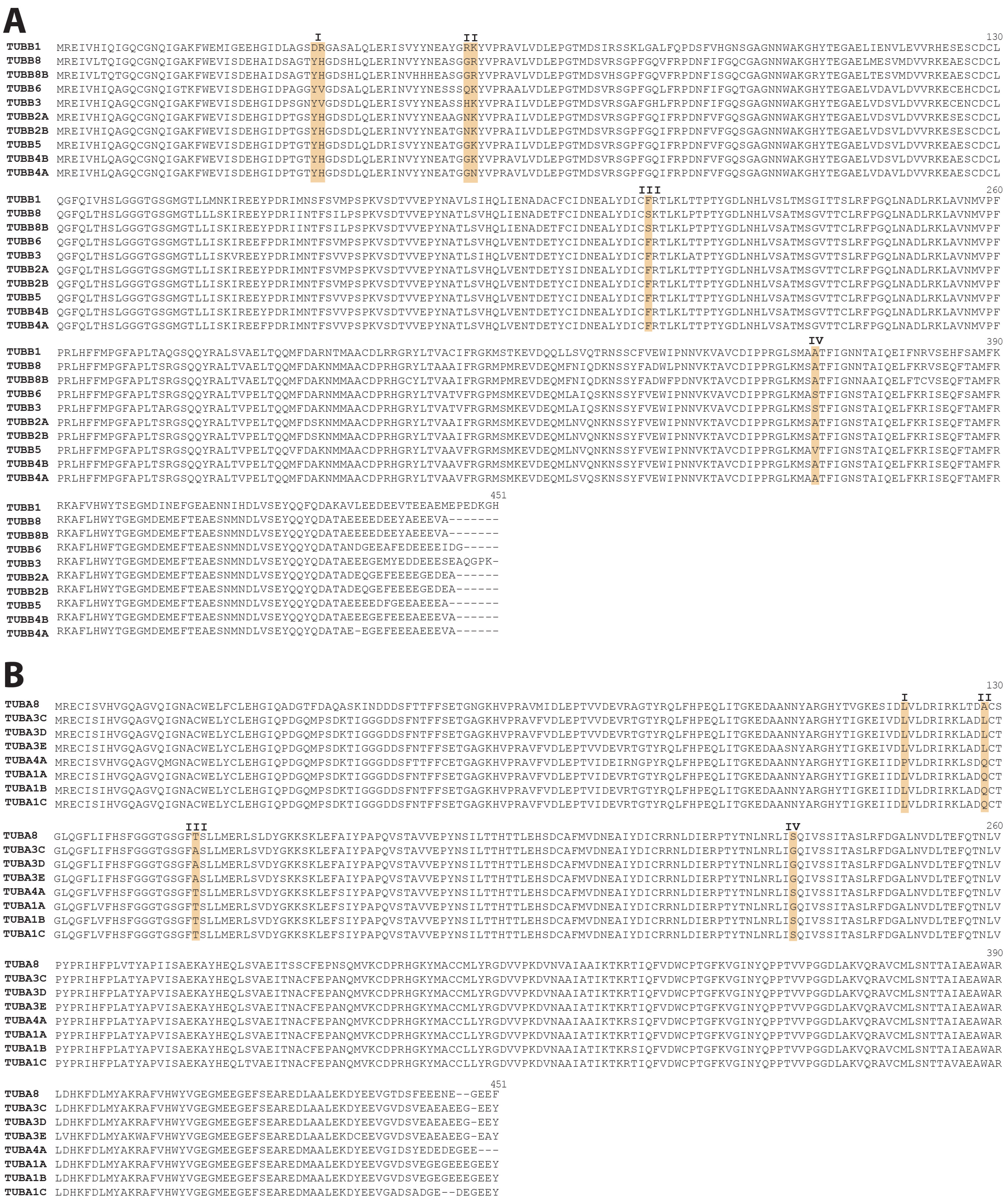

Fig. S14.

Sequence analysis of human α-and β-tubulin isotypes.

(**A**) Multiple sequence alignment of β-tubulin isotypes.

(**B**) Multiple sequence alignment of α-tubulin isotypes. TUBAL3 was omitted from the analysis as it contains a 7-residue insertion at position 39 that is incompatible with the α-tubulin cryo-EM density. In both panels A and B, colored highlights and numerals I-IV denote residues evaluated for their fit to the cryo-EM density in **Fig. S12** and **Fig. S13**, respectively.

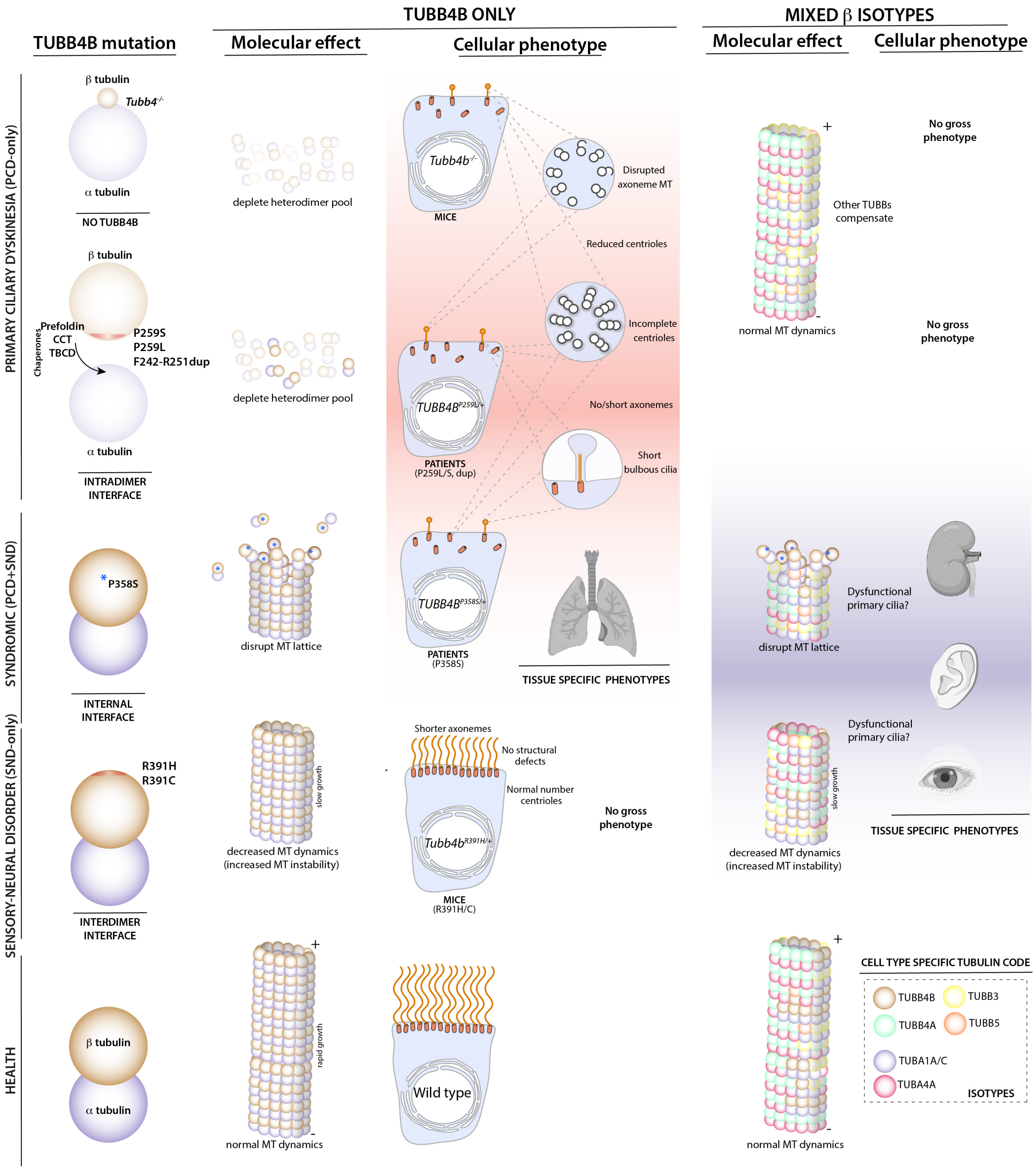

Fig. S15.

Patient *TUBB4B* variants cause distinct clinical phenotypes depending on how they disrupt tubulin heterodimerization, polymerization and microtubule stability.

Pathogenic PCD-variants stall during heterodimerization effectively depleting heterodimer pools; these phenocopy null mutations in airway cells where motile axonemes are enriched in TUBB4B and as such these variants will cause only PCD. Whereas in other cilia where alternative axonemal-competent TUBB isotypes can be incorporated, they overcome the competitive dominant negative effect of TUBB4B PCD-only variants on tubulin heterodimerization, as the ratio of mutant protein is less. Importantly, these PCD-only patient variants on the intradimer surface cause effects which are molecularly distinct from heterozygous loss-of-function (i.e frameshift) mouse allele, which we show lacks a phenotype. In contrast,

p.P358S (on the luminal interface) likely has an assembly-mediated dominant-negative effect on poisoning the lattices into which it integrates, thus disrupting integration of new heterodimers and effective concentration-dependent polymerization during multiciliogenesis to cause PCD. It also means in tissues where TUBB4B is expressed but other isotypes make up axonemes (mixed isotype lattice), p.P358S TUBB4B will integrate causing syndromic PCD+SND disease. The SND-only patient variants are found on the interdimer interface, where we observed slowed growth characteristics in overexpression experiments. They are also predicted to impair stabilization between adjacent dimers resulting in subtle changes in airway cilia length (in mouse) but without gross airway disease but other tissues such as retina and inner ear (as seen in human patients) species-specific sensitivity to dysfunction may exist.

Table S1.

Clinical features of *TUBB4B* cohort reported in this study.

				Primary ciliary dyskinesia (PCD)														Sensorineural disease (SND)					Mutation
Case number	Samples recruited	Sex	Ethnicity	Age at diagnosis	Ciliary ultrastructure	Cilia motility	nNO (ppb)	Current FEV1 (% predicted)	NRD	Chronic wet cough	Regular IV antibiotics	Recurrent infections	Rhinosinusitis	Bronchiectasis	Recurrent ear infection	Hydrocephaly	Situs defects	Auditory Defects	ERG	Renal defects	Congenital heart disease	Skeletal defects	HGVS protein
P1	Trio	F	Caucasian	11	Ciliary agenesis	Abnormal	15.5	47	YES	YES	YES	YES	YES	YES	NO	YES	NO	CHL	Normal	NO	NO	NO	p.P259L
P2	Trio	F	Mixed	2	Ciliary agenesis	NA	50	107	YES	YES	NO	YES	YES	YES	NO	NO	NO	SNHL/CHL	NA	NO	NO	NO	
P3	Trio	F	Caucasian	2	Ciliary agenesis	NA	NA	NA	YES	YES	NA	YES	YES	NA	YES	YES	NO	Hearing deficit	NA	NO	NO	NO	
P4	Family	M	Caucasian	10	Ciliary agenesis	Abnormal	23	76	YES	YES	YES	YES	YES	YES	YES	YES	YES	SNHL/CHL	NA	NO	Dextrocardia	NO	
P5	Singleton	F	Caucasian	Birth	Ciliary agenesis	NA	35	NA	NO	NA	NA	YES	YES	YES	NO	YES	NO	NA	NA	NO	NO	NO	
P6	Family	F	Caucasian	7	Ciliary agenesis	NA	6.9	45	YES	YES	YES	YES	NA	YES	YES	YES	NO	CHL	NA	NO	NO	NO	
P7	Singleton	M	Caucasian	15	NA	NA	NA	NA	YES	NO	YES	YES	NA	YES	YES	NO	NO	SNHL	NA	NO	NO	NO	p.F242_R251dup
P8	Duo	M	Caucasian	4	Ciliary agenesis	Abnormal	6	75	YES	YES	YES	YES	YES	YES	NO	YES	NO	CHL	Normal	NO	No	NO	p.P259S
P9	Trio	M	Caucasian	1.5	Ciliary agenesis	Abnormal	6.7	NA	YES	YES	NO	YES	YES	NO	YES	NO	NO	SNHL/CHL	Flat	NO	NO	NO	p.P358S
P10	Trio	M	Caucasian	14	Ciliary agenesis	Abnormal	NA	NA	NA	NA	NA	YES	YES	NA	NA	NA	NO	SNHL	Flat	Nephromegaly, polyuria, hyperfiltration and hypertension	NO	Short stature, dysmorphic features	
P11	Family	M	African	10	NA	NA	NA	NA	YES	NA	NA	NA	NA	NA	YES	NO	NO	SNHL	Flat	NR	Restrictive cardiomyopathy	NR	
P12	Trio	F	Caucasian	11	Ciliary agenesis	Abnormal	NA	88.4	NA	NO	NO	YES	YES	YES	NO	NO	NO	SNHL	Flat	Kidney dysplasia and mild eGFR reduction	Perimembranotic ventricular septal defect	Short stature	

Table S2.

Genetic features of patient variants in *TUBB4B.*

Case number	Change (hg38)	HGVS cDNA	HGVS protein	Zygosity	Inheritance Mode	ACMG Criteria	Platform used
P1	chr9:137242994:C:T	c.776C>T	p.P259L	Heterozygous	de novo	PS2-PM2- PP2-PP3	WGS: Illumina HiSeq X
P2	chr9:137242994:C:T	c.776C>T	p.P259L	Heterozygous	NA	PM2-PP2-PP3	Capture: Agilent SureSelectQXT NGS kit WES: Illumina NextSeq
P3	chr9:137242994:C:T	c.776C>T	p.P259L	Heterozygous	de novo	PS2-PM2- PP2-PP3	Capture: Agilent SureSelect All Exon V.6 WES: Illumina HiSeq2500
P4	chr9:137242994:C:T	c.776C>T	p.P259L	Heterozygous	de novo	PS2-PM2- PP2-PP3	Capture: Roche MedExome WES: Illumina NextSeq
P5	chr9:137242994:C:T	c.776C>T	p.P259L	Heterozygous	NA	PM2-PP2-PP3	Capture: Roche SeqCap EZ Choice capture WES: Illumina MiSeq
P6	chr9:137242994:C:T	c.776C>T	p.P259L	Heterozygous	de novo	PM2-PS2-PP2-PP3	Capture: Roche xGen Exome v1.0 WES: Illumina Novaseq 6000
P7	chr9:137242934:G:GCCTGCGCTTCCCAGGCCAGCTCAATGCTGA	c.723_752dup	p.F242_R251dup	Heterozygous	NA	PM2-PM4	Capture: Roche xGen Exome v1.0 WES: Illumina Novaseq 6000
P8	chr9:137242993:C:T	c.775C>T	p.P259S	Heterozygous	NA	PM2-PP2-PP3	WGS: Illumina HiSeq 2500
P9	chr9:137243290:C:T	c.1072C>T	p.P358S	Heterozygous	de novo	PS2-PM2-PP2-PP3	WES: GeneDx
P10	chr9:137243290:C:T	c.1072C>T	p.P358S	Heterozygous	de novo	PS2-PM2-PP2-PP3	Capture: Agilent SureSelect All Exon V5 WES: Illumina HiSeq2500 HT
P11	chr9:137243290:C:T	c.1072C>T	p.P358S	Heterozygous	de novo	PS2-PM2- PP2-PP3	WES: GeneDx WGS: Baylor Genetics
P12	chr9:137243290:C:T	c.1072C>T	p.P358S	Heterozygous	de novo	PS2-PM2-PP2-PP3	Capture: Roche MedExome WES: Illumina NextSeq 500

Table S3.

Description of patient variants in *TUBB4B* and predicted pathogenicity. Standard predictors struggle to predict pathogenicity of tubulin variants accurately. Using structural modelling of variant effects on both the β-tubulin monomer (subunit) and its interaction with α-tubulin a heterodimer (full) based on the crystal structure, we could demonstrate profound effects on heterodimer formation in PCD-only variants. Here, higher ΔΔG means the variant is predicted to destabilize the protein and importantly its interactions (full). Interactions, like lateral interactions between subunits not in the structure, are not captured for interfaces affected like p.P358S.

Case numbers	Change (hg38)	HGVS cDNA	HGVS protein	PolyPhen2 HumVar score	SIFT score	Grantham score	ΔΔG subunit (kcal/mol)	ΔΔG full (kcal/mol)	Study
P1-P6	chr9:137242994:C:T	c.776C>T	p.P259L	probably damaging (0.993)	deleterious low confidence(0.02)	moderately conservative (98)	1.44	4.6	This study
P8	chr9:137242993:C:T	c.775C>T	p.P259S	possibly damaging (0.847)	deleterious low confidence(0.01)	moderately conservative (74)	3.14	4.92	
P9-P12	chr9:137243290:C:T	c.1072C>T	p.P358S	possibly damaging (0.551)	deleterious low confidence(0.04)	moderately conservative (74)	2.31	2.22	
Family 1-3	chr9:140137842G>A	c.1172G>A	p.R391H	probably damaging (0.993)	deleterious (0)	conservative (29)	0.99	3.41	Luscan et al 2017
Family 4	chr9:140137841C>T	c.1171C>T	p.R391C	probably damaging (0.999)	deleterious (0)	radical (180)	1.5	2.77	

Table S4.
Statistics for data collection, data processing, model refinement and validation.

	**TUBA1A-TUBB4B heterodimer (EMD-40480) (PDB 8SH7)**
**Data collection**	
Microscope	Titan Krios
Detector	K3
Voltage (keV)	300
Nominal magnification	64,000x
Electron exposure ($e^-/Å^2$)	60
Defocus range set during data acquisition (μm)	-0.8 to -2.0
Pixel size (Å)	1.37
**Data Processing**	
Particles	208,558
Map resolution (Å)	2.8
**Model composition**	
Chains	2
Atoms	6820
Residues	862
Ligands	GDP: 1, GTP: 1 Mg^{2+}:1
**Refinement**	
Initial model used (PDB code)	7UNG
Resolution limit set in refinement (Å)	2.8
Correlation coefficient (CCmask)	0.89
C_{ref} (masked) (Å)	3.1
Root-mean-square deviation (bond lengths) (Å)	0.008
Root-mean-square deviation (bond angles) (Å)	1.223
**Validation**	
MolProbity Score	1.08
Clashscore	2.02
Rotamer outliers (%)	0
Ramachandran (favored) (%)	97.43
Ramachandran (outliers) (%)	0

Table S5.
CRISPR guides and repair templates.

Name	Sequence	Application	Source
Tubb4b Exon4 p.R391H guide	5' - GAAGGCCTTGTGTCGAAACATTG - 3'	CRISPR guide for generation of *Tubb4b*$^{R391H/+}$ mouse	
Tubb4b Exon4 p.R391H patient variant KI ssODN	5' - GAAGGCCTTGCGTCGGAACATGG - 3'	Repair template for generation of *Tubb4b*$^{R391H/+}$ mouse	
Tubb4b Exon4 p.P259L guide	5' - CCATATTTACAGCCAGTTTCCGC - 3'	CRISPR guide for generation of *Tubb4b*$^{P259L/+}$ mouse	GeneArt Precision gRNA synthesis kit
Tubb4b Exon4 p.P259L patient variant KI ssODN	5'- GCTGGGTGAGCTCAGGAACTGTCAGGGCACGGTACTGCTGGCTGCCCCGGCTGGTCAAGGGGGCAAAGCCAGGCATGAAGAAGTGCAGGCGAGGGAAGaGCACCATgTTaACgGCCAGTTTCCGCAGGTCAGCATTTAGCTGGCCAGGGAATCGCAGGCAAGTGGTTACCCCACTCATGGTGGCGGACACTAGATGGTTC - 3'	Repair template for generation of *Tubb4b*$^{P259L/+}$ mouse	IDT
Tubb4b Exon4 p.P259L patient silent mutation ssODN	5' - GCTGGGTGAGCTCAGGAACTGTCAGGGCACGGTACTGCTGGCTGCCCCGGCTGGTCAAGGGGGCAAAGCCAGGCATGAAGAAGTGCAGGCGAGGGAAGGgCACCATgTTaACgGCCAGTTTCCGCAGGTCAGCATTTAGCTGGCCAGGGAATCGCAGGCAAGTGGTTACCCCACTCATGGTGGCGGACACTAGATGGTTC - 3'	Repair template for generation of T*ubb4b*$^{+/+}$ mouse: silent mutation control	IDT
Tubb4b Exon4 p.P259L guide	5' - AAATGCTGACCTGCGGAAAC - 3'	CRISPR guide for generation of *Tubb4b*$^{P259L/+}$ mouse	GeneArt Precision gRNA synthesis kit
Tubb4b Exon4 p.P259L patient variant KI ssODN	5'- CACCCACTTACGGTGACCTGAACCATCTAGTGTCCGCCACCATGAGTGGGGTAACCACTTGCCTGCGATTCCCTGGCCAGCTAAATGCTGATCTCCGCAAGCTGGCTGTAAATATGGTGCTCTTCCCTCGCCTGCACTTCTTCATGCCTGGCTTTGCCCCCTTGACCAGCCGGGGCAGCCAGCAGTACCGTGCCCTGACA - 3'	Repair template for generation of *Tubb4b*$^{P259L/+}$ mouse	IDT
Tubb4b Exon4 p.P259S guide	5' - CCATATTTACAGCCAGTTTCCGC - 3'	CRISPR guide for generation of *Tubb4b*$^{P259S/+}$ mouse	GeneArt Precision gRNA synthesis kit
Tubb4b Exon4 p.P259S patient variant KI ssODN	5'- GCTGGGTGAGCTCAGGAACTGTCAGGGCACGGTACTGCTGGCTGCCCCGGCTGGTCAAGGGGGCAAAGCCAGGCATGAAGAAGTGCAGGCGAGGGAAGGaCACCATgTTaACgGCCAGTTTCCGCAGGTCAGCATTTAGCTGGCCAGGGAATCGCAGGCAAGTGGTTACCCCACTCATGGTGGCGGACACTAGATGGTTC - 3'	Repair template for generation of *Tubb4b*$^{P259S/+}$ mouse	IDT
Tubb4b Exon4 p.P259S patient silent mutation ssODN	5' - GCTGGGTGAGCTCAGGAACTGTCAGGGCACGGTACTGCTGGCTGCCCCGGCTGGTCAAGGGGGCAAAGCCAGGCATGAAGAAGTGCAGGCGAGGGAAGGgCACCATgTTaACgGCCAGTTTCCGCAGGTCAGCATTTAGCTGGCCAGGGAATCGCAGGCAAGTGGTTACCCCACTCATGGTGGCGGACACTAGATGGTTC - 3'	Repair template for generation of T*ubb4b*$^{+/+}$ mouse: silent mutation control	IDT
Tubb4b Exon4 p.P358S guide	5' - GCCGACATTTTCAGGCCCCG - 3'	CRISPR guide for generation of *Tubb4b*$^{P358S/+}$ mouse	GeneArt Precision gRNA synthesis kit
Tubb4b Exon4 p.P358S patient variant KI ssODN	5'- GTCGGAACATGGCTGTGAACTGCTCTGAGATGCGTTTGAACAGCTCCTGAATAGCGGTGCTGTTGCCAATGAAGGTGGCCGACATTTTCAGGCCCCGAGATGGAATGTCACAGACAGCTGTCTTCACATTGTTGGGGATCCACTCAACAAAGTAGCTGCTGTTCTTGTTTTGGACATTAAGCATCTGTTCGTCCACCTCC - 3'	Repair template for generation of *Tubb4b*$^{P358S/+}$ mouse	IDT
Tubb4b Exon4 p.P358S patient silent mutation ssODN	5' - GTCGGAACATGGCTGTGAACTGCTCTGAGATGCGTTTGAACAGCTCCTGAATAGCGGTGCTGTTGCCAATGAAGGTGGCCGACATTTTgAGcCCgCGAGgTGGAATGTCACAGACAGCTGTCTTCACATTGTTGGGGATCCACTCAACAAAGTAGCTGCTGTTCTTGTTTTGGACATTAAGCATCTGTTCGTCCACCTCC - 3'	Repair template for generation of T*ubb4b*$^{+/+}$ mouse: silent mutation control	IDT
Tubb4b Exon4 KO guide	5' - CCATATTTACAGCCAGTTTCCGC - 3'	CRISPR guide for generation of *Tubb4b*$^{+/-}$ mouse, generated while targeting p.P259L and p.P259S mutations	GeneArt Precision gRNA synthesis kit

Tubb4b Exon1 ALFA guide	5' - CCGCAGTCGCCGCCGCCATG – 3'	CRISPR guide to insert ALFA tag into the N terminus of the *Tubb4b* gene.	GeneArt Precision gRNA synthesis kit
Tubb4b Exon1 ALFA tag ssODN	5'CGCCTGCTCCTCCCGAAGTGCTCCTCTTCTACAGCTGTTCCGCAGTCGCCGCCGCCATGAGCAGGCTGGAGGAGGAGCTGAGGAGGAGGCTGACCGAGCCCGGCGGCAGCGGCATCGATGGCGGCAGCGGCTCCGGAAGGGAGATCGTGCACCTGCAGGCTGGGCAGTGCGGCAACCAGATTGGCGCCAAGGTAGG-3'	Repair template to generate N-terminally tagged *Tubb4b*$^{ALFA/+}$ mouse	IDT

Table S6.

Site directed mutagenesis and genotyping primers.

Site mutagenesis primers			
**Patient variant**	**Primer name**	**Primer Sequence**	**Process**
**P259L**	p.P259L_forward	5' CTGTGAACATGGTCC**T**GTTTCCCCGGCTGCA 3'	Site directed mutagenesis
	p.P259L_reverse	5' TGCAGCCGGGGAAAC**A**GGACCATGTTCACAG 3'	
**P259S**	p.P259S_forward	5' GCTGTGAACATGGTC**T**CGTTTCCCCGGCTGC 3'	Site directed mutagenesis
	p.P259S_reverse	5' GCAGCCGGGGAAACG**A**GACCATGTTCACAGC 3'	
**P358S**	p.P358S_forward	5' CTGTCTGTGACATCCCA**T**CTCGGGGGCTAAAAAT 3'	Site directed mutagenesis
	p.P358S_reverse	5' ATTTTTAGCCCCCGAG**A**TGGGATGTCACAGACAG 3'	
**R391C**	p.R391C_forward	5' CACGGCCATGTTCCGG**T**GCAAGGCCTTCCTGCAC 3'	Sitedirected mutagenesis
	p.R391C_reverse	5' GTGCAGGAAGGCCTTGC**A**CCGGAACATGGCCGTG 3'	
**R391H**	p.R391H_forward	5' CACGGCCATGTTCCGGC**A**CAAGGCCTTCCTGCAC 3'	Site directed mutagenesis
	p.R391H_reverse	5' GTGCAGGAAGGCCTTG**T**GCCGGAACATGGCCGTG 3'	
**Genotyping primers**			
**Mouse Line**	**Primer name**	**Primer Sequence**	**Process**
*Tubb4b^R391H^*	Tubb4b_R391H F	5' CTGAAAATGTCGGCCACCT 3'	PCR followed by Sanger sequencing
	Tubb4b_R391H R	5' GACTAAGACAGCTCTAAGCC 3'	
*Tubb4b^KO^*	Tubb4b KO F	5' GTTGAGCCCTACAATGCCAC 3'	PCR followed by Sanger sequencing
	Tubb4b KO R	5' GAAGGTGGCCGACATTTTCA 3'	
*Tubb4b^ALFA^*	Tubb4b ALFA F	5’ GGGCGGGTCATAAGAGGTAT 3’	PCR
	Tubb4b ALFA R	5’ CGCAGAAACCTAGCCTCCTA 3’	

Table S7.

Primary antibodies and small molecules.

Antigen	Antibody/Clone Name/RRID	Host species	Source	Application
α-tubulin	DM1A, T9026, RRID:AB_477593	Mouse	Sigma	WB (1:1000); IF (1:2000, MeOH)
α-tubulin	YL1/2, ab6160, RRID:AB_305328	Rat	Abcam	WB (1:2000); IF (1:1000, PFA)
Acetylated α-tubulin	6-11B-1, T6793, RRID:AB_477585	Mouse	Sigma	IF (1:1000-1:5000)
ALFA-Atto647N	N1502-At647N-L	Alpaca	Nanotag	IF (1:1000)
ALFA-HRP	N1505-HRP	Camelid nanobody	Nanotag	WB (1:1000)
ARL13B	17711-1-AP, RRID:AB_2060867	Rabbit	Proteintech	IF (1:1000, PFA)
Centrin	20H5 04-1624, RRID:AB_10563501	Mouse	Merck	IF (1:500, MeOH w. PE or PFA)
Detyrosinated tubulin	AA12, ab254154	Mouse	Abcam	IF (1:1000, PFA)
DNALI1	HPA028305, RRID:AB_10601807	Rabbit	Sigma	IF (1:200, PFA)
DNALI1	N-13, RRID:AB_2246230	Goat	Santa Cruz	IF (1:75, PFA)
DNAH5	HPA037469, RRID:AB_10672791	Rabbit	Sigma	IF (1:200, PFA)
DNAI2	IC8, H00064446-M01, RRID:AB_426059	Mouse	Abnova	IF(1:100, PFA)
EB1	610534, 5/EB1, AB_397891	Mouse	BD Biosciences	IF (1:200, MeOH)
FLAG	ab95045, RRID:AB_10676074	Goat	Abcam	IF (1:5000, MeOH)
FLAG	M2, F1804, RRID:AB_262044	Mouse	Sigma	WB (1:1000)
FOP	11343-1-AP, RRID:AB_2103362	Rabbit	Proteintech	IF (1:500, PFA)
Gamma tubulin	GTU88, T6557, RRID:AB_477584	Mouse	Sigma	IF (1:500, MeOH w PE)
GFP	MMS-118P, RRID:AB_10063778	Mouse	Covance	WB (1:5000)
GFP-Booster Alexa 488	AB_2827573	Alpaca	Proteintech, Chromotek	IF (1:500-1,000, PFA)
His	102-10667	Rabbit	RayBiotech	WB (1:1000)
IFT88	13967-1-AP, RRID:AB_2121979	Rabbit	Proteintech	IF (1:100, PFA)
M-Opsin	OSR00222W, RRID:AB_2178929	Rabbit	ThermoFIsher Scientific	IF (1:500, PFA)
Polyglutamylated tubulin	GT335, AG-20B-0020-C100,	Mouse	AdipoGen Life Sciences	IF (1:1000, PFA or MeOH)
Pericentrin	ab4448, RRID:AB_304461	Rabbit	Abcam	IF (1:1000, MeOH)
Rhodopsin	4D2, NBP2-59690	Mouse	Novus Biologicals	IF (1:500, PFA)
TBCD	14867-1-AP, RRID:AB_2199548	Rabbit	Proteintech	WB (1:1000)

Table S8.
Secondary antibodies.

Antigen	Host Species	Dilution	Source	Application
ECL α-Mouse IgG, HRP-conjugated	Sheep	1:7500	GE Healthcare UK Ltd	WB
ECL α-Rabbit IgG, HRP-conjugated	Sheep	1:7500	GE Healthcare UK Ltd	WB
HRP-conjugated α-Rabbit IgG H + L	Goat	1:5000	BioRad	WB
HRP-conjugated α-Mouse IgG H + L	Goat	1:5000	BioRad	WB
Alexa 488, 568, 594, 647-conjugated-α-Mouse	Donkey	1:500	Invitrogen Molecular Probes	IF
Alexa 488, 594, 647 -conjugated-α-Rabbit	Donkey	1:500	Invitrogen Molecular Probes	IF
Alexa 555, 647-conjugated-α-Goat	Donkey	1:500	Invitrogen Molecular Probes	IF

Movie S1.
Tomogram from TEM samples of PCD-only patient (P3) showing distended cilium without clear axonemal microtubules adjacent to one where axonemal structure is preserved (12 fps).

Movie S2.
Nasal epithelial cell culture from healthy female control en-face view (30 fps).

Movie S3.
Nasal epithelial cell culture from healthy female control side view showing coordinated metachronal beat (12 fps).

Movie S4.
Nasal epithelial cell culture from PCD-only patient (P8) en-face view showing paucity of short, bulbous cilia and disrupted beat (30 fps).

Movie S5.
Nasal epithelial cell culture from PCD-only patient (P8) side view showing sparse short cilia with slow beat (12 fps).

Movie S6.
Nasal epithelial cell culture from PCD-only patient (P1) side view showing sparse short cilia with slow dyskinetic beat (12 fps).
